# Decreased TMIGD1 aggravates colitis and intestinal barrier dysfunction via the BANF1-NF-κB pathway in Crohn’s disease

**DOI:** 10.1186/s12916-023-02989-2

**Published:** 2023-08-04

**Authors:** Longyuan Zhou, Liguo Zhu, Xiaomin Wu, Shixian Hu, Shenghong Zhang, Min Ning, Jun Yu, Minhu Chen

**Affiliations:** 1 Department of Gastroenterology, The First Affiliated Hospital of Sun Yat-Sen University, Guangzhou, Guangdong People’s Republic of China; 2 Institute of Precision Medicine, The First Affiliated Hospital of Sun Yat-Sen University, Guangzhou, Guangdong People’s Republic of China; 3 State Key Laboratory of Digestive Disease, Institute of Digestive Disease and The Department of Medicine and Therapeutics, Li Ka Shing Institute of Health Sciences, CUHK Shenzhen Research Institute, The Chinese University of Hong Kong, Hong Kong SAR, China

**Keywords:** Crohn’s disease, TMIGD1, BANF1, Intestinal epithelial barrier, NF-κB pathway

## Abstract

**Background:**

Disrupted intestinal epithelial barrier is one of the major causes of Crohn’s disease (CD). Novel molecular targets for intestinal epithelial barrier are essential to treatment of CD. Transmembrane and immunoglobulin domain-containing protein 1 (TMIGD1) is an adhesion molecule that regulates cell adhesion, migration, and enterocyte differentiation. However, the function and mechanism of TMIGD1 in CD and intestinal epithelial barrier has rarely been studied. Furthermore, the association between TMIGD1 and the clinical features of CD remains unclear.

**Methods:**

Transcriptome analysis on colonic mucosa from CD patients and healthy individuals were performed to identify dysregulated genes. Multi-omics integration of the 1000IBD cohort including genomics, transcriptomics of intestinal biopsies, and serum proteomics identified the association between genes and characteristics of CD. Inflammation was assessed by cytokine production in cell lines, organoids and intestinal-specific *Tmigd1* knockout (*Tmigd1*^*INT-KO*^) mice. Epithelial barrier integrity was evaluated by trans-epithelium electrical resistance (TEER), paracellular permeability, and apical junction complex (AJC) expression. Co-immunoprecipitation, GST pull-down assays, mass spectrometry, proteomics, and transcriptome analysis were used to explore downstream mechanisms.

**Results:**

Multi-omics integration suggested that TMIGD1 was negatively associated with inflammatory characteristics of CD. TMIGD1 was downregulated in inflamed intestinal mucosa of patients with CD and mice colitis models. *Tmigd1*^*INT-KO*^ mice were more susceptible to chemically induced colitis. In epithelial cell lines and colonic organoids, TMIGD1 knockdown caused impaired intestinal barrier integrity evidenced by increased paracellular permeability and reduced TEER and AJC expression. TMIGD1 knockdown in intestinal epithelial cells also induced pro-inflammatory cytokine production. Mechanistically, TMIGD1 directly interacted with cytoplasmic BAF nuclear assembly factor 1 (BANF1) to inhibit NF-κB activation. Exogenous expression of TMIGD1 and BANF1 restored intestinal barrier function and inhibited inflammation *in vitro* and *in vivo*. TMIGD1 expression predicted response to anti-TNF treatment in patients with CD.

**Conclusions:**

Our study demonstrated that TMIGD1 maintained intestinal barrier integrity and inactivated inflammation, and was therefore a potential therapeutic target for CD.

**Supplementary Information:**

The online version contains supplementary material available at 10.1186/s12916-023-02989-2.

## Background

Crohn’s disease (CD) is a chronic inflammatory gastrointestinal disease that often causes strictures or fistulas in the terminal ileum, colon, and perianal regions [1, 2]. The intestinal mucosal barrier plays a crucial role in the development of CD through maintaining intestinal homeostasis, and consists of the epithelial layer, mucus layer, mucosal immunity, and commensal microbiota [3, 4]. The epithelial layer is the cornerstone of intestinal homeostasis due to its involvement in physically separating the internal milieu from the lumen, modulating mucus secretion, microbiota-host communication, and adjusting immune responses via diverse epithelial cell subtypes [5]. Epithelial barrier integrity defects are mainly characterized by apical junction complex (AJC) damage, including tight junctions, adhesion junctions, and desmosomes [6]. Following inflammatory injury, the dysregulation of AJC proteins increases gut permeability and therefore aggravates colitis [7]. However, further investigation is required to develop novel molecular targets to recover epithelial integrity and alleviate inflammation.

Transmembrane and immunoglobulin domain-containing 1 (TMIGD1), consisting of two extracellular immunoglobulin (Ig) domains, one transmembrane domain, and one intracellular domain, is transported from cytoplasm to intercellular junction [8]. TMIGD1 is a component of the brush border and localizes to the proximal base region of microvilli in intestinal epithelial cells [9]. TMIGD1 functions as an adhesion receptor and a tumor suppressor [10]. Recruited by EBP50 and E3KARP in the brush border, TMIGD1 maintains microvilli formation. TMIGD1 binds to cytoskeletal-linking proteins, such as moesin and ezrin, and regulates cell adhesion and migration [11, 12]. TMIGD1 also regulates transepithelial permeability and protects renal tubular epithelial cells from oxidative injury [8]. Additionally, TMIGD1 displays a declining trend in normal intestinal mucosa, nonpolypoid lesions, polypoid lesions, and colon cancer [13]. TMIGD1 induces cell cycle arrest at the G2/M phase and enterocyte differentiation; TMIGD1 deficiency impairs the maturation of intestinal epithelia and leads to a deformed apicobasal organization in mouse microvilli [13, 14]. Although TMIGD1 expression has been reported to decrease significantly in CD mucosa [15], the function and mechanism of TMIGD1 in CD and intestinal barrier has rarely been studied. Furthermore, the association between TMIGD1 and the clinical features of CD remains unclear.

In this study, we performed transcriptome analysis on colonic mucosa from in-house patients and multi-omics integration using the 1000IBD cohort dataset, including genomics, transcriptomics of intestinal biopsies, and serum proteomics. We found that downregulation of TMIGD1 was associated with more severe inflammatory characteristics in CD. We also generated intestinal-specific Tmigd1 knockout (*Tmigd1*^*INT-KO*^) mice, cell lines, and colonic organoids to demonstrate that decreased TMIGD1 promoted inflammation and triggered barrier dysfunction. Moreover, we unveiled a novel molecular mechanism: direct interaction between TMIGD1 and cytoplasmic BAF nuclear assembly factor 1 (BANF1) inhibits NF-κB pathway.

## Methods

### Human samples

The human study was approved by the Ethics Committee Institutional Board of the First Affiliated Hospital of Sun Yat-sen University, and informed consent was also obtained from all participants ([2022] No. 252). All human samples were collected by endoscopic biopsy from the Department of Gastroenterology, the First Affiliated Hospital of Sun Yat-sen University. The diagnosis of CD was based on clinical, radiologic, endoscopic, and histological examinations according to the European Crohn’s and Colitis Organization guidelines [16]. Crohn’s disease activity index (CDAI), simple endoscopic score for Crohn's disease (SES-CD), and Geboes histology activity score (GHAS) were assessed according to protocols [17–19].

### 1000IBD cohort dataset

The 1000IBD cohort dataset (EGAS00001002702) was based on a well-established inflammatory bowel disease cohort from the University Medical Center Groningen containing 1215 patients [20]. The genomic information was generated by Global Screening Array and whole-exome sequencing for all patients. The intestinal transcriptomic data was detected using bulk mRNA sequencing for 171 patients. Serum inflammatory biomarkers were measured by Olink Proximity Extension Assay for 1026 patients. To explore the genetic background of *TMIGD1* and its putative effects on downstream molecular traits, we retrieved 831 genomic variants with minor allele frequency >5% located ±1 Mb around the *TMIGD1* gene center. These variants were selected to detect regulatory effects on gene expression, which were defined as *cis*-eQTL [21]. Genomic variants were also used to determine their associations with inflammatory biomarkers (protein quantitative trait loci, pQTL) [22]. The patients with only reference alleles were coded as 0 while heterozygous and homozygotes of alternative alleles were coded as 1 and 2, respectively. All the QTL effects were assessed using generalized linear models according to our previous studies [21, 22].

### Mice and colitis models

All animal experimental protocols were approved by the Ethics Committee Institutional Board of the First Affiliated Hospital of Sun Yat-sen University ([2021] No. 028). All mice had C57BL/6J background. *Tmigd1*-floxed without Villin-Cre mice (control mice, WT) were intercrossed with Villin-Cre mice to generate *Tmigd1*^*INT-KO*^ mice (Shanghai Model Organisms Company, China). The mice were kept separately after weaning at four weeks of age. Mice were raised at 23℃ ± 3℃ and 35% ± 5% humidity under a 12-h dark/light cycle in the specific pathogen-free facility. Acute models of colitis were induced by administering either 2.5% Dextran sulfate sodium (DSS) (160110, MP Biomedicals, USA) through drinking water or 2,4,6-trinitrobenzenesulfonic acid (TNBS) (P2297, Sigma-Aldrich, USA) through an intra-rectal enema to 6–8-week-old mice, as described by Wirtz et al. [23]. The disease activity index (DAI) was determined using weight loss, stool consistency, and intestinal bleeding. Endoscopic scores and histological scores for DSS- and TNBS-induced colitis were calculated according to the classic protocol [23]. Adenoviruses for Tmigd1 and Banf1 overexpression and control viruses were administered to mice by intraperitoneal injection.

### Cell culture

Caco2, a colonic epithelial adenocarcinoma cell line, was purchased from the American Tissue Culture Collection (ATCC, USA) and cultured in Dulbecco’s modified Eagle’s medium (DMEM; C11995500BT, Gibco, USA) supplemented with 10% fetal bovine serum (10099-141C, Gibco, USA) and 1% antibiotics (15140-122, Invitrogen, USA). NCM460, a colonic epithelial cell line, was purchased from Incell Corporation LLC (INCELL, USA) and cultured in M3: BaseF medium (M300F, INCELL, USA) supplemented with 10% fetal bovine serum and 1% antibiotics. Cells were grown in a humidified incubator with 5% CO_2_ at 37°C for no longer than 2 months.

### Organoid isolation and culture

Crypt isolation and cultivation were performed as described by Lukonin et al. [24]. Colon tissues were dissected, washed with ice-cold phosphate buffered saline (PBS; BL601A, Biosharp, CHN), and cut into small pieces. Villi were completely washed off with ice-cold PBS by pipetting up and down until the supernatant became clear. Tissue pieces were incubated in the Gentle Cell Dissociation Reagent (#100-0485, Stemcell Technologies, CAN) for either 30 min (human samples) or 15 min (mouse samples) with agitation. After vigorous pipetting, filtering through a 70-μm cell strainer (352350, Corning, USA), and washing, colonic crypts were collected and seeded into Matrigel (356231, Corning, USA) supplemented with IntestiCult™ organoid growth medium (06005 and 06010, Stemcell Technologies, CAN). Organoids were cultured in 5% CO_2_ at 37°C for at least seven days before use. The medium was replaced every 2 days, and organoids were passaged once a week.

### Western blotting

Cells and tissues were lysed using lysis buffer (20-188, MERCK, GER) supplemented with protease and phosphatase inhibitor cocktail (5872, CST, USA). Protein samples were subjected to SDS-PAGE and transferred to PVDF membranes. After incubation with primary antibodies at 4°C overnight, the membranes were washed and incubated with horseradish peroxidase (HRP)-conjugated secondary antibodies at 25℃ for 2 h. The blots were visualized using the iBright™ CL1500 system (Thermo Scientific, USA). Antibodies used in this study are listed in Additional file 1: Table S1.

### Immunochemistry staining

Fresh colon tissues were fixed in 4% paraformaldehyde (P1110, Solarbio, CHN) and embedded in paraffin. The sections were incubated with primary antibodies overnight at 4°C, and then HRP-conjugated secondary antibodies for 30 min at 25℃. Antibodies used in this study are listed in Additional file 1: Table S1. Subsequent detection was performed by substrate detection using DAB (8059, CST, USA). Images were taken using a BX51 microscope (Olympus, JPN), and AxioScan Z1 system (Zeiss, GER).

### Immunofluorescence staining

Paraffin-embedded sections of colon tissues were hydrated in xylene and gradient ethanol and then blocked with goat serum. Sections were incubated with primary antibodies overnight at 4°C, followed by secondary antibodies, and preserved in anti-fade mounting medium supplemented with DAPI (P36981, Invitrogen, USA). Fluorescence was detected using an LSM780 confocal microscope (Zeiss, GER). Antibodies used in this study are listed in Additional file 1: Table S1.

### Quantitative real-time polymerase chain reaction (qPCR)

Total RNA was extracted from either cells or tissues using TRIzol (15596026, Thermo Scientific, USA). Total RNA of the DSS-induced colitis model was purified with lithium chloride to avoid possible inhibition on qPCR by DSS [25]. The Transcript First Stand cDNA Synthesis Kit (04897030001, Roche, Basel, Switzerland) was used for reverse transcription, and Fast Start Universal SYBR Green (12239264001, Roche, Basel, Switzerland) was used for qPCR. The relative quantification of mRNA expression was performed using the delta-delta Ct (ΔΔCt) method. The primer sequences are listed in Additional file 1: Table S2.

### Transmission electron microscopy

Pieces of colonic tissue were pre-fixed overnight at 4 °C and then post-fixed with 1% OsO_4_ for 2 h at 25℃. After dehydration and embedding in epoxy resin, resin blocks were cut to 60–80 nm thickness on an ultramicrotome (Leica UC7, GER) and fished onto 150 mesh cuprum grids with formvar film. The grids were further contrasted using 2% uranyl acetate and 2.6% lead citrate. Finally, cuprum grids were observed under transmission electron microscopy (TEM) (HITACHI HT7800, JPN) operating at 80.0 kV. Examinations of the length of microvilli were performed on a total of 30 longitudinally sectioned microvilli per specimen. Examinations of AJC gaps were performed on a total of 10 longitudinally sectioned junctional regions of colonic epithelial cells per specimen.

### Measurement of transepithelial electrical resistance

Transepithelial electrical resistance (TEER) represents paracellular permeability of the Caco2 monolayer cell model. Caco2 cells were seeded in the upper chamber of a 12-well, 0.4-μm transwell chamber (3460, Corning, USA) at a density of 5×10^4^ cells/well. After 2 weeks, TEER was measured using a Millicell ERS-2 (Millipore, GER).

### Fluorescein isothiocyanate-dextran permeability assay

For mice, 4 kD fluorescein isothiocyanate-dextran (FD4; 60842-46-8, Sigma, USA) powder was dissolved in PBS to obtain an 80 mg/mL solution. Mice were fasted for 4 h prior to gavage with 150 μL of FD4 solution. Three hours after gavage, the mice were anesthetized and their blood was collected and kept in the dark. The leakage of FD4 into the circulation was determined by measuring serum fluorescence (λex/λem= 485/535 nm; TECAN Infinite F500, AUT). For cells, FD4 powder was dissolved in Opti-MEM (31985070, Gibco, USA) to obtain a 2 mg/mL solution. FD4 solution was added to the upper transwell chamber, and Opti-MEM was added to the lower chamber. After incubation for 4 h, the fluorescence intensity from the lower chamber was detected (λex/λem= 485/535 nm; TECAN Infinite F500, AUT). For organoids, FD4 was added to the culture medium as described by Xu et al. [26]. Fluorescence intensity inside the organoid was quantified using an LSM780 confocal microscope (Zeiss, GER).

### Overexpression constructs and shRNA knockdown

Overexpression plasmids for FLAG-tagged TMIGD1, BANF1, and their controls were designed and synthesized by OBiO Technology Corp., Ltd. (Shanghai, CHN). RNAi plasmids for TMIGD1, BANF1, and their controls were purchased from Shanghai GENECHEM Corp., Ltd. (Shanghai, CHN). Plasmid transfection was performed using Lipofectamine™ 3000 (L3000001, Thermo Scientific, USA) according to the manufacturer’s instructions. Overexpression and RNAi lentiviruses for TMIGD1, BANF1, and their controls were synthesized by the two aforementioned companies. The efficacy of the infection was evaluated using qPCR and western blotting. The target sequences were as follows:

TMIGD1 knockdown: CGTGAGATGACAAGTTCTGTT;

BANF1 knockdown: CTTCGGATGCCTTCGAGAGTG;

### Immunoprecipitation

Immunoprecipitation was performed according to the manufacturer’s instructions (90409, Thermo Scientific, USA). After preparation of cell lysate, specific antibody and control IgG were added separately to each aliquot, and samples were rotated at 4°C overnight. The antigen-antibody complex was then bound to Protein A/G magnetic beads for 1 h at 25℃. The magnetic bead-antibody-antigen complex was then washed repeatedly. Finally, the immunocomplex supernatants were eluted and subjected to subsequent analysis.

### GST pull-down assays

GST pull-down assays were performed according to the manufacturer’s protocol (21516, Thermo Scientific, USA). Purified recombinant GST-tagged TMIGD1 protein (TMIGD1-GST; H00388364-P01, Abnova, CHN) was incubated with Glutathione Agarose at 4°C for 2 h. After washing five times, the Glutathione Agarose was incubated with FLAG-tagged BANF1 protein (BANF1-Flag; TP303270, Origene, CHN) at 4°C for 2 h. After washing five times, the elution was analyzed via western blotting.

### Nuclear and cytoplasmic extraction

The nuclear and cytoplasmic fractions were separately extracted using the Minute™ Cytoplasmic and Nuclear Fractionation Kit (SC-003, Invent Biotechnologies, USA). β-Actin was used as the cytoplasmic control, and Lamin B1 was used as the nuclear control for western blotting analysis. Nuclear p65 DNA-binding activity was detected using NF-κB p65 transcription factor assays (ab210613, Abcam, UK).

### MultiELISA

The murine serum expression levels of eight cytokines, including IL-1β, IL-6, IL-10, IL-17A, IL-33, and TNF-α were measured using a Luminex cytokine assay kit (LXSAMSM-06, R&D Systems, USA) and a Luminex X-200 instrument according to the manufacturer’s protocol (R&D Systems, USA).

### Statistical analysis

The IBM Statistical Package for the Social Sciences (SPSS v26.0, USA) was used for statistical analysis. Missing data were excluded from all statistical analyses. Data are presented as mean ± standard error of the mean (SEM). Unpaired two-tailed Student’s t-test, one-way analysis of variance (ANOVA), and the nonparametric Mann-Whitney U test were used to analyze the datasets. Non-parametric correlation analysis was performed by Spearman’s correlation. Logistic regression was used to predict the anti-TNF response with TMIGD1 mRNA expression before anti-TNF treatment. The RISK cohort dataset was divided into train (60%) and test (40%) sets. The model training was done by a fivefold cross-validation. The significance level was defined as *p*<0.05. All the data are representative of at least three independent experiments.

## Results

### Decreased TMIGD1 is associated with severe inflammatory characteristics of CD

**Fig. 1:**
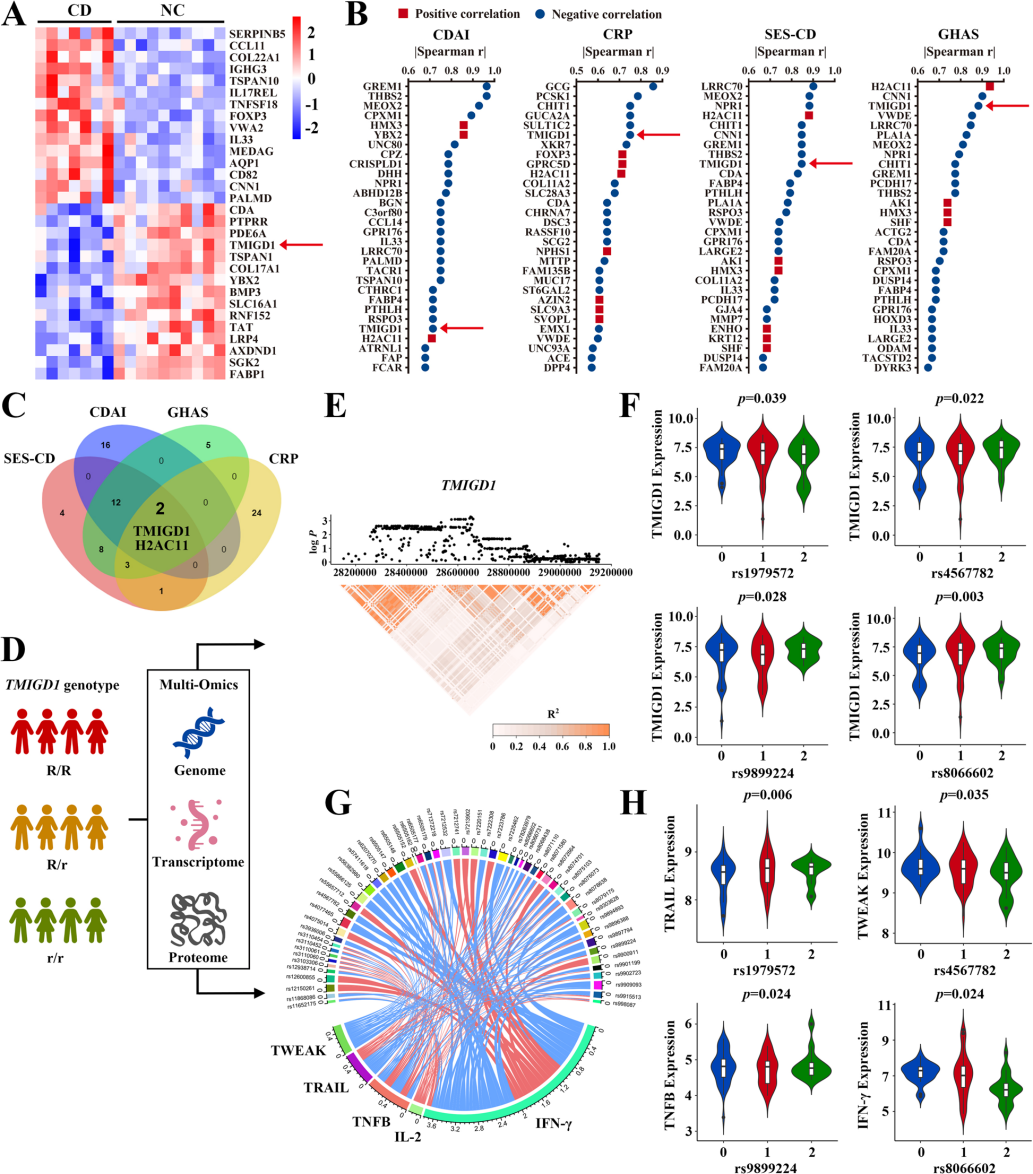
Multi-omics integration indicates that TMIGD1 is negatively correlated with inflammatory characteristics of CD. **A** Heatmap of our in-house dataset showing different expression of mRNA in patients with CD (*n*=7) vs healthy individuals (NC, *n*=10). The arrowhead indicates TMIGD1 expression level. **B** List of top 30 genes correlated with CDAI, CRP, SES-CD, and GHAS; the absolute value of Spearman r using correlation analysis between the FPKM of genes and CDAI, CRP, SES-CD, and GHAS. **C** Venn diagram showing the common genes in the four groups. **D** Multi-omics data integration of the Dutch 1000IBD cohort, including genomic information of 1125 patients, transcriptomic data of intestinal biopsies of 171 patients, and serum Olink proteomic data of 1026 patients. **E** Upper panel indicates the −log_10_ *p* value of *cis*-eQTLs within a region of ±1 Mb around the *TMIGD1* gene center. Lower panel indicates the linkage disequilibrium (LD, *R*^2^) of 831 variants. **F** Examples of significant *cis*-eQTLs. 0, 1, and 2 indicate the homozygotes of reference alleles, heterozygotes, and homozygotes of alternative alleles in patients, respectively. *Y*-axis indicates the normalized gene expression count. **G**, **H** Examples of significant associations between *cis*-eQTL variants and pro-inflammatory proteins in serum. Blue in (**G**) indicates the negative associations while red indicates the positive associations. Values around the circle represent the absolute effect size generated from the linear model. *Y*-axis in (**H**) shows the normalized expression values of serum proteins

Two datasets were included in this study: in-house data from Chinese patients with CD and the Dutch 1000IBD cohort. Analysis of in-house transcriptomic data identified 530 dysregulated genes (417 upregulated and 113 downregulated) in the inflamed colonic mucosa of patients with CD compared to uninflamed mucosa from healthy individuals (|log_2_ fold change|≥1 and *p*<0.05, Fig. 1A). To identify genes most related with overall inflammatory characteristics of CD, we then determined the 30 genes whose expression is most strongly correlated with CDAI, C-reactive protein (CRP), SES-CD, and GHAS (Fig. 1B and Additional file 1: Table S3). Only TMIGD1 and H2AC11 showed a significant correlation with all inflammatory characteristics (Fig. 1C). Since TMIGD1 is mainly expressed in the intestine, we investigated the role of TMIGD1 in the development of CD [13]. We performed multi-omics integration using the 1000IBD cohort dataset, including genomics, transcriptomics of intestinal biopsies, and serum proteomics (Fig. 1D). 278 significant genomic variants nearby *TMIGD1* were observed to potentially exert *cis*-expression quantitative loci (*cis*-eQTL) effects on TMIGD1 expression (Fig. 1E and F, Additional file 2). Moreover, these *cis*-eQTL variants were also associated with numerous pro-inflammatory proteins of CD in serum (e.g., IL-2, TWEAK, and IFN-γ; Fig. 1G and H, Additional file 3). Altogether, patients carrying *TMIGD1* genomic variants showed altered inflammatory characteristics.

**Fig. 2:**
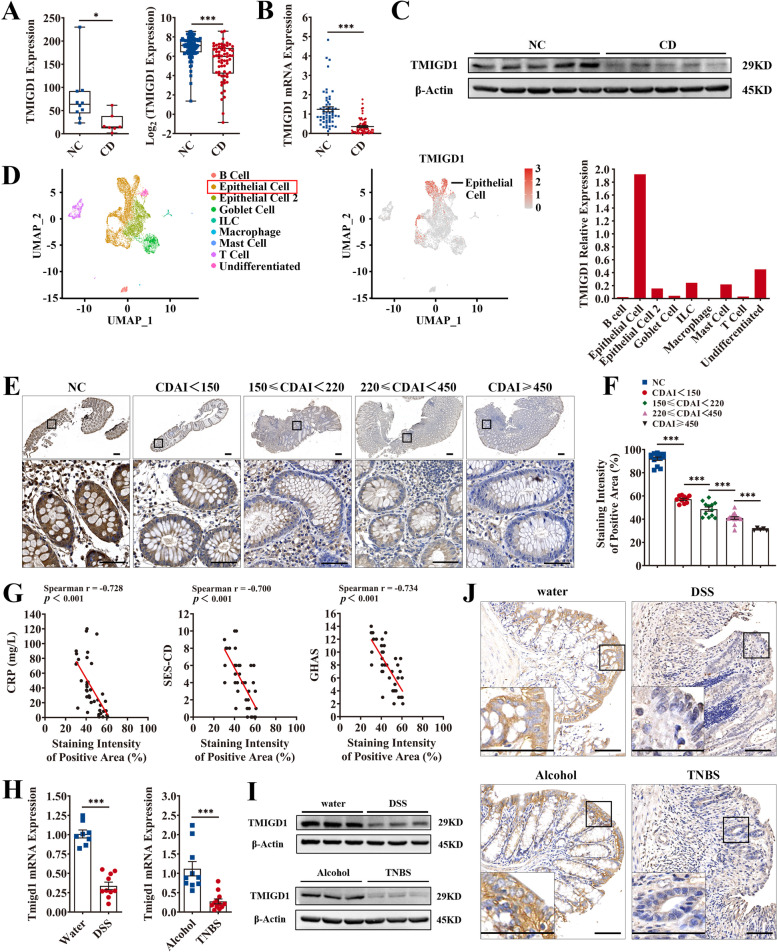
TMIGD1 expression is decreased in the intestinal mucosa of patients with CD and mice with chemically induced colitis. **A** TMIGD1 mRNA expression in intestinal mucosa in the in-house dataset (NC, *n*=10; CD, *n*=7; left) and in the 1000IBD cohort (NC, *n*=107; CD, *n*=64; right). **B** The expression of TMIGD1 mRNA in human intestinal mucosa was assessed via qPCR (NC, *n*=55; CD, *n*=64). **C** The level of TMIGD1 protein in human colonic mucosa. **D** UMAP plot displaying single cells, which are colored as per shared nearest neighboring clusters and cell types based on single-cell sequencing of human colonic biopsy samples (*n*=9) from the GSE116222 dataset (left). The feature plot demonstrates TMIGD1 expression, with each dot representing a single cell (middle). Relative mRNA expression of TMIGD1 in different cell types (right). **E** Representative IHC images of TMIGD1-stained colon sections of NC and different stages of CD. Scale bars, 200 μm (top) and 50 μm (bottom). **F** The staining intensity of TMIGD1-stained colon sections (NC, *n*=13; CDAI<150, *n*=10; 150≤CDAI<220, *n*=12; 220≤CDAI<450, *n*=15; CDAI≥450, *n*=5). The staining intensity of the positive area was quantified using ImageJ software. **G** Spearman’s correlation analysis between the staining intensity of TMIGD1 and CRP, SES-CD, and GHAS (*n*=42). **H** The expression of Tmigd1 mRNA in DSS-induced (water, *n*=8 and DSS, *n*=10) and TNBS-induced (Alcohol, *n*=10; TNBS, *n*=10) colitis was examined via qPCR. **I** The expression of Tmigd1 protein in DSS-induced (up) and TNBS-induced (down) colitis. **J** The expression of Tmigd1 protein in DSS-induced (up) and TNBS-induced (down) colitis was measured via IHC. Scale bars, 100 μm. Data are expressed as mean ± SEM. * *p*<0.05, *** *p*<0.001

We then investigated the phenotype of TMIGD1 expression changes. TMIGD1 was downregulated in the inflamed intestinal mucosa of patients with CD in the in-house dataset (0.294-fold, *p*=0.034) and the 1000IBD cohort (0.339-fold, *p*<0.001; Fig. 2A). Decreased TMIGD1 mRNA was also confirmed in the inflamed intestinal mucosa of patients with CD compared to uninflamed mucosa from healthy individuals (0.284-fold, *p*<0.001, Fig. 2B and Additional file 1: Table S4). TMIGD1 protein expression was also consistently downregulated (Fig. 2C). Analysis of single-cell sequencing data from human colonic biopsy samples (GSE116222) revealed that TMIGD1 was preferentially expressed in epithelial cells (Fig. 2D) [5]. Immunohistochemistry (IHC) staining also showed that TMIGD1 protein was mainly located in the cell membrane and cytoplasm of epithelial cells (Fig. 2E). The staining intensity decreased, whereas CDAI increased (Fig. 2E, F and Additional file 1: Table S5). Moreover, the staining intensity of TMIGD1 was negatively correlated with CRP, SES-CD, and GHAS (Spearman *r*=−0.728 and *p*<0.001 for CRP; Spearman *r*=−0.700 and *p*<0.001 for SES-CD; Spearman *r*=−0.734 and *p*<0.001 for GHAS; Fig. 2G). Hence, downregulation of TMIGD1 was associated with more severe inflammation in patients with CD.

DSS- and TNBS-induced colitis mice models were established. Downregulation of both Tmigd1 mRNA (0.335-fold, *p*<0.001 in DSS-induced colitis; 0.245-fold, *p*<0.001 in TNBS-induced colitis) and protein levels in murine colonic epithelia were detected (Fig. 2H–J). Collectively, downregulation of TMIGD1 may play an important role in the pathogenesis of CD.

### Intestinal-specific *Tmigd1* knockout mice are hypersensitive to chemically induced acute colitis

**Fig. 3:**
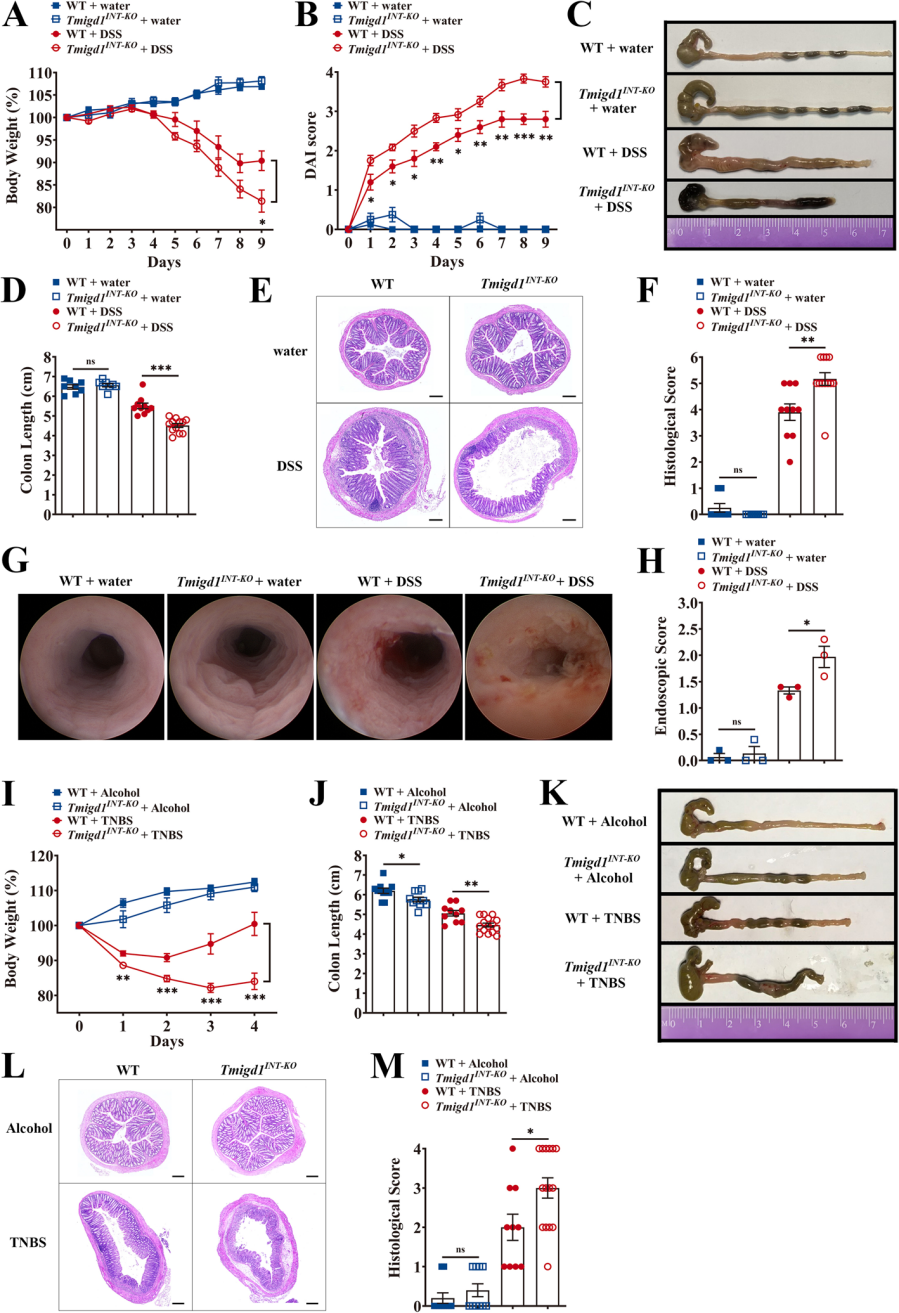
*Tmigd1*^*INT-KO*^ mice are predisposed to chemically induced acute colitis. **A**, **B** Body weight and Disease Activity Index (DAI) score; WT+water (*n*=8), *Tmigd1*^*INT-KO*^+water (*n*=8), WT+DSS (*n*=10), *Tmigd1*^*INT-KO*^+DSS (*n*=12). **C** Representative images of mouse colons. **D** Colon length; WT+water (*n*=8), *Tmigd1*^*INT-KO*^+water (*n*=8), WT+DSS (*n*=10), *Tmigd1*^*INT-KO*^+DSS (*n*=12). **E** Representative colonic histopathological images. Scale bars, 200 μm. **F** Histological scores; WT+water (*n*=8), *Tmigd1*^*INT-KO*^+water (*n*=8), WT+DSS (*n*=10), *Tmigd1*^*INT-KO*^+DSS (*n*=12). **G** Representative colonoscopy images. **H** Endoscopic scores. Every group, *n*=3. **I**, **J** Body weight and colon length; WT+Alcohol (*n*=10), *Tmigd1*^*INT-KO*^+Alcohol (*n*=10), WT+TNBS (*n*=10), *Tmigd1*^*INT-KO*^+TNBS (*n*=15). **K** Representative images of mouse colons. **L** Representative colonic histopathological images. Scale bars, 200 μm. **M** Histological scores; WT+Alcohol (*n*=10), *Tmigd1*^*INT-KO*^+Alcohol (*n*=10), WT+TNBS (*n*=10), *Tmigd1*^*INT-KO*^+TNBS (*n*=15). Data are expressed as mean ± SEM. ns, no significance, * *p*<0.05, ** *p*<0.01, *** *p*<0.001

To investigate the role of TMIGD1 in CD development, *Tmigd1*^*INT-KO*^ and *Tmigd1*-floxed without Villin-Cre (WT) mice were generated (Additional file 1: Fig. S1). *Tmigd1*^*INT-KO*^ mice with DSS-induced colitis showed more body weight loss, more severe bloody diarrhea, higher disease activity, and shorter colons than DSS-treated WT mice (Fig. 3A–D, Additional file 1: Fig. S2A). More severe histological damage, such as colonic epithelial damage and lamina propria lymphocyte infiltration, was also observed in DSS-treated *Tmigd1*^*INT-KO*^ mice (Fig. 3E, F). In addition, DSS-treated *Tmigd1*^*INT-KO*^ mice showed more severe endoscopic damage, such as bleeding mucosa, altered vascular pattern, and ulcers (Fig. 3G, H). Next, we examined whether *Tmigd1*^*INT-KO*^ mice were more susceptible to TNBS-induced acute colitis. When compared with WT mice, TNBS-treated *Tmigd1*^*INT-KO*^ mice lost more body weight and exhibited more significant colon shortening (Fig. 3I–K, Additional file 1: Fig. S2B). Histological analysis also showed more severe tissue damage with distortion of gland architecture, tissue edema, and increased inflammatory cell infiltration in TNBS-treated *Tmigd1*^*INT-KO*^ mice (Fig. 3L and M).

### TMIGD1 downregulation aggravates inflammation and impairs intestinal epithelial barrier integrity

**Fig. 4:**
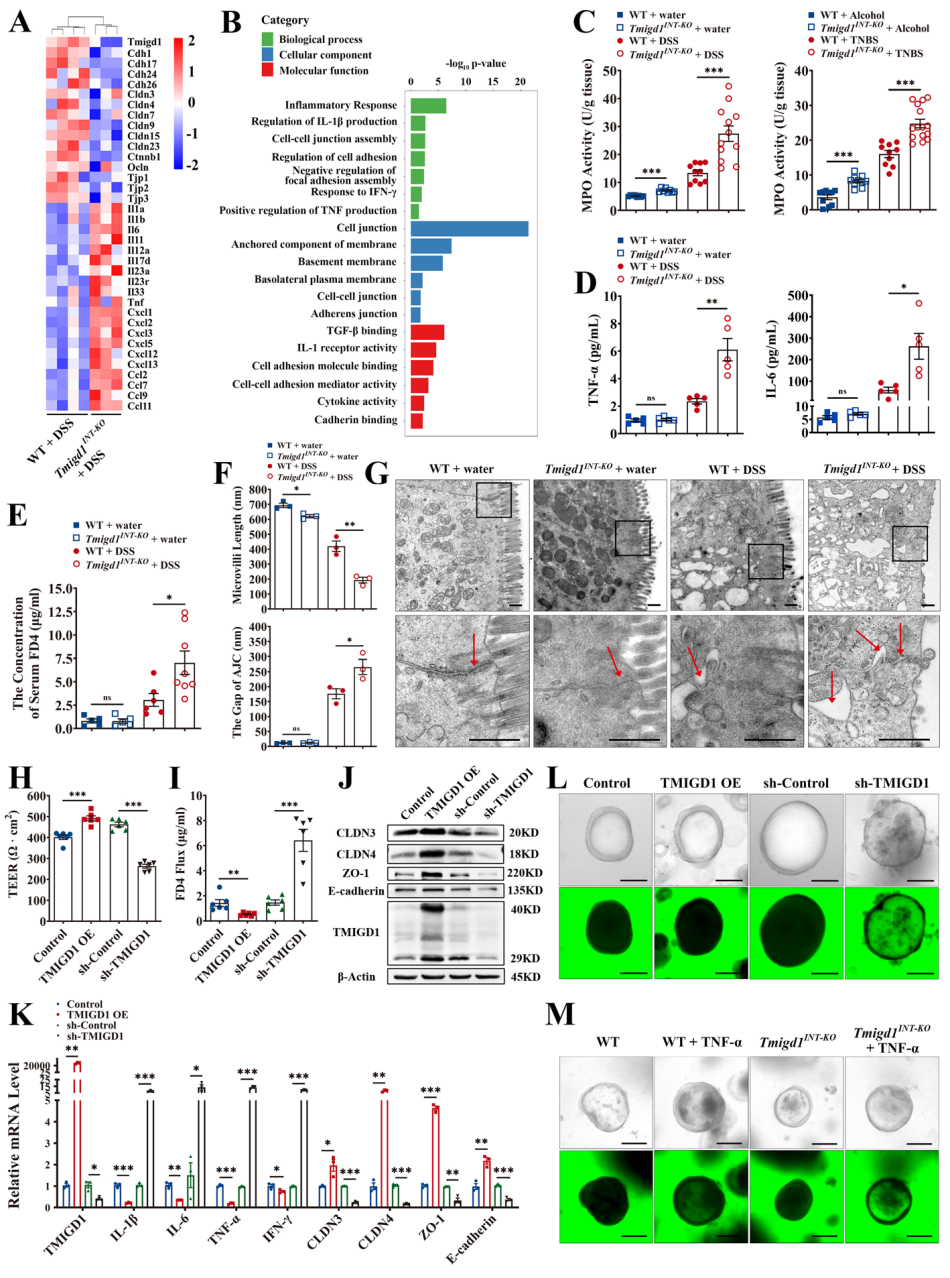
TMIGD1 downregulation aggravates inflammation and weakens intestinal epithelial barrier function in the inflammatory environment. **A** Transcriptomic analysis of genes related to epithelial barrier function and inflammatory cytokines in the colonic tissues of WT+DSS and *Tmigd1*^*INT-KO*^+DSS mice. **B** The differentiated genes were enriched in barrier function and inflammation-relevant cytokines according to gene ontology (GO) analysis. **C** Myeloperoxidase (MPO) activity in colon tissues; WT+water (*n*=8), *Tmigd1*^*INT-KO*^+water (*n*=8), WT+DSS (*n*=10), *Tmigd1*^*INT-KO*^+DSS (*n*=12); WT+Alcohol (*n*=10), *Tmigd1*^*INT-KO*^+Alcohol (*n*=10), WT+TNBS (*n*=10), *Tmigd1*^*INT-KO*^+TNBS (*n*=15). **D** Serum TNF-α and IL-6 concentrations were tested using multiELISA; Every group, *n*=5. **E** The concentration of serum FD4; WT+water (*n*=5), *Tmigd1*^*INT-KO*^+water (*n*=5), WT+DSS (*n*=6), *Tmigd1*^*INT-KO*^+DSS (*n*=8). **F** Measurement of microvilli length and AJC gaps in colonic epithelial cells using TEM. Every group, *n*=3. **G** Representative TEM images of the colonic mucosa. The arrowheads indicate AJC. Scale bars, 500 nm. **H**, **I** TEER and FD4 permeability of Caco2 monolayer cell model after TNF-α stimulation. **J**, **K** The expression of AJC proteins (**J**) and inflammation-relevant cytokine mRNA and AJC mRNA (**K**) in NCM460 cells after TNF-α stimulation. **L** After TNF-α treatment, FD4 permeability of human colonic organoids was detected using confocal microscopy. Scale bars, 100 μm. **M** The FD4 permeability of colonic organoids from *Tmigd1*^*INT-KO*^ and WT mice was detected using confocal microscopy before and after TNF-α treatment. Scale bars, 100 μm. Data are expressed as mean ± SEM. ns, no significance, * *p*<0.05, ** *p*<0.01, *** *p*<0.001

To explore the biological function of dysregulated TMIGD1, transcriptomic analysis of colonic tissues from DSS-treated *Tmigd1*^*INT-KO*^ mice and DSS-treated WT littermates was conducted. Increased expression of pro-inflammatory cytokines (e.g., Ils, Cxcls, and Ccls) and reduced expression of epithelial barrier markers (e.g., Cdhs, Cldns, and Tjps) were observed in DSS-treated *Tmigd1*^*INT-KO*^ mice (Fig. 4A). Gene Ontology (GO) analysis revealed enrichment for cell-cell junctions, inflammatory responses, and TNF production (Fig. 4B). We further evaluated markers of inflammation *in vivo*. Both neutrophils and CD4+ T cells were upregulated in the colonic tissue of *Tmigd1*^*INT-KO*^ mice with acute colitis (Fig. 4C, Additional file 1: Fig. S2C-S2D). In addition, pro-inflammatory cytokines were remarkably upregulated in the DSS-treated and TNBS-treated *Tmigd1*^*INT-KO*^ mice (Fig. 4D, Additional file 1: Fig. S2E-S2F). As for mucosal barrier function, DSS- and TNBS-treated *Tmigd1*^*INT-KO*^ mice exhibited higher transepithelial permeability of FD4 (Fig. 4E and Additional file 1: Fig. S3A). The colon tissue of *Tmigd1*^*INT-KO*^ mice with chemically induced colitis showed decreased tight junction and adherence junction complex proteins (Additional file 1: Fig. S3B-S3G). In addition, TEM revealed shorter and fewer microvilli, more damaged intracellular junction complexes, and increased paracellular space with saccular dilatation in DSS- and TNBS-treated *Tmigd1*^*INT-KO*^ mice (Fig. 4F, G and Additional file 1: Fig. S3H-S3I).

The biological function of TMIGD1 was also confirmed *in vitro*. First, to determine which inflammatory factor contributed to the decreased TMIGD1 expression, we treated NCM460 and Caco2 cell lines with CD-relevant cytokines for 48 h. We found that only TNF-α significantly downregulated TMIGD1 expression in both cell lines (Additional file 1: Fig. S4A). Moreover, TNF-α induced decreased expression of TMIGD1 in a dose-dependent manner (Additional file 1: Fig. S4B). TNF-α (20 ng/mL) treatment for 48 h was considered effective and was used for subsequent *in vitro* experiments. Second, we measured TEER and paracellular FD4 permeability in Caco2 monolayer cell model to evaluate the tightness of junctions between adjacent colonic cells and the intestinal epithelial barrier function. Compared to the control group, the TMIGD1-overexpressing cells exhibited increased TEER and reduced FD4 permeability after TNF-α treatment, whereas knockdown of TMIGD1 led to decreased TEER and increased FD4 permeability (Fig. 4H, I). The expression of AJC components also confirmed that TMIGD1 protected intestinal epithelial barrier function after TNF-α treatment, as evidenced by the upregulation of CLDN3, CLDN4, ZO-1, and E-cadherin in TMIGD1-overexpressed cells (Fig. 4J, K, Additional file 1: Fig. S5A-S5B). Third, pro-inflammatory cytokines (e.g., IL-1β, IL-6, and TNF-α) were downregulated in TMIGD1-overexpressing cell lines and upregulated in TMIGD1 knockdown cell lines, further indicating that TMIGD1 inhibited inflammation (Fig. 4K, Additional file 1: Fig. S5B).

Finally, we generated TMIGD1-overexpressed and TMIGD1-knockdown human colonic organoids using a lentiviral system (Additional file 1: Fig. S5C-S5D). After TNF-α treatment for 48 h, FD4 was added to the culture medium, and barrier function was assessed. FD4 was exclusively observed in TMIGD1-knockdown organoids under confocal microscopy, indicating that TMIGD1 helped to maintain intact epithelial integrity (Fig. 4L). Similarly, colonic organoids from *Tmigd1*^*INT-KO*^ and WT mice were generated (Additional file 1: Fig. S5E-S5F). After TNF-α treatment and FD4 supplementation, *Tmigd1*^*INT-KO*^ mice organoids showed more intense intraluminal FD4 signals than those generated from WT mice (Fig. 4M).

### TMIGD1 directly interacts with cytoplasmic BANF1 and modulates downstream NF-κB pathway

**Fig. 5:**
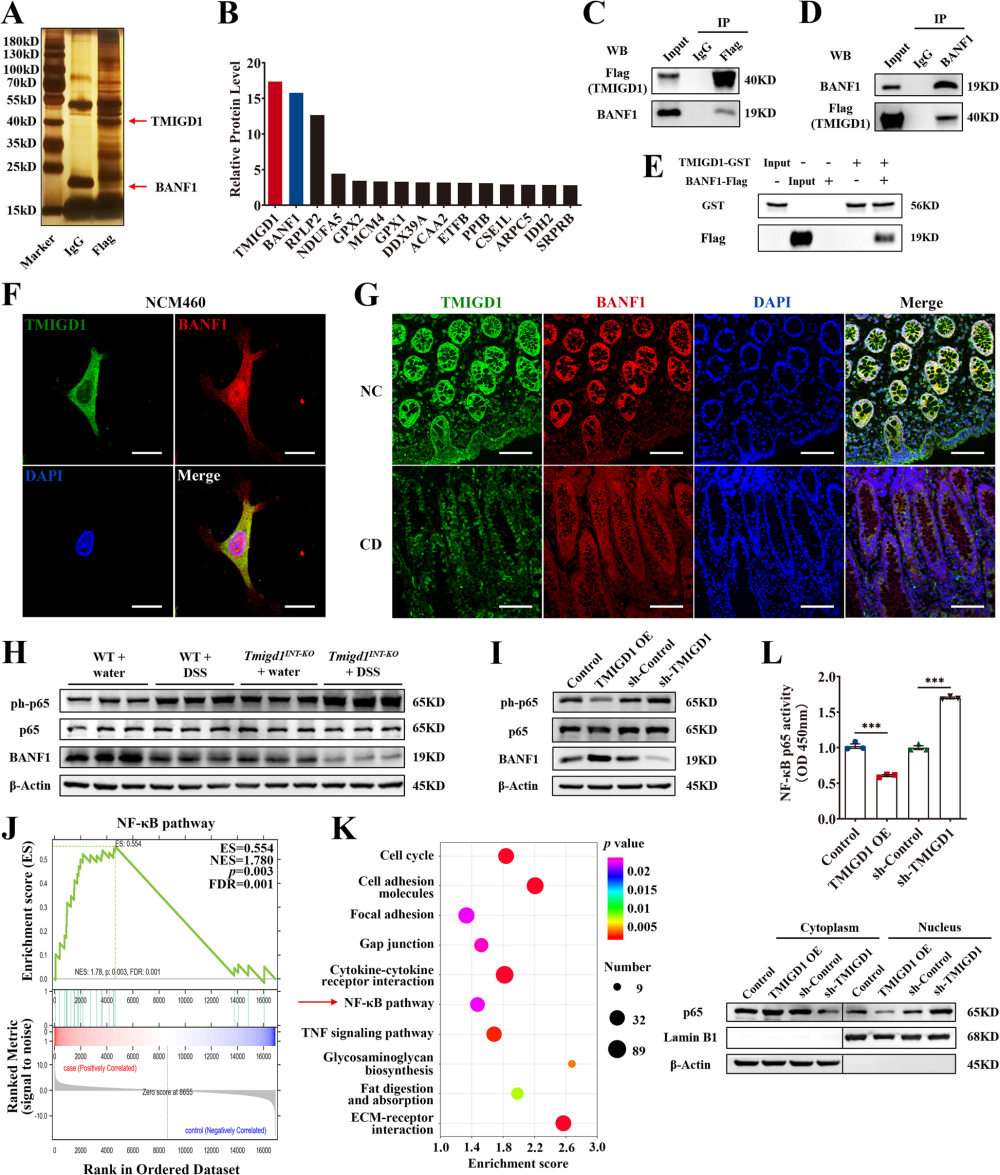
TMIGD1 directly interacts with BANF1 and modulates the NF-κB pathway. **A** Silver stain identified the TMIGD1-protein complex immunoprecipitated by anti-IgG or anti-FLAG antibody from the lysates of NCM460 cells transfected with lentivirus overexpressing FLAG-tagged TMIGD1. The arrowheads indicate bands of TMIGD1 and BANF1. **B** Proteomics analysis showed the 15 most enriched proteins from the immunoprecipitate of anti-FLAG antibody. **C**, **D** Immunoprecipitate by anti-FLAG antibody (**C**) or anti-BANF1 antibody (**D**) from the lysates of NCM460 cells transfected with lentivirus overexpressing FLAG-tagged TMIGD1. IP lysate (10%) was used as input. **E** GST pull-down assays of purified recombinant TMIGD1-GST and BANF1-Flag proteins. **F**, **G** Confocal microscope showed colocalization of TMIGD1 and BANF1 in NCM460 cells (**F**) and human epithelial mucosa (**G**). Scale bars, 20 μm for NCM460 cells and 100 μm for human epithelial mucosa. **H** Protein levels of BANF1, p65, and phosphorylated p65. **I** Protein levels of BANF1, p65, and phosphorylated p65 after TNF-α stimulation in NCM460 cells. **J** GSEA revealed significant enrichment of the NF-κB pathway. **K** Genes relevant to the NF-κB pathway were enriched in the comparison of WT+DSS and *Tmigd1*^*INT-KO*^+DSS groups according to KEGG analysis. **L** After TNF-α stimulation, the p65 protein level in cytoplasmic and nuclear fractions (down) and results of transcription factor binding assay of nuclear p65 (up) of NCM460 cells. Data are expressed as mean ± SEM. *** *p*<0.001

To elucidate the potential molecular mechanisms of TMIGD1, we generated NCM460 and Caco2 cell lines overexpressing FLAG-tagged TMIGD1 (Additional file 1: Fig. S6A). Immunoaffinity purification (IP), high-throughput mass spectrometry (LC-MS/MS), and proteomics analysis were performed to identify potential TMIGD1 protein interaction partners (Fig. 5A, Additional file 1: Fig. S6B). Among the several potential target proteins, BANF1 was enriched in both NCM460 (15.8-fold) and Caco2 (2.7-fold) cell lines and was identified as a prime candidate (Fig. 5B, Additional file 1: Fig. S6C-S6E). The direct interaction between TMIGD1 and BANF1 was further confirmed by co-immunoprecipitation (co-IP) and GST pull-down assays (Fig. 5C–E). The colocalization of TMIGD1 and BANF1 in the cytoplasm also supported their direct interaction (Fig. 5F). Both TMIGD1 and BANF1 were decreased in the colonic epithelium of patients with CD compared to the expression in healthy individuals (Fig. 5G). Moreover, a significant reduction in BANF1 protein level was observed in *Tmigd1*^*INT-KO*^ mice compared to WT mice treated with either DSS or TNBS (Fig. 5H, Additional file 1: Fig. S7A). BANF1 levels increased after ectopic expression of TMIGD1, whereas a decrease in BANF1 was detected after TMIGD1 knockdown in cell lines (Fig. 5I, Additional file 1: Fig. S7B). Thus, the TMIGD1-BANF1 interaction may act synergistically on downstream pathways.

We analyzed the transcriptomic data obtained from the colonic tissues of *Tmigd1*^*INT-KO*^ and WT mice with DSS-induced colitis. Gene Set Enrichment Analysis (GSEA) and Kyoto Encyclopedia of Genes and Genomes (KEGG) pathway enrichment analysis revealed that the NF-κB pathway was significantly enriched in DSS-treated *Tmigd1*^*INT-KO*^ mice (Fig. 5J, K). As expected, the phosphorylation of p65 was detected in DSS- and TNBS-induced colitis models, and p65 phosphorylation increased significantly in *Tmigd1*^*INT-KO*^ mice compared to WT mice (Fig. 5H, Additional file 1: Fig. S7A). Furthermore, TMIGD1 overexpression resulted in lower p65 phosphorylation in whole-cell extracts and decreased p65 in the nucleus, with attenuated DNA-binding activity of nuclear p65. In contrast, TMIGD1 knockdown enhanced the p65 phosphorylation in whole-cell extracts, increased the accumulation of p65 in the nucleus, and activated the DNA-binding activity of nuclear p65 (Fig. 5I, L, Additional file 1: Fig. S7B-S7C). However, other markers of the NF-κB pathway (e.g., IKKα, IKKβ, IκBα, and their phosphorylation) were unchanged (Additional file 1: Fig. S7D). Therefore, our data suggest that TMIGD1 directly binds to cytoplasmic BANF1 and inhibits the NF-κB pathway.

### BANF1 maintains the intestinal epithelial barrier and attenuates inflammation via the NF-κB pathway

**Fig. 6:**
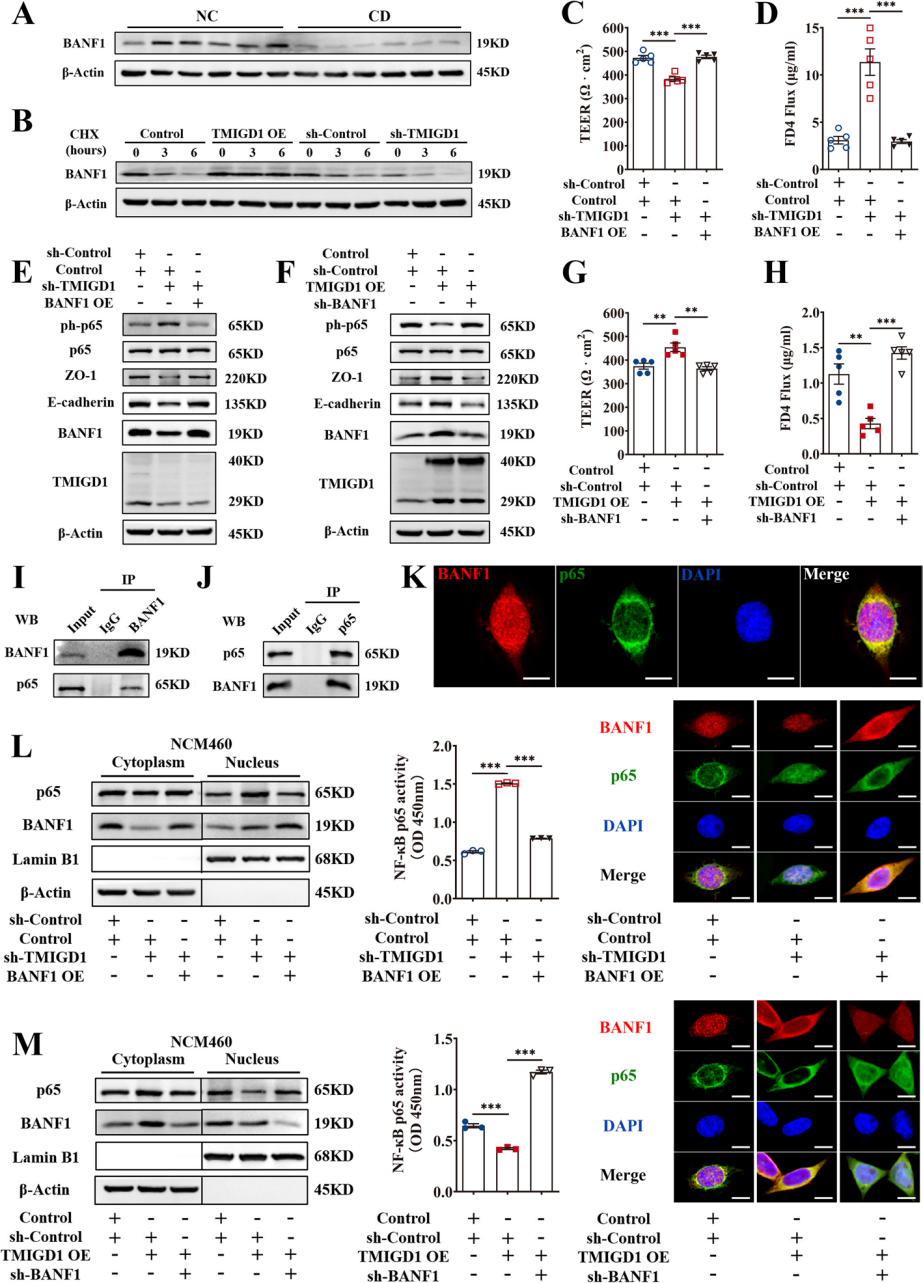
BANF1 is crucial for TMIGD1 to maintain the intestinal epithelial barrier and attenuate inflammation via the NF-κB pathway. **A** The expression level of BANF1 protein in the colonic tissues of patients with CD and healthy individuals. **B** Degradation of BANF1 protein in NCM460 cells treated with 50 μg/mL CHX was evaluated. **C**, **D** TEER and FD4 permeability were measured after TNF-α stimulation in Caco2 monolayer model. **E**, **F** The expression levels of AJC proteins, p65, and phosphorylated p65 after TNF-α stimulation in NCM460 cells. **G**, **H** TEER and FD4 permeability were measured after TNF-α stimulation in Caco2 monolayer model. **I**, **J** Immunoprecipitate by anti-BANF1 antibody (**I**) and anti-p65 antibody (**J**) from the lysates of NCM460 cells. IP lysate (10%) was used as input. **K** Confocal microscope showing colocalization of BANF1 and p65 in NCM460 cells. Scale bars, 10 μm. **L**, **M** After TNF-α stimulation, expression of p65 and BANF1 in cytoplasmic and nuclear fractions (left), transcription factor binding assay of nuclear p65 (middle), and subcellular localization of p65 (right) in NCM460 cells. Scale bars, 10 μm. Data are expressed as mean ± SEM. ** *p*<0.01, *** *p*<0.001

BANF1 protein was reduced in the inflamed colonic mucosa of patients with CD, DSS-, and TNBS-induced colitis compared with the corresponding control groups (Figs. 5G, H and 6A and Additional file 1: Fig. S7A). To explore the reason for synchronized protein expression between TMIGD1 and BANF1, we used 50 μg/mL cycloheximide to inhibit protein synthesis in NCM460 cells and observed changes in the level of BANF1 protein over time. TMIGD1 overexpression maintained BANF1 protein levels by inhibiting BANF1 degradation, while TMIGD1 knockdown led to rapid degradation of BANF1. Therefore, the TMIGD1-BANF1 interaction may help maintain the expression of BANF1 and protect BANF1 from degradation (Fig. 6B).

Moreover, while knockdown of TMIGD1 led to decreased TEER and increased FD4 permeability in the Caco2 monolayer cell model, overexpression of BANF1 in the TMIGD1-knockdown cells restored TEER and reduced FD4 permeability (Fig. 6C, D). BANF1 overexpression also significantly reversed the downregulated expression levels of AJCs and the increased expression of pro-inflammatory cytokines when compared to the TMIGD1 knockdown group (Fig. 6E, Additional file 1: Fig. S8A, S8C, and S8D). In contrast, after inhibition of BANF1, TMIGD1-overexpressed cells showed weakened intestinal epithelial barrier function, increased pro-inflammatory cytokines, and decreased AJCs (Fig. 6F–H, Additional file 1: Fig. S8B-S8D). Hence, BANF1 is a crucial mediator for TMIGD1 in maintaining the intestinal epithelial barrier and attenuating inflammation.

The protein-protein interaction between BANF1 and p65, the main effector of the NF-κB pathway, was verified by co-IP (Fig. 6I, J). This was also supported by the colocalization of BANF1 and p65 (Fig. 6K). After knocking down TMIGD1, cytoplasmic BANF1 levels decreased significantly. However, BANF1 overexpression caused decreased p65 phosphorylation in whole-cell extracts, downregulated nuclear p65 expression, and attenuated nuclear p65 DNA-binding activity (Fig. 6E and L, Additional file 1: Fig. S8A and S8E). In contrast, TMIGD1 overexpression upregulated cytoplasmic BANF1. P65 phosphorylation in whole-cell extracts, nuclear p65 expression, and the DNA-binding activity of nuclear p65 were upregulated after knocking down BANF1 (Fig. 6F and M, Additional file 1: Fig. S8B and S8F). Altogether, TMIGD1 maintained protein expression of BANF1. Cytoplasmic BANF1 further captured p65, inhibited the translocation of p65 from cytoplasm to nucleus, and finally decreased phosphorylation of p65 (S536). Consequently, BANF1 is crucial for TMIGD1 function by inhibiting the NF-κB pathway.

### Introducing TMIGD1 and BANF1 alleviates inflammation and restores intestinal barrier function

**Fig. 7:**
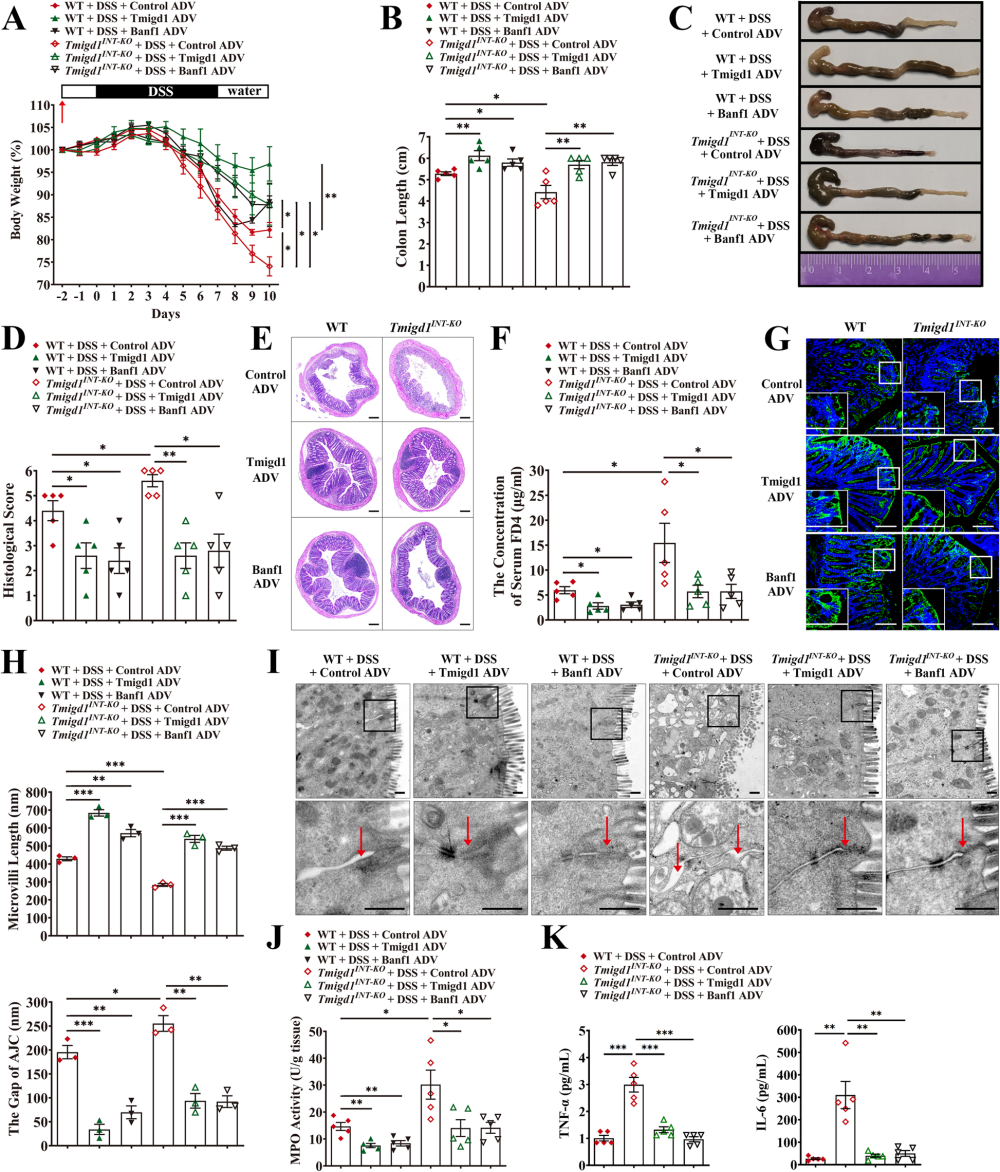
Restoring TMIGD1 and BANF1 alleviates inflammation and resumes intestinal barrier function. **A**, **B** Body weight and colon length. The arrowhead indicates the time point at which ADV was intraperitoneally injected to boost TMIGD1 and BANF1 expression in mice. Every group, *n*=5. **C** Representative images of colons. **D** Histological scores. Every group, *n*=5. **E** Representative colonic HE images. Scale bars, 200 μm. **F** The concentration of serum FD4. Every group, *n*=5. **G** Representative images of ZO-1-stained colon sections. Scale bars, 100 μm. **H** Measurement of microvilli length (up) and AJC gaps (down) in colonic epithelial cells using TEM. Every group, *n*=3. **I** Representative TEM images of the colonic mucosa. The arrowheads indicate AJC. Scale bars, 500 nm. **J** MPO activity in colon tissues. Every group, *n*=5. **K** Serum TNF-α and IL-6 concentrations were detected using multiELISA; Every group, *n*=5. Data are expressed as mean ± SEM. * *p*<0.05, ** *p*<0.01, *** *p*<0.001

As a reduction in the TMIGD1-BANF1 interaction led to intestinal barrier dysfunction and inflammation via activation of the NF-κB pathway, we further explored whether restoring TMIGD1 and BANF1 levels could rescue inflammation and mucosal barrier function. We intraperitoneally injected adenovirus (ADV) to boost TMIGD1 and BANF1 expression in mice 2 days before inducing colitis with DSS. After restoring TMIGD1 and BANF1, both WT and *Tmigd1*^*INT-KO*^ mice with colitis exhibited increased body weight and colon length, less bloody diarrhea, and lower disease activity (Fig. 7A–C, Additional file 1: Fig. S9A-S9C). They also showed histological recovery with more normal-appearing epithelium and less lymphocyte infiltration (Fig. 7D, E). In addition, TMIGD1 and BANF1 re-expression rescued intestinal barrier function, resulting in lower serum FD4 flux accompanied by enhanced expression of AJCs (Fig. 7F, G, Additional file 1: Fig. S9D-S9E). In the ultrastructure, the presence of intact intracellular junction complexes and well-preserved microvilli without serious distortion confirmed the therapeutic effects of TMIGD1 and BANF1 supplementation (Fig. 7H, I). Restoring TMIGD1 and BANF1 also inhibited the NF-κB pathway, reduced CD4+ T cell and neutrophil accumulation, and attenuated pro-inflammatory cytokine excretion, especially for NF-κB pathway-related cytokines (Fig. 7J, K, Additional file 1: Fig. S9F-S9H).

### TMIGD1 expression predicts response to anti-TNF treatment

**Fig. 8:**
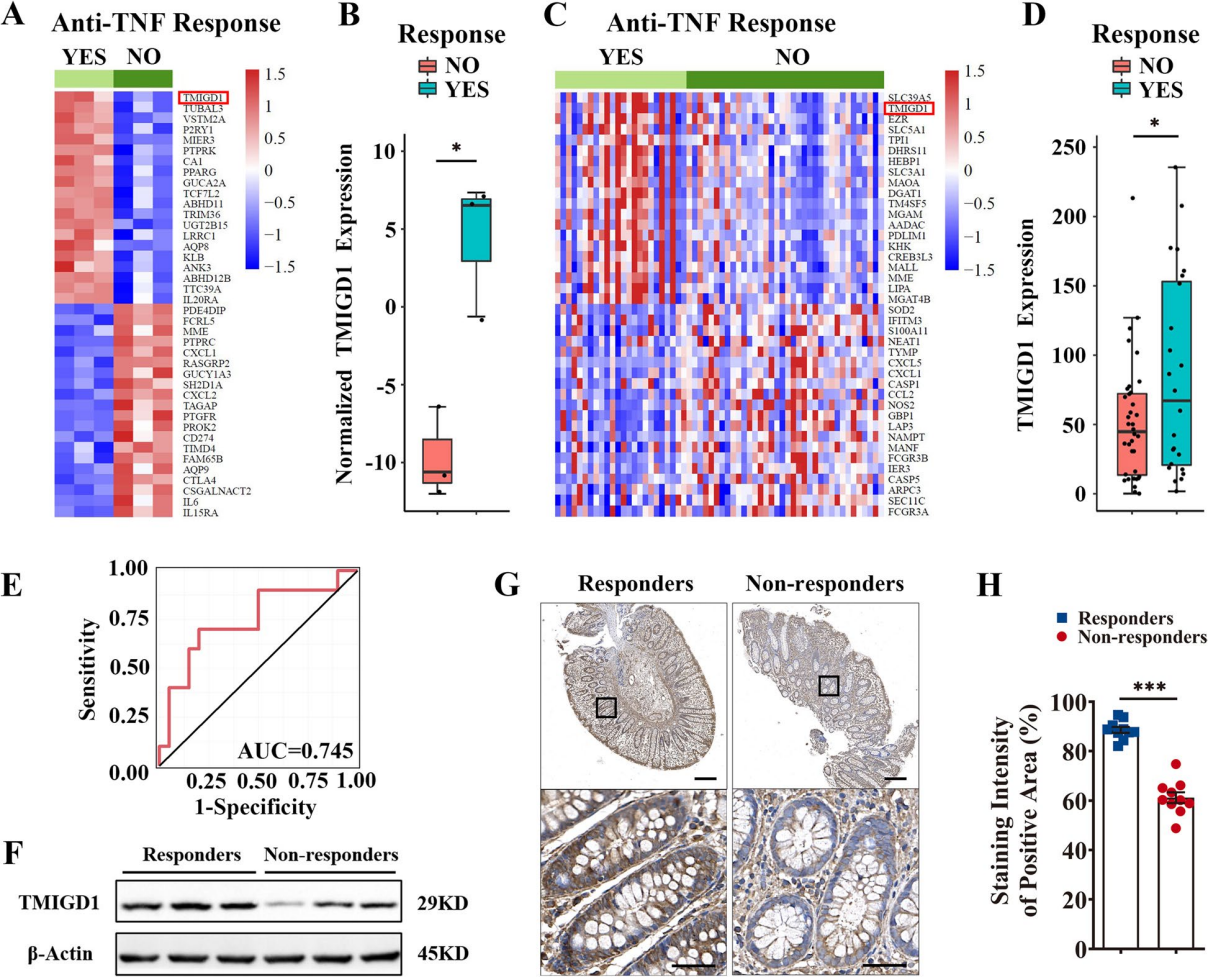
TMIGD1 expression predicts response to anti-TNF treatment. **A**, **B** Heatmap and box plot from GSE111761 showing different baseline expressions of mRNA in LPMCs of 3 anti-TNF responding patients with CD and 3 non-responders. **C**, **D** Heatmap and box plot of the GSE134881 dataset showing different baseline expressions of mRNA in intestinal tissues of anti-TNF responding (*n*=24) and non-responding (*n*=36) patients with CD. **E** Receiver operating characteristic (ROC) curve for the prediction of anti-TNF response with baseline TMIGD1 mRNA expression (AUC=0.745, 95% CI: 0.540–0.950). **F** The expression of TMIGD1 protein in colonic tissues before anti-TNF treatment. **G** Representative IHC images of TMIGD1-stained sections from responders and non-responders before anti-TNF treatment. Scale bars, 200 μm (top) and 50 μm (bottom). **H** The staining intensity of TMIGD1-stained sections from responders (*n*=10) and non-responders (*n*=10). The staining intensity of the positive area was quantified using the ImageJ software. Data are expressed as mean ± SEM. * *p*<0.05, *** *p*<0.001

TMIGD1 was downregulated by TNF-α, and decreased TMIGD1 enhanced TNF-α excretion in turn, suggesting a close relationship between TMIGD1 and TNF-α. Therefore, we tested whether TMIGD1 expression was associated with responsiveness to anti-TNF therapy in patients with CD. Preliminary evidence in support of this theory was obtained by analysis on two published transcriptome datasets: lamina propria mononuclear cells (LPMCs) of anti-TNF responders and non-responders (GSE111761) [27], and intestinal tissues from the RISK cohort (GSE134881) [28]. We found that baseline TMIGD1 was significantly elevated in treatment-naïve anti-TNF responders in both datasets (Fig. 8A–D). In the RISK cohort, baseline TMIGD1 expression could predict anti-TNF response with 77.2% sensitivity and 62.5% specificity using a logistic model (AUC=0.745; 95% CI, 0.540–0.950; Fig. 8E). Moreover, we retrospectively included biopsies from anti-TNF responders and non-responders with similar disease activity before anti-TNF therapy (Additional file 1: Table S6). IHC and western blotting analyses revealed TMIGD1 downregulation in the inflamed colonic mucosa of non-responders compared to responders, before anti-TNF treatment (Fig. 8F–H). In summary, baseline TMIGD1 was a potential biomarker for predicting response to anti-TNF treatment.

## Discussion

In the present study, we demonstrated that TMIGD1 played a protective role in CD development (Additional file 1: Fig. S10). Analysis of multi-omics integration demonstrated TMIGD1 was negatively associated with inflammatory characteristics of CD. TMIGD1 expression was markedly decreased in inflamed colonic mucosa of patients with CD and in mouse colitis. *Tmigd1*^*INT-KO*^ mice exhibited enhanced susceptibility to DSS-induced and TNBS-induced colitis. Furthermore, TMIGD1 inhibited inflammation and protected intestinal barrier function in CD. In physiological condition, TMIGD1 directly interacted with cytoplasmic BANF1 and maintained protein expression of BANF1. Cytoplasmic BANF1 further captured p65, inhibited p65 translocation from cytoplasm to nucleus, and finally suppressed the activation of the NF-κB pathway. However, in an inflammatory environment, the transcription of TMIGD1 was repressed, and reduced TMIGD1 failed to play the protective role aforementioned above, therefore aggravating inflammation and mucosal barrier damage. Finally, restoring TMIGD1 helped to maintain intestinal mucosal homeostasis. The higher expression of TMIGD1 predicted responsiveness to anti-TNF treatment in patients with CD. In conclusion, TMIGD1 is a therapeutic target for CD and potentially guides the optimization of anti-TNF therapy.

During the pathogenesis of CD, intestinal barrier breakdown leads to increased intestinal permeability, invasion of pathogens and toxic substances, excessive immune responses, and chronic inflammatory responses [29, 30]. With a molecular structure similar to that of the classic junctional adhesion molecule subfamily, TMIGD1 is likely to play a role similar to that of other AJC proteins in intestinal barrier integrity [8]. In our study, we identified the anti-inflammatory role of TMIGD1 based on the population-scale multi-omics. The expression of TMIGD1 was negatively associated with clinical indices of patients with CD, such as CDAI, serum CRP, SES-CD, and GHAS score. Moreover, CD patients carrying TMIGD1 genomic variants showed higher levels of pro-inflammatory proteins. In the inflammatory environment, transcription of TMIGD1 was suppressed. Decreased TMIGD1 in turn led to severe barrier damage and enhanced inflammation, thus forming a vicious cycle. In contrast, restoring TMIGD1 levels alleviated inflammation and recovered barrier function and intestinal integrity.

Previous studies showed that BANF1 was a highly conserved, ubiquitous, and self-associating protein (9KD) and functioned as a dimer (19KD) [31]. BANF1 has dynamic subcellular distribution in the cytoplasm, nucleoplasm, and nucleus [32]. BANF1 has numerous functions due to its ability to bind both DNA and proteins. Most importantly, BANF1 protects genome integrity and ensures successful completion of mitosis [33]. BANF1 also performs anti-inflammatory function. In psoriasis, translocation of BANF1 from the cytoplasm to the nucleus suppresses the phosphorylation of c-Jun and mitigates cutaneous inflammation [34]. In our study, we reveal a new role of BANF1 and demonstrated it as a link-bridge in the TMIGD1-BANF1-p65 interaction in intestinal epithelial cells. In particular, TMIGD1 directly binds to cytoplasmic BANF1, maintaining BANF1 levels in intestinal epithelial cells. Subsequently, BANF1 captures cytoplasmic p65, inhibiting the translocation of p65 from cytoplasm to nucleus and thus decreasing phosphorylation of p65 (S536). In turn, decreased p65 phosphorylation reduces the production of NF-κB pathway-relevant cytokines and maintains barrier integrity by promoting AJC protein production. Furthermore, we found that restoring BANF1 was essential for TMIGD1 to maintain intestinal homeostasis *in vitro* and *in vivo*. However, further research is required to identify the exact binding motifs between TMIGD1, BANF1, and p65.

Currently, anti-TNF therapy is ineffective in a large proportion of patients with CD [35]. Biomarkers for treatment response are important for personalized medicine, which optimizes efficacy, decreases the risk of adverse events, and reduces the costs for a selected patient [36]. However, most of the predictive factors remain controversial in clinical practice. Our study revealed that downregulation of TMIGD1 contributed to the positive feedback loop of TNF-α-induced inflammatory injury. TMIGD1 expression was reduced by TNF-α and this downregulation led to uncontrolled TNF-α via loss of regulation of the NF-κB pathway. Our finding showed that TMIGD1 was a promising biomarker because decreased baseline TMIGD1 levels in the intestinal mucosa or LPMCs were associated with non-responsiveness to anti-TNF therapy (e.g., infliximab) in patients with CD. However, similar investigations should be replicated with a larger sample size in the future. Further research is also required to unveil the mechanisms underlying this predictive relationship.

## Conclusions

In summary, TMIGD1 expression is negatively associated with inflammatory characteristics of CD. TMIGD1 inhibits inflammation and maintains intestinal barrier function by directly interacting with cytoplasmic BANF1 and subsequent inactivation of the NF-κB pathway. Our study proposes that TMIGD1 is a promising therapeutic target for CD and may guide the optimization of anti-TNF therapy.

### Supplementary Information

**Additional file 1:** **Table S1.** Antibodies. **Table S2.** Primers used in qPCR. **Table S3.** Clinical characteristics of patients with CD and healthy individuals in transcriptome sequencing. **Table S4.** Clinical characteristics of patients with CD and healthy individuals analyzed for TMIGD1 expression. Table S5. Clinical characteristics of patients with CD and healthy individuals analyzed for IHC staining of TMIGD1. **Table S6.** Clinical characteristics of anti-TNF responding and non-responding patients with CD before anti-TNF treatment. **Fig. S1.** Verification of *Tmigd1*^INT-KO^ mice. **Fig. S2.** Chemically induced colitis in *Tmigd1*^INT-KO^ mice show more severe inflammation. **Fig. S3.** Chemically induced colitis in *Tmigd1*^INT-KO^ mice show more severe barrier dysfunction. **Fig. S4.** TMIGD1 is downregulated after TNF-α stimulation. **Fig. S5.** TMIGD1 modulates barrier function and inflammation. **Fig. S6.** TMIGD1 binds to BANF1. **Fig. S7.** TMIGD1 modulates BANF1 and inactivates NF-κB pathway. **Fig. S8.** BANF1 is crucial for TMIGD1 to maintain barrier function and inhibit inflammation. **Fig. S9.** Restoring TMIGD1 and BANF1 repairs barrier function and attenuates inflammation. **Fig. S10.** The proposed model for the landscape of TMIGD1-BANF1-NF-κB pathway in CD.

**Additional file 2.** The cis-eQTL effects of genomic variants nearby TMIGD1 on TMIGD1 expression in the 1000IBD cohort dataset.

**Additional file 3.** The association between cis-eQTL variants and pro-inflammatory proteins of CD in serum in the 1000IBD cohort dataset.

**Additional file 4.** The uncropped original gels or western blots from the figures.

## Data Availability

All data associated with this study were present in the paper or the additional files. Our in-house dataset (PRJNA860352 and PRJNA859794) and published cohort data (the 1000IBD cohort, GSE116222, GSE134881, GSE111761) could be downloaded from the NCBI Gene Expression Omnibus (GEO, https://www.ncbi.nlm.nih.gov/geo/) and the 1000IBD project (https://ega-archive.org/studies/EGAS00001002702). All datasets or information generated in this study are available upon reasonable request from the corresponding author.

## Abbreviations

ADV: Adenovirus
AJC: Apical junction complex
BANF1: BAF nuclear assembly factor 1
CD: Crohn’s disease
CDAI: Crohn’s disease activity index
CRP: C-reactive protein
DAI: Disease activity index
DSS: Dextran sulfate sodium
FD4: 4 kD fluorescein isothiocyanate-dextran
GHAS: Geboes histology activity score
IL: Interleukin
IP: Immunoaffinity purification
qPCR: Quantitative real-time polymerase chain reaction
SES-CD: Simple endoscopic score for Crohn’s disease
TEER: Trans-epithelium electrical resistance
TEM: Transmission electron microscopy
TMIGD1: Transmembrane and immunoglobulin domain-containing protein 1
*Tmigd1*^*INT-KO*^: Intestinal-specific *Tmigd1* knockout
TNBS: 2,4,6-Trinitrobenzenesulfonic acid
TNF-α: Tumor necrosis factor α
ZO1: Zonula occludens 1
