# Supplementary material for: Decreased TMIGD1 aggravates colitis and intestinal barrier dysfunction via the BANF1-NF-κB pathway in Crohn’s disease
Source: BMC Med. 2023 Aug 4;21:287. doi: 10.1186/s12916-023-02989-2 (PMC10403950; doi:10.1186/s12916-023-02989-2)
Supplement: Supplementary file 1 — Additional file 1: Table S1. Antibodies. Table S2. Primers used in qPCR. Table S3. Clinical characteristics of patients with CD and healthy individuals in transcriptome sequencing. Table S4. Clinical characteristics of patients with CD and healthy individuals analyzed for TMIGD1 expression. Table S5. Clinical characteristics of patients with CD and healthy individuals analyzed for IHC staining of TMIGD1. Table S6. Clinical characteristics of anti-TNF responding and non-responding patients with CD before anti-TNF treatment. Fig. S1. Verification of Tmigd1INT-KO mice. Fig. S2. Chemically induced colitis in Tmigd1INT-KO mice show more severe inflammation. Fig. S3. Chemically induced colitis in Tmigd1INT-KO mice show more severe barrier dysfunction. Fig. S4. TMIGD1 is downregulated after TNF-α stimulation. Fig. S5. TMIGD1 modulates barrier function and inflammation. Fig. S6. TMIGD1 binds to BANF1. Fig. S7. TMIGD1 modulates BANF1 and inactivates NF-κB pathway. Fig. S8. BANF1 is crucial for TMIGD1 to maintain barrier function and inhibit inflammation. Fig. S9. Restoring TMIGD1 and BANF1 repairs barrier function and attenuates inflammation. Fig. S10. The proposed model for the landscape of TMIGD1-BANF1-NF-κB pathway in CD. [file 12916_2023_2989_MOESM1_ESM.docx]

**Additional file 1**

Table S1. Antibodies

Table S2. Primers used in qPCR

Table S3. Clinical characteristics of patients with CD and healthy individuals in transcriptome sequencing

Table S4. Clinical characteristics of patients with CD and healthy individuals analyzed for TMIGD1 expression

Table S5. Clinical characteristics of patients with CD and healthy individuals analyzed for IHC staining of TMIGD1

Table S6. Clinical characteristics of anti-TNF responding and non-responding patients with CD before anti-TNF treatment

Fig. S1. Verification of *Tmigd1^INT-KO^* mice

Fig. S2. Chemically induced colitis in *Tmigd1^INT-KO^* mice show more severe inflammation

Fig. S3. Chemically induced colitis in *Tmigd1^INT-KO^* mice show more severe barrier dysfunction

Fig. S4. TMIGD1 is downregulated after TNF-α stimulation

Fig. S5. TMIGD1 modulates barrier function and inflammation

Fig. S6. TMIGD1 binds to BANF1

Fig. S7. TMIGD1 modulates BANF1 and inactivates NF-κB pathway

Fig. S8. BANF1 is crucial for TMIGD1 to maintain barrier function and inhibit inflammation

Fig. S9. Restoring TMIGD1 and BANF1 repairs barrier function and attenuates inflammation

Fig. S10. The proposed model for the landscape of TMIGD1-BANF1-NF-κB pathway in CD

**Table S1.** **Antibodies**

| Antibodies | Application | Cat. | Company |
| --- | --- | --- | --- |
| TMIGD1 | WB (1:500), IHC (1:200), IF (1:200) | #ABC938 | Millipore |
| BANF1 | WB (1:500), IHC (1:100), IF (1:100), IP (1:50) | sc-166324 | Santa Cruz |
| BANF1  β-Actin | WB (1:1000)  WB (1:1000) | ab129074  #4970 | Abcam  CST |
| CLDN3 | WB (1:1000), IF (1:100) | ab214487 | Abcam |
| CLDN4 | WB (1:1000), IF (1:100) | ab53156 | Abcam |
| ZO-1 | WB (1:1000), IF (1:100) | ab276131 | Abcam |
| E-cadherin | WB (1:1000), IF (1:200) | #3195 | CST |
| MPO | IF (1:100) | ab208670 | Abcam |
| CD4 | IHC (1:1000) | ab183685 | Abcam |
| IgG  IgG | IP (1:250)  IP (1:250) | #3900  #5415 | CST  CST |
| Flag  Flag | IP (1:50)  IP (1:50) | #14793  #8146 | CST  CST |
| GST | WB (1:100) | #2625 | CST |
| NF-κB Pathway Antibody Kit | WB (1:1000) | #9936 | CST |
| Lamin B1 | WB (1:1000) | ab133741 | Abcam |
| HRP Goat Anti-Rabbit IgG (H+L) | WB (1:2000) | #7074 | CST |
| HRP Goat Anti-Mouse IgG (H+L) | WB (1:2000) | #7076 | CST |
| SignalStain® Boost IHC Detection Reagent (HRP, Rabbit)  SignalStain® Boost IHC Detection Reagent (HRP, Mouse)  Anti-mouse IgG (H+L), F(ab')2 Fragment (Alexa Fluor® 555 Conjugate) | IHC  IHC  IF (1:500) | #8114  #8125  #4409 | CST  CST  CST |
| Anti-rabbit IgG (H+L), F(ab')2 Fragment (Alexa Fluor® 488 Conjugate) | IF (1:500) | #4412 | CST |

CST, Cell Signaling Technology

**Table S2. Primers used in qPCR**

| Gene | | Forward Primer | | Reverse Primer |
| --- | --- | --- | --- | --- |
| Human TMIGD1 | CAGTTGAGGAAGGCAGTAATGT | | CGGCTTTTCTCTAAATCGAGGAG | |
| Human BANF1 | GTGGCTTGAGGTATCCGCAG | | TGCCCAGGACTTCACCAATC | |
| Human β-Actin | CTAAGTCATAGTCCGCCTAGAAGCA | | TGGCACCCAGCACAATGAA | |
| Human IL-1β | CAGAAGTACCTGAGCTCGCC | | AGATTCGTAGCTGGATGCCG | |
| Human IL-6  Human IL-23A  Human TNF-α | TTCGGTCCAGTTGCCTTCTC  CCCAAGGACTCAGGGACAAC  GCTGCACTTTGGAGTGATCG | | CTGAGATGCCGTCGAGGATG  AGAGAAGGCTCCCCTGTGAA  TCACTCGGGGTTCGAGAAGA | |
| Human IFN-γ  Human CLDN3  Human CLDN4  Human ZO-1  Human E-cadherin | ACTGACTTGAATGTCCAACGC  TCGGCCAACACCATTATCCG  CCTGGAGACTGATCCCCTCT  CAACATACAGTGACGCTTCACA  TGGACCGAGAGAGTTTCCCT | | TATTGCAGGCAGGACAACCA  CCGTGTACTTCTTCTCGCGT  AGGACTTCCAAGGGTGAAGC  CACTATTGACGTTTCCCCACTC  TTAGCCTCGTTCTCAGGCAC | |
| Mouse TMIGD1 | GGTGTCCAAGCATCTCTGGAA | | CCATTGTCACTTTCGTTGATGGG | |
| Mouse BANF1 | GCATAAAACGACCCGGAAGC | | GATCAGGCTTAACCGGAGGC | |
| Mouse β-Actin | CCACTGTCGAGTCGCGT | | CCACGATGGAGGGGAATACAG | |
| Mouse IL-1β | GCAGTGGTTCGAGGCCTAAT | | TCATCACTGTCAAAAGGTGGCA | |
| Mouse IL-6  Mouse TNF-α | GTCCTTCCTACCCCAATTTCCA  GTAGCCCACGTCGTAGCAAA | | TAACGCACTAGGTTTGCCGA  ACAAGGTACAACCCATCGGC | |

**Table S3. Clinical characteristics of patients with CD and healthy individuals in transcriptome sequencing**

|  | Sex | Age (years) | Sampling  location | CDAI | CRP (mg/L) | SES-CD | GHAS |
| --- | --- | --- | --- | --- | --- | --- | --- |
| CD1 | male | 24.0 | colon | 240.4 | 42.64 | 5 | 11 |
| CD2 | female | 37.0 | colon | 368.6 | 163.03 | 8 | 12 |
| CD3 | male | 38.0 | colon | 442.2 | 61.22 | 8 | 11 |
| CD4 | male | 42.0 | colon | 465.0 | 134.88 | 11 | 15 |
| CD5 | male | 34.0 | colon | 366.0 | 189.15 | 9 | 13 |
| CD6 | male | 17.0 | colon | 487.8 | 107.31 | 10 | 14 |
| CD7 | male | 19.0 | colon | 243.4 | 27.04 | 3 | 10 |
| NC1 | male | 29.0 | colon | - | - | - | - |
| NC2 | female | 63.0 | colon | - | - | - | - |
| NC3 | male | 22.0 | colon | - | - | - | - |
| NC4 | female | 69.0 | colon | - | - | - | - |
| NC5 | female | 48.0 | colon | - | - | - | - |
| NC6 | male | 55.0 | colon | - | - | - | - |
| NC7 | female | 32.0 | colon | - | - | - | - |
| NC8 | male | 37.0 | colon | - | - | - | - |
| NC9 | female | 31.0 | colon | - | - | - | - |
| NC10 | female | 66.0 | colon | - | - | - | - |

**Table S4. Clinical characteristics of patients with CD and healthy individuals analyzed for TMIGD1 mRNA expression**

|  | Patients with CD (n=64) | Healthy controls (n=55) |
| --- | --- | --- |
| Male, n (%) | 46 (71.9) | 38 (69.1) |
| Female, n (%) | 18 (28.1) | 17 (30.9) |
| Age, years, median (IQR) | 27.0 (18.0-32.0) | 28.0 (20.0-35.0) |
| Disease location |  |  |
| L1 (ileal disease), n (%) | 2 (3.1) | - |
| L2 (colonic disease), n (%) | 4 (6.3) | - |
| L3 (ileocolonic disease), n (%) | 58 (90.6) | - |
| Sampling location |  |  |
| Terminal ileum, n (%) | 3 (4.7) | 0 (0.0) |
| Ascending colon, n (%) | 9 (14.1) | 10 (18.2) |
| Transverse colon, n (%) | 38 (59.4) | 34 (61.8) |
| Descending colon, n (%)  Sigmoid colon, n (%) | 11 (17.2)  3 (4.7) | 8 (14.5)  3 (5.5) |
| Disease behavior |  |  |
| B1 (non-stricturing, non-penetrating), n (%) | 37 (57.8) | - |
| B2 (stricturing), n (%) | 17 (26.6) | - |
| B3 (penetrating), n (%) | 10 (15.6) | - |
| Perianal disease, n (%) | 42 (65.6) | - |
| Presence of extraintestinal manifestations, n (%) | 5 (7.8) | - |
| Medication |  |  |
| 5-Aminosalicyclic acid, n (%) | 19 (29.7) | - |
| Immunomodulator (e.g., Thiopurine), n (%)  Corticosteroids, n (%) | 7 (10.9)  4 (6.3) | -  - |
| Biologic agents, n (%) | 3 (4.7) | - |
| None (treatment-naive), n (%)  Abdominal surgery, n (%) | 31 (48.4)  2 (3.1) | -  - |

IQR: interquartile range

**Table S5. Clinical characteristics of patients with CD and healthy individuals analyzed for IHC staining of TMIGD1**

|  | Healthy individuals  (n=13) | Patients with CD,  CDAI＜150 (n=10) | Patients with CD,  150≤CDAI＜220 (n=12) | Patients with CD,  220≤CDAI＜450 (n=15) | Patients with CD,  450≤CDAI (n=5) |
| --- | --- | --- | --- | --- | --- |
| Male, n (%) | 7 (53.8) | 5 (50.0) | 6 (50.0) | 9 (60.0) | 3 (60.0) |
| Female, n (%) | 6 (46.2) | 5 (50.0) | 6 (50.0) | 6 (40.0) | 2 (40.0) |
| Age, years, median (IQR) | 28.0 (21.0-35.0) | 26.0 (20.0-33.0) | 27.0 (21.0-33.0) | 22.0 (16.0-27.0) | 24.0 (17.0-27.0) |
| Disease location |  |  |  |  |  |
| L1 (ileal disease), n (%) | - | 1 (10.0) | 1 (8.3) | 0 (0.0) | 0 (0.0) |
| L2 (colonic disease), n (%) | - | 0 (0.0) | 0 (0.0) | 1 (6.7) | 0 (0.0) |
| L3 (ileocolonic disease), n (%) | - | 9 (90.0) | 11 (91.7) | 14 (93.3) | 5 (100.0) |
| Sampling location |  |  |  |  |  |
| Ascending colon, n (%) | 2 (15.4) | 2 (20.0) | 2 (16.7) | 2 (13.3) | 1 (20.0) |
| Transverse colon, n (%) | 9 (69.2) | 6 (60.0) | 7 (58.3) | 8 (53.3) | 3 (60.0) |
| Descending colon, n (%) | 2 (15.4) | 2 (20.0) | 3 (25.0) | 5 (33.3) | 1 (20.0) |
| Disease behavior |  |  |  |  |  |
| B1 (non-stricturing, non-penetrating), n (%) | - | 6 (60.0) | 6 (50.0) | 9 (60.0) | 2 (40.0) |
| B2 (stricturing), n (%) | - | 3 (30.0) | 3 (25.0) | 2 (13.3) | 1 (20.0) |
| B3 (penetrating), n (%) | - | 1 (10.0) | 3 (25.0) | 4 (26.7) | 2 (40.0) |
| Perianal disease, n (%) | - | 5 (50.0) | 9 (75.0) | 11 (73.3) | 4 (80.0) |
| Presence of extraintestinal manifestations, n (%) | - | 1 (10.0) | 1 (8.3) | 1 (6.7) | 1 (20.0) |
| Medication |  |  |  |  |  |
| 5-Aminosalicyclic acid, n (%) | - | 2 (20.0) | 2 (16.7) | 2 (13.3) | 1 (20.0) |
| Immunomodulator (e.g., Thiopurine), n (%)  Biologic agents, n (%) | -  - | 0 (0.0)  0 (0.0) | 0 (0.0)  0 (0.0) | 0 (0.0)  0 (0.0) | 0 (0.0)  0 (0.0) |
| None (treatment-naive), n (%)  Abdominal surgery, n (%) | -  - | 8 (80.0)  0 (0.0) | 10 (83.3)  0 (0.0) | 13 (86.7)  0 (0.0) | 4 (80.0)  0 (0.0) |

IQR: interquartile range.

**Table S6. Clinical characteristics of anti-TNF responding and non-responding patients with CD before anti-TNF treatment**

|  | Responders  (n=10) | Non-responders (n=10) | *p* |
| --- | --- | --- | --- |
| Male, n (%) | 6 (60.0) | 7 (70.0) | ns |
| Female, n (%) | 4 (40.0) | 3 (30.0) | ns |
| Age, years, median (IQR) | 43.0 (36.0-50.0) | 46.5 (38.0-50.0) | ns |
| Disease location |  |  |  |
| L1 (ileal disease), n (%) | 1 (10.0) | 1 (10.0) | - |
| L2 (colonic disease), n (%) | 1 (10.0) | 1 (10.0) | - |
| L3 (ileocolonic disease), n (%) | 8 (80.0) | 8 (80.0) | - |
| Sampling location |  |  |  |
| Ascending colon, n (%) | 3 (30.0) | 2 (20.0) | - |
| Transverse colon, n (%) | 5 (50.0) | 5 (50.0) | - |
| Descending colon, n (%) | 2 (20.0) | 3 (30.0) | - |
| Disease behavior |  |  |  |
| B1 (non-stricturing, non-penetrating), n (%) | 5 (50.0) | 4 (40.0) | - |
| B2 (stricturing), n (%) | 3 (30.0) | 4 (40.0) | - |
| B3 (penetrating), n (%) | 2 (20.0) | 2 (20.0) | - |
| Perianal disease, n (%) | 5 (50.0) | 7 (70.0) | - |
| Presence of extraintestinal manifestations, n (%) | 1 (10.0) | 1 (10.0) | - |
| Medication |  |  |  |
| 5-Aminosalicyclic acid, n (%) | 10 (100.0) | 10 (100.0) | - |
| Immunomodulator (e.g., Thiopurine), n (%)  Corticosteroids, n (%) | 9 (90.0)  9 (90.0) | 9 (90.0)  9 (90.0) | -  - |
| Biologic agents, n (%)  None (treatment-naive), n (%) | 0 (0.0)  0 (0.0) | 0 (0.0)  0 (0.0) | -  - |
| Abdominal surgery, n (%) | 0 (0.0) | 0 (0.0) | - |
| CDAI, median (IQR) | 259.5 (221.5-290.0) | 254.5 (223.0-294.0) | ns |
| SES-CD, median (IQR) | 20.0 (13.0-24.0) | 22.0 (14.5-25.0) | ns |
| GHAS, median (IQR) | 7.0 (6.0-8.0) | 6.5 (6.0-8.0) | ns |
| CRP, mg/L, median (IQR) | 18.8 (11.7-46.9) | 22.1 (6.0-53.7) | ns |

IQR: interquartile range; CDAI: Crohn's disease activity index; SES-CD: simple endoscopic score for Crohn's disease; GHAS: Geboes histology activity score; CRP: C-reactive protein; ns: no significance.


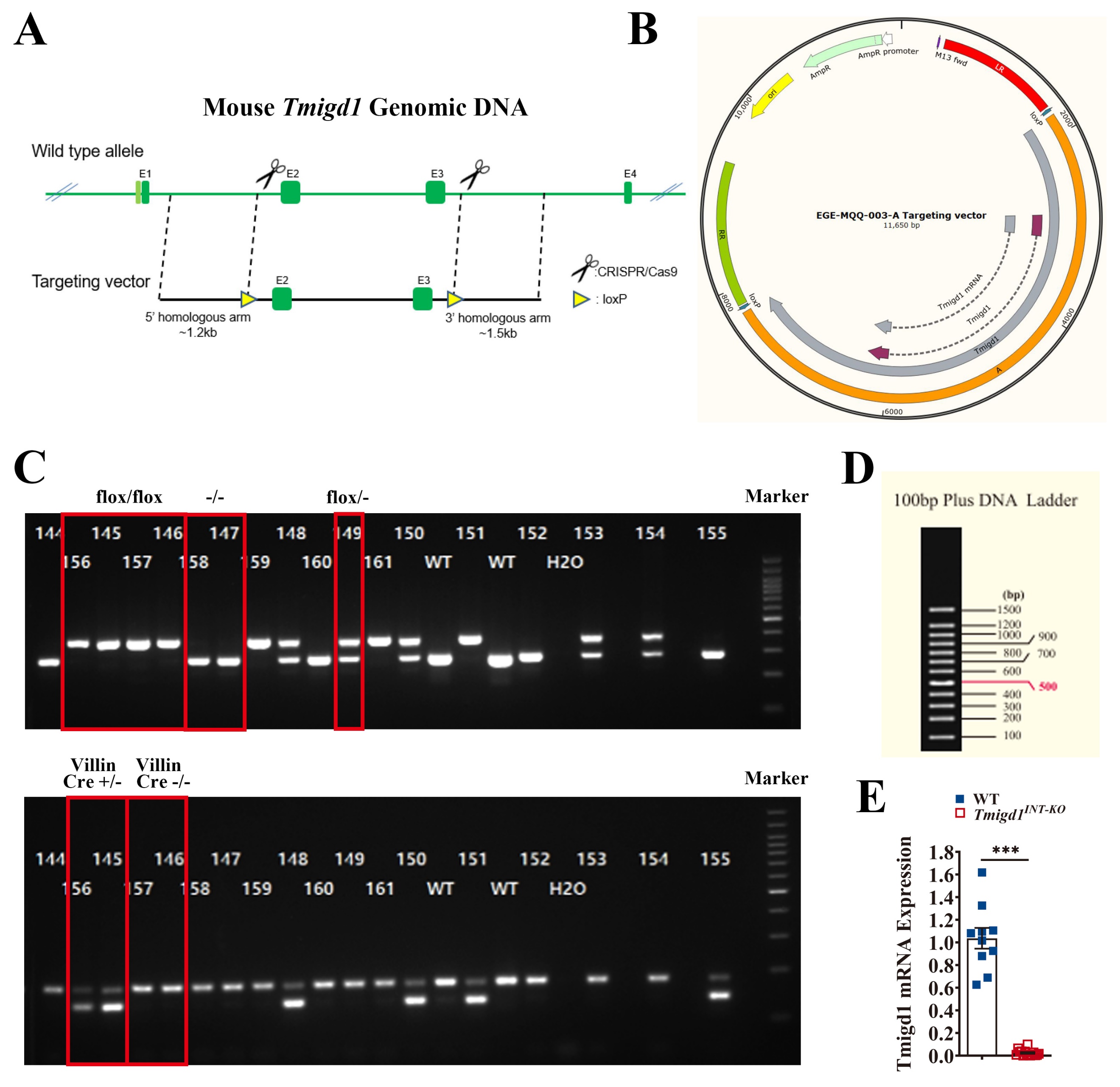


**Fig. S1.** **Verification of *Tmigd1^INT-KO^* mice**

(A) Schematic illustration of the strategy to generate *Tmigd1^flox/flox^* mice. SgRNAs were designed to delete exons 2 to 3 of the Tmigd1 gene. (B) Targeting plasmid in the generation of *Tmigd1^flox/flox^* mice. (C) Genomic identification of *Tmigd1^flox/flox^* mice (e.g., No. 145, 146, 156, 157), *Tmigd1^flox/-^* (e.g., No. 149) and wild type (e.g., No. 147, 158) (up). Genomic identification of mice with villin cre (e.g., No. 145, 156) and without villin cre (e.g., No. 146, 157) (down). That is to say, *Tmigd1^INT-KO^* mice (e.g., No. 145, 156), flox control (WT) mice (e.g., No. 146, 157) and wild type mice (e.g., No. 147, 158) were generated. (D) The legend of DNA marker. (E) The expression of Tmigd1 mRNA in *Tmigd1^INT-KO^* mice (n=15) and WT mice (n=10).

Data are expressed as mean ± SEM. *** *p*<0.001.


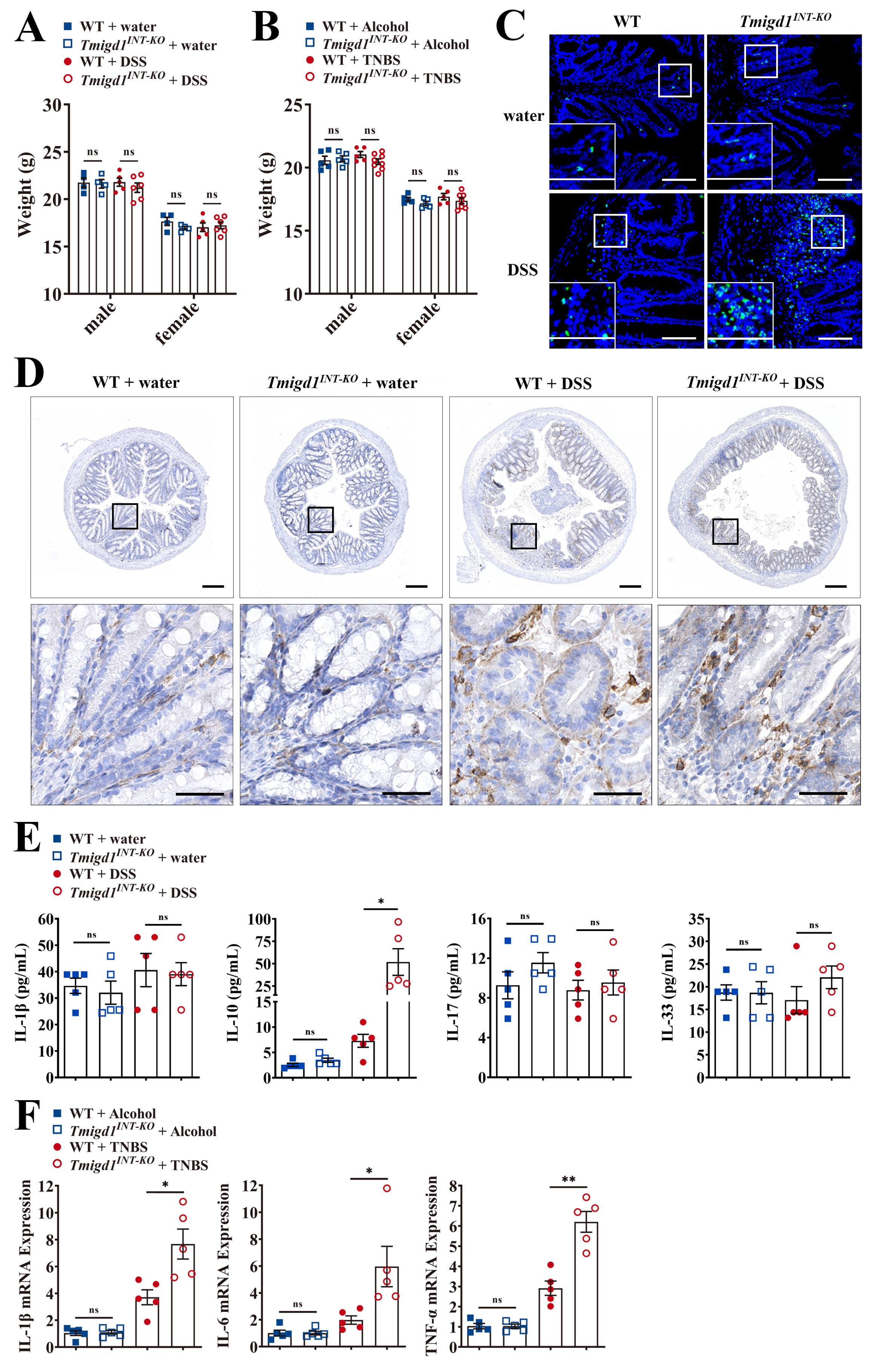


**Fig. S2. Chemically induced colitis in *Tmigd1^INT-KO^* mice show more severe inflammation**

(A) Body weight at Day 0; WT+water (male, n=4; female, n=4), *Tmigd1^INT-KO^*+water (male, n=4; female, n=4), WT+DSS (male, n=5; female, n=5), *Tmigd1^INT-KO^*+DSS (male, n=6; female, n=6). (B) Body weight at Day 0; WT+Alcohol (male, n=5; female, n=5), *Tmigd1^INT-KO^*+Alcohol (male, n=5; female, n=5), WT+TNBS (male, n=5; female, n=5), *Tmigd1^INT-KO^*+TNBS (male, n=8; female, n=7). (C) Representative images of MPO-stained colon sections. Scale bars, 100 μm. (D) Representative images of CD4-stained colon sections. Scale bars, 200 μm (top) and 50 μm (bottom). (E) Serum IL-1β, IL-10, IL-17, and IL-33 concentrations were tested using multiELISA; Every group, n=5. (F) Pro-inflammatory cytokines mRNA in colonic tissues. Every group, n=5.

Data are expressed as mean ± SEM. ns, no significance, * *p*<0.05, ** *p*<0.01, *** *p*<0.001.


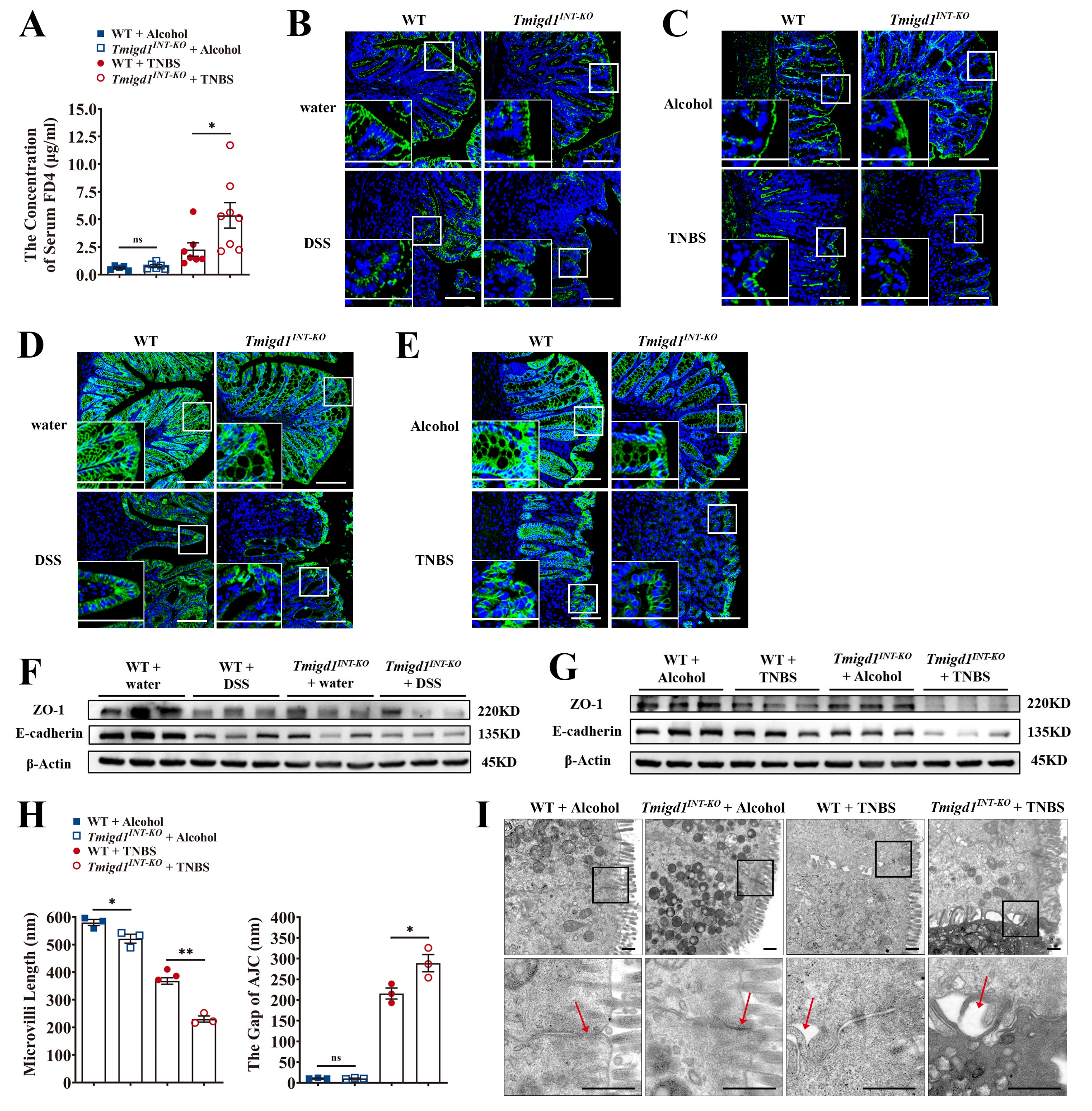


**Fig. S3. Chemically induced colitis in *Tmigd1^INT-KO^* mice show more severe barrier dysfunction**

(A) The concentration of FD4 in serum; WT+Alcohol (n=5), *Tmigd1^INT-KO^*+Alcohol (n=6), WT+TNBS (n=7), *Tmigd1^INT-KO^*+TNBS (n=8). (B-C) Representative images of ZO-1-stained colon sections. Scale bars, 100 μm. (D-E) Representative images of E-cadherin-stained colon sections. Scale bars, 100 μm. (F-G) The expression of AJC proteins. (H) Measurement of microvilli length and AJC gaps in colonic epithelial cells using TEM. Every group, n=3. (I) Representative TEM images of the colonic mucosa. The arrowheads indicate AJC. Scale bars, 500 nm.

Data are expressed as mean ± SEM. ns, no significance, * *p*<0.05, ** *p*<0.01.


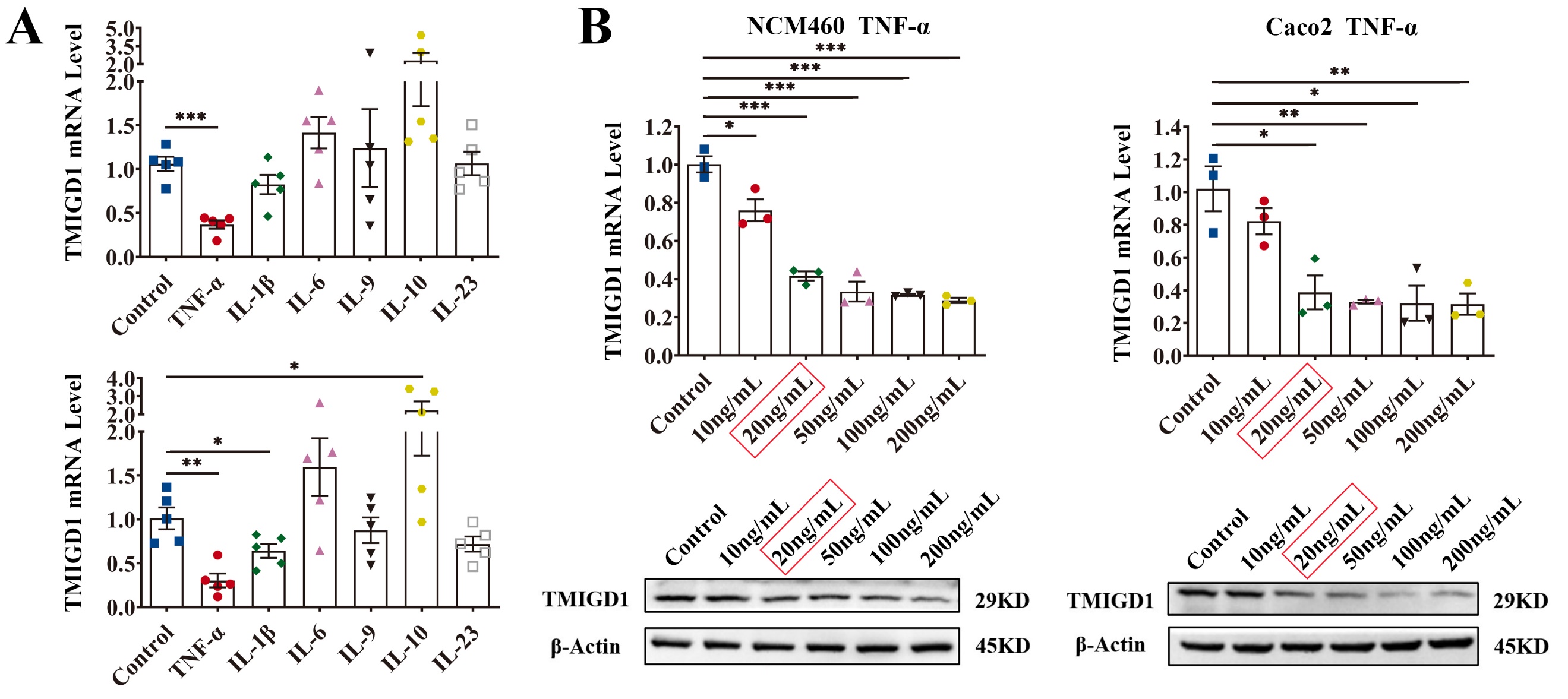


**Fig. S4. TMIGD1 is downregulated after TNF-α stimulation**

(A) TMIGD1 mRNA expression after cytokines treatment in NCM460 (up) and Caco2 cells (down) for 48 h. (B) The expression of TMIGD1 mRNA and protein in NCM460 (left) and Caco2 cells (right) after TNF-α stimulation at different concentrations for 48 h.

Data are expressed as mean ± SEM. ns, no significance, * *p*<0.05, ** *p*<0.01, *** *p*<0.001.

**
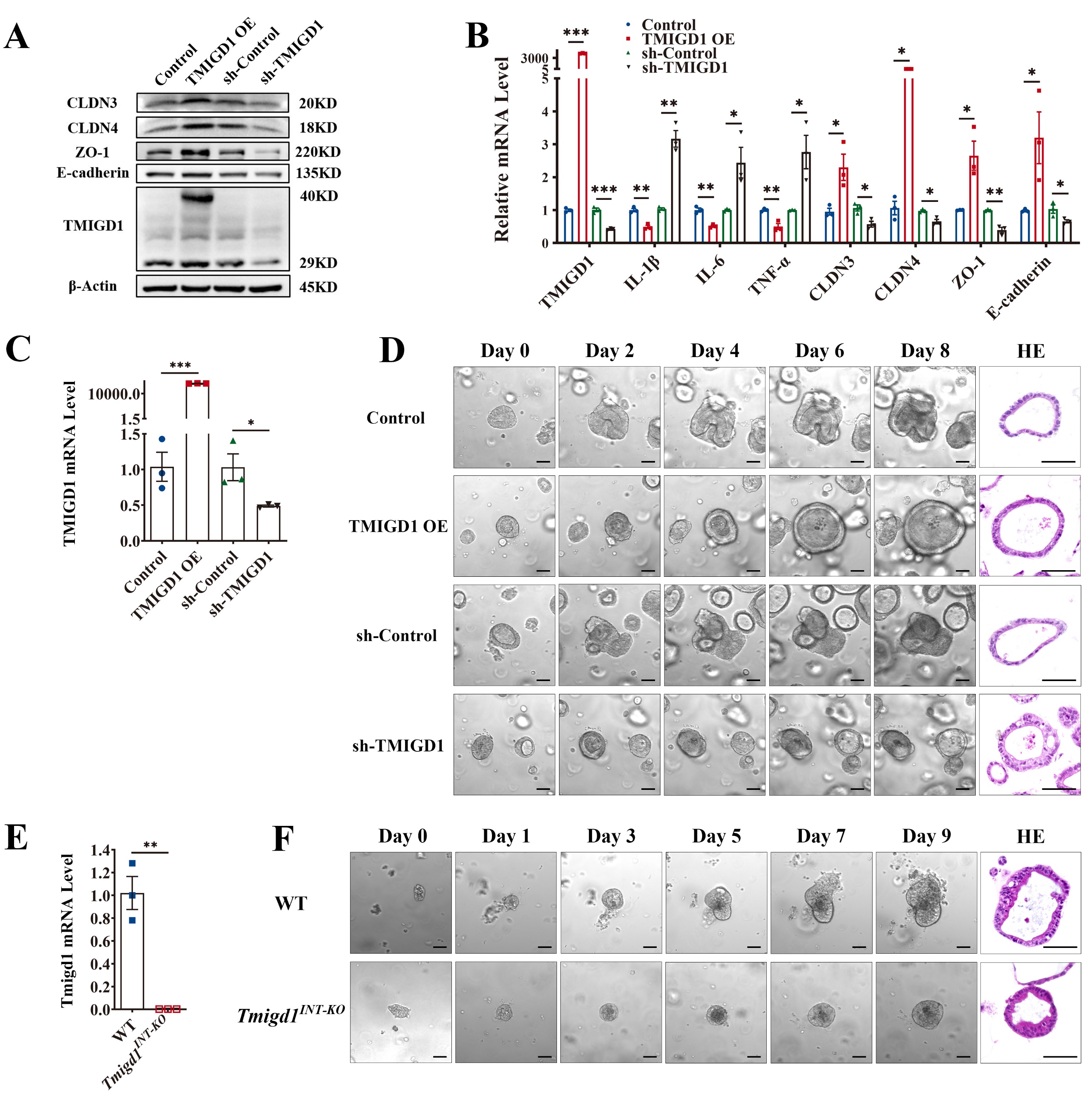
**

**Fig. S5. TMIGD1 modulates barrier function and inflammation**

(A-B) The expression of AJC proteins (A) and inflammation-relevant cytokine mRNA and AJC mRNA (B) in Caco2 cells after TNF-α stimulation. (C) The expression of TMIGD1 in human colonic organoids. (D) Cultivation of human colonic organoids and representative HE images. Scale bars, 50 μm. (E) The expression of Tmigd1 in colonic organoids generated from WT and *Tmigd1^INT-KO^* mice. (F) Cultivation of murine colonic organoids and representative HE images from WT and *Tmigd1^INT-KO^* mice. Scale bars, 50 μm.

Data are expressed as mean ± SEM. * *p*<0.05, ** *p*<0.01, *** *p*<0.001.


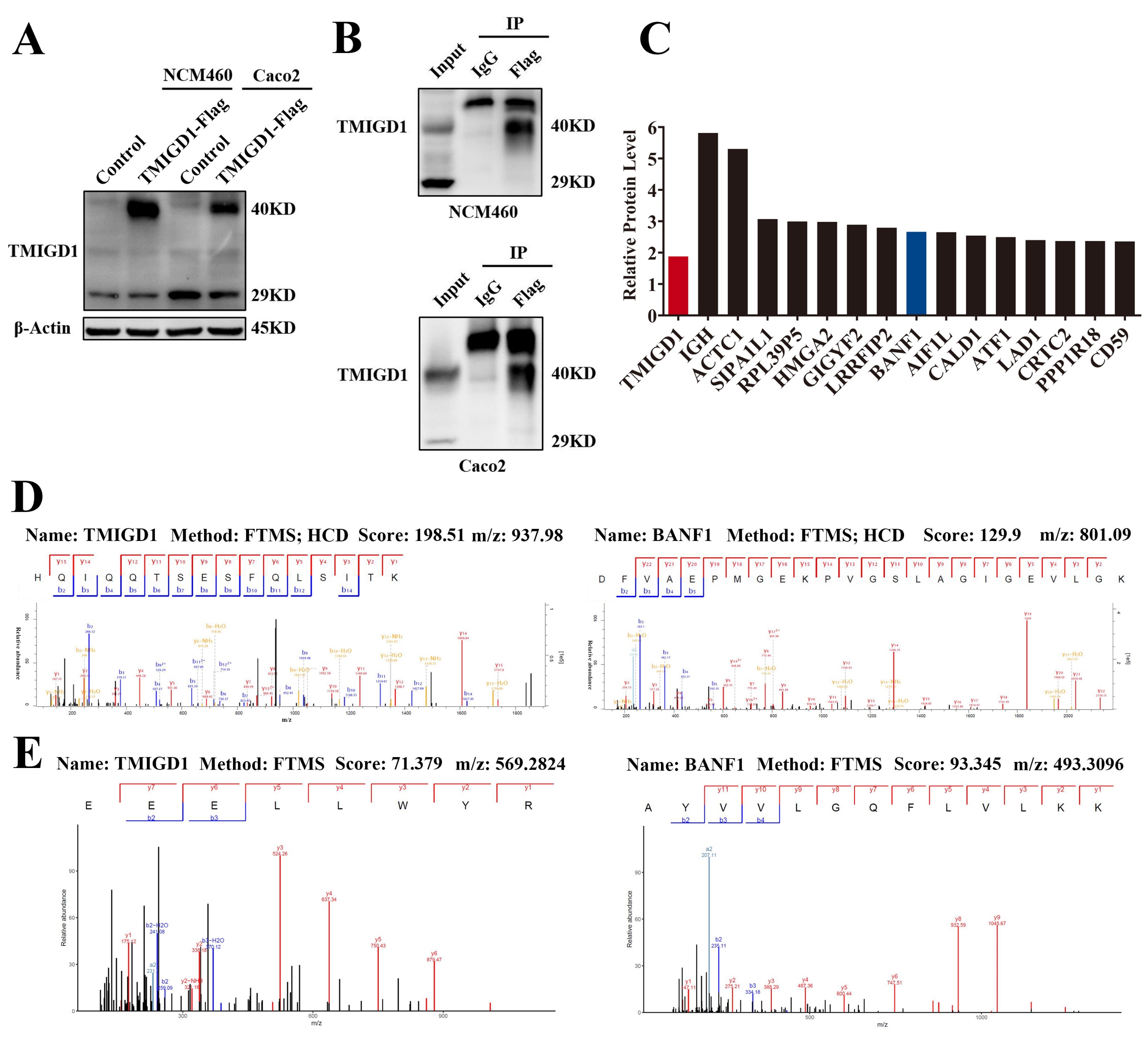


**Fig. S6.** **TMIGD1 binds to BANF1**

(A) Overexpression of FLAG-tagged TMIGD1 in NCM460 and Caco2 cells. (B) Immunoprecipitation by anti-FLAG antibody from the lysates of NCM460 or Caco2 cells transfected with lentivirus overexpressing FLAG-tagged TMIGD1. (C) Proteomic analysis shows the 15 most enriched proteins in the immunoprecipitate of Caco2 cells. (D-E) The mass spectrum of TMIGD1 protein and BANF1 protein from the immunoprecipitate of NCM460 (D) and Caco2 cells (E).


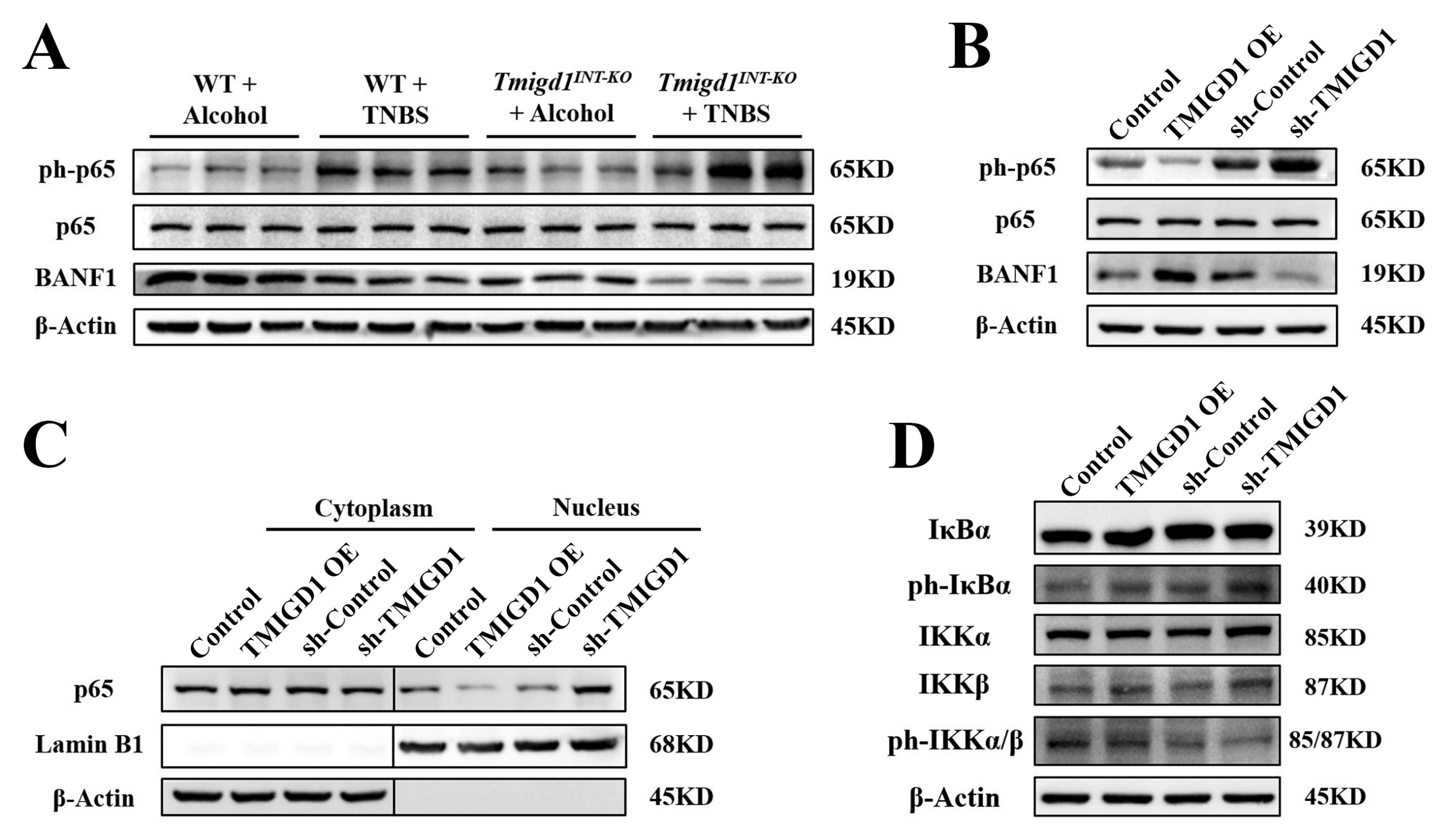


**Fig. S7.** **TMIGD1 modulates BANF1 and inactivates** **NF-κB pathway**

(A) Protein levels of BANF1, p65, and phosphorylated p65. (B) Protein levels of BANF1, p65, and phosphorylated p65 after TNF-α stimulation in Caco2 cells. (C) P65 in the cytoplasmic and nuclear fractions of Caco2 cells after TNF-α stimulation. (D) Protein levels of NF-κB pathway after TNF-α stimulation in NCM460 cells.


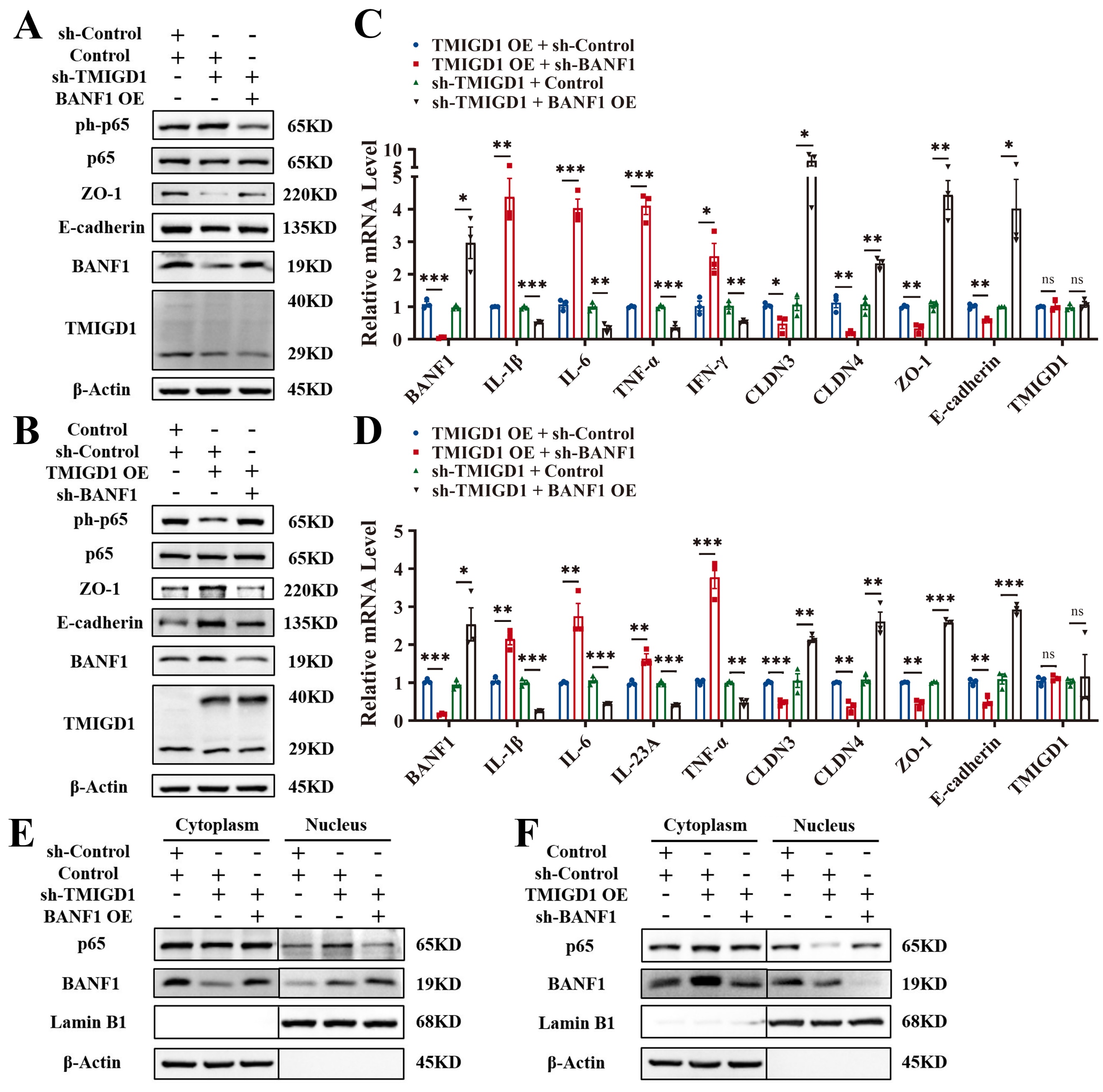


**Fig. S8. BANF1 is crucial for TMIGD1** **to maintain barrier function and inhibit inflammation**

(A-B) The protein expression of AJC, p65, and phosphorylated p65 in Caco2 cells after TNF-α stimulation. (C-D) The mRNA levels of cytokine and AJC in NCM460 cells (C) and Caco2 cells (D) after TNF-α stimulation. (E-F) BANF1 and p65 in the cytoplasmic and nuclear fractions of Caco2 cells after TNF-α stimulation.

Data are expressed as mean ± SEM. ns, no significance, * *p*<0.05, ** *p*<0.01, *** *p*<0.001.


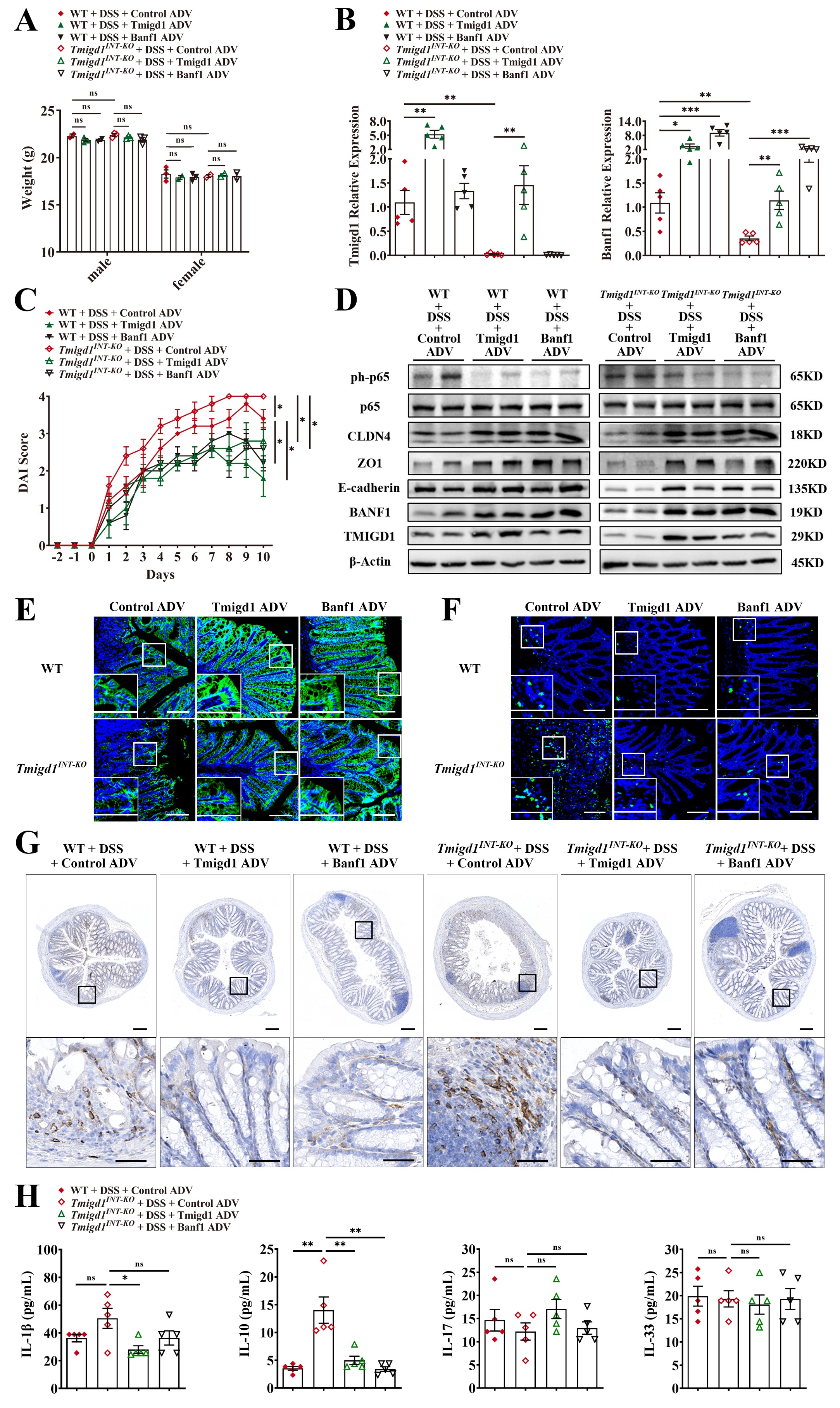


**Fig. S9. Restoring TMIGD1 and BANF1 repairs barrier function and attenuates inflammation**

(A) Body weight at Day -2; WT+DSS+Control ADV (male, n=2; female, n=3), WT+DSS+Tmigd1 ADV (male, n=3; female, n=2), WT+DSS+Banf1 ADV (male, n=2; female, n=3), *Tmigd1^INT-KO^*+DSS+Control ADV (male, n=3; female, n=2), *Tmigd1^INT-KO^* +DSS+Tmigd1 ADV (male, n=3; female, n=2) and *Tmigd1^INT-KO^*+DSS+Banf1 ADV (male, n=3; female, n=2). (B) The mRNA expression of Tmigd1 and Banf1 in colonic tissues. Every group, n=5. (C) DAI scores. Every group, n=5. (D) The protein expression of AJC, p65, and phosphorylated p65 in mice colonic tissues. (E-F) Representative images of E-cadherin-stained (E) and MPO-stained (F) colon sections. Scale bars, 100 μm. (G) Representative images of CD4-stained colon sections. Scale bars, 200 μm (top) and 50 μm (bottom). (H) Serum IL-1β, IL-10, IL-17, and IL-33 concentrations were detected using multiELISA; Every group, n=5.

Data are expressed as mean ± SEM. ns, no significance, * *p*<0.05, ** *p*<0.01, *** *p*<0.001.


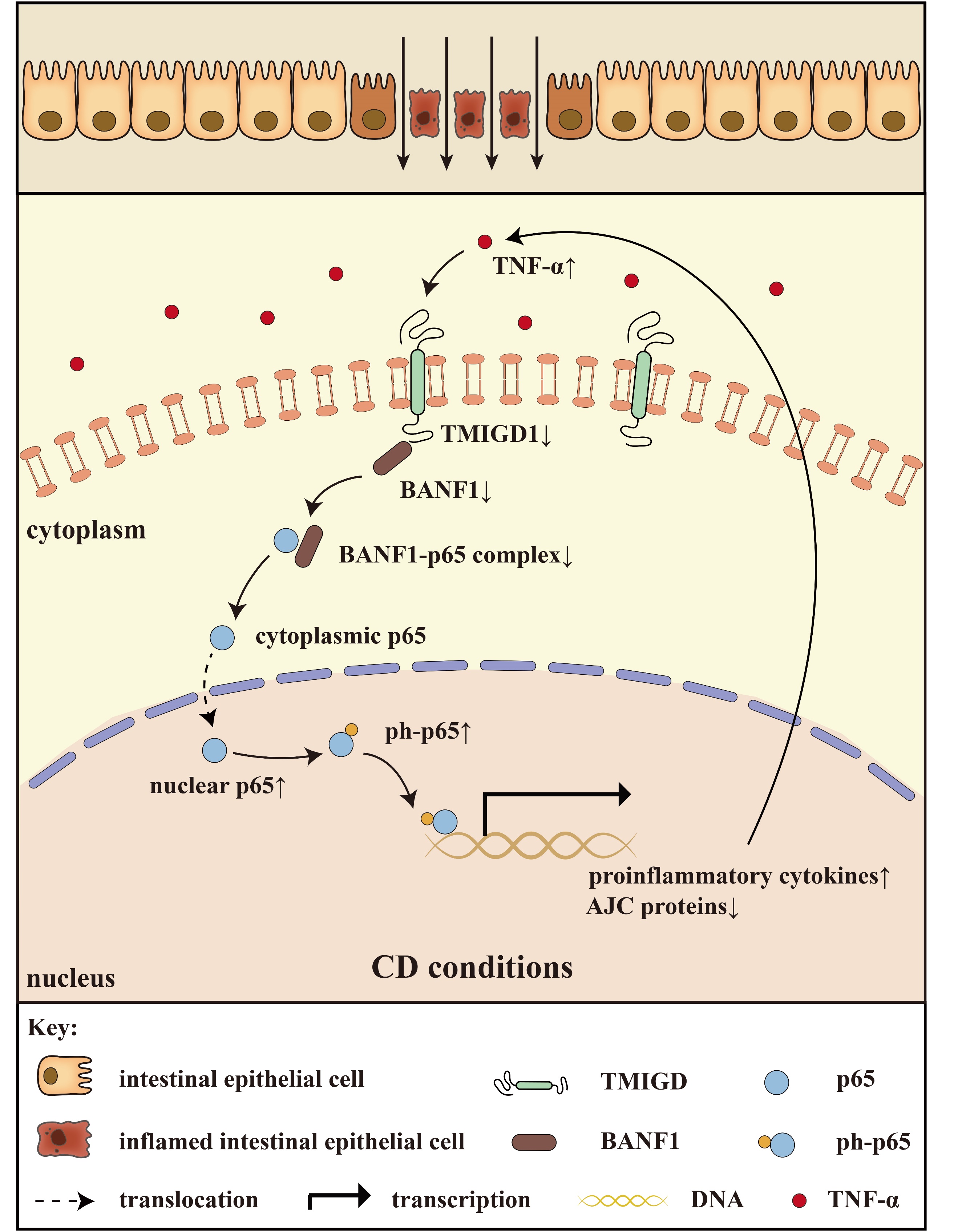


**Fig. S10. The proposed model for the landscape of TMIGD1-BANF1-NF-κB pathway in CD**
