# Supplementary material for: Decreased TMIGD1 aggravates colitis and intestinal barrier dysfunction via the BANF1-NF-κB pathway in Crohn’s disease
Source: BMC Med. 2023 Aug 4;21:287. doi: 10.1186/s12916-023-02989-2 (PMC10403950; doi:10.1186/s12916-023-02989-2)
Supplement: Supplementary file 4 — Additional file 4. The uncropped original gels or western blots from the figures. [file 12916_2023_2989_MOESM4_ESM.pptx]

## Slide 1
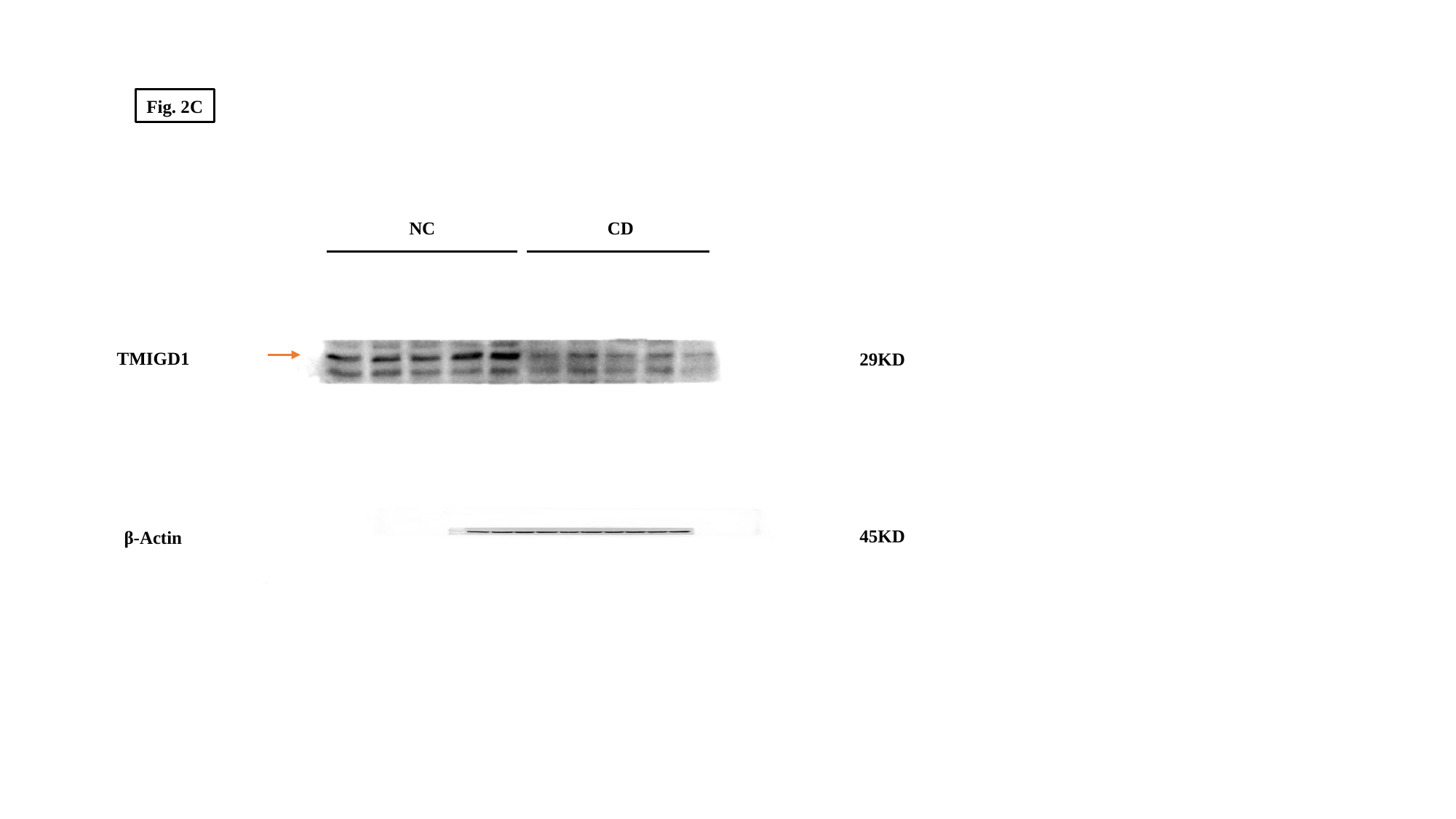

Fig. 2C
NC
CD
TMIGD1
29KD
45KD
β-Actin

## Slide 2
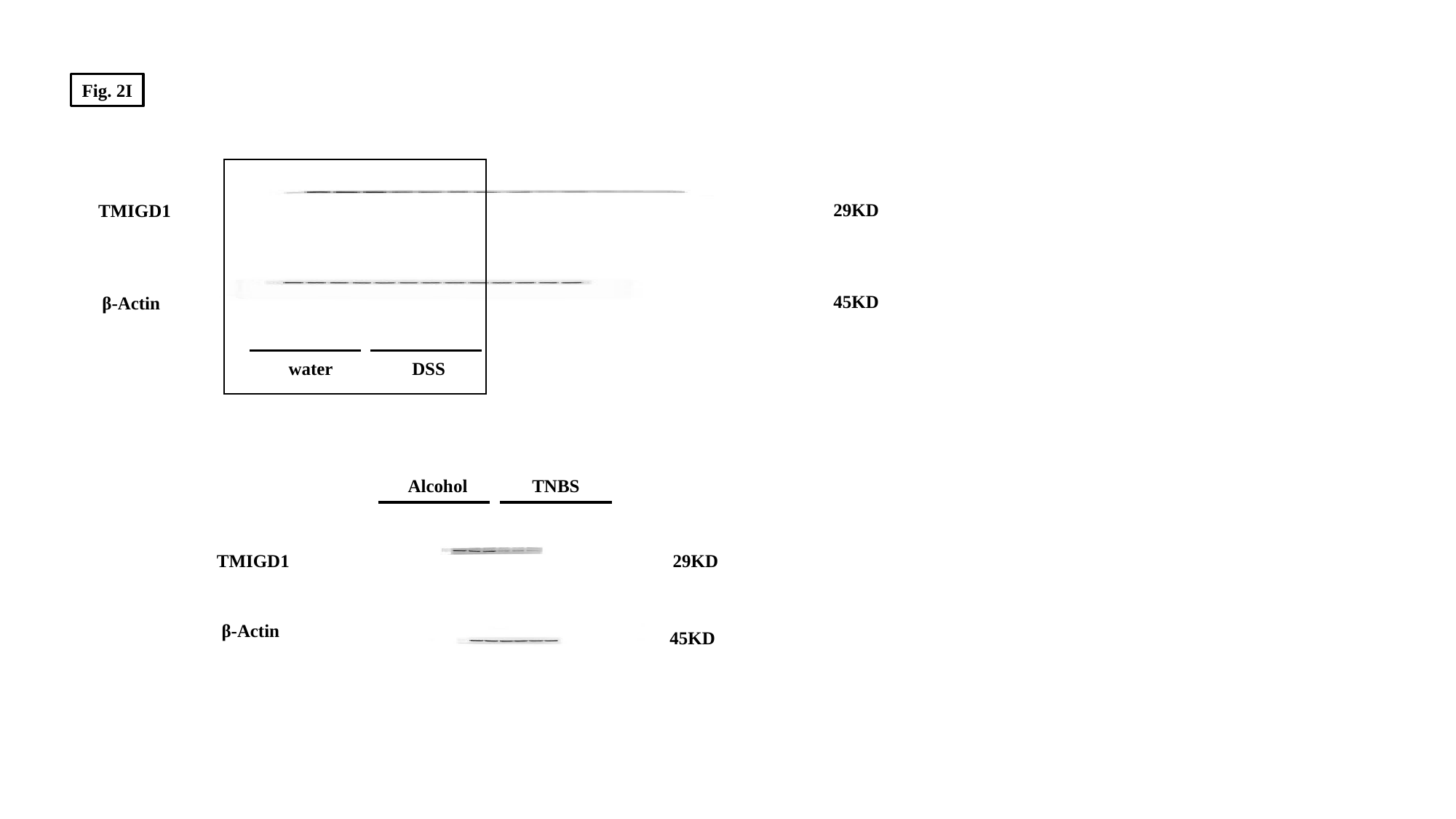

Fig. 2I
29KD
TMIGD1
45KD
β-Actin
water
DSS
Alcohol
TNBS
TMIGD1
29KD
β-Actin
45KD

## Slide 3
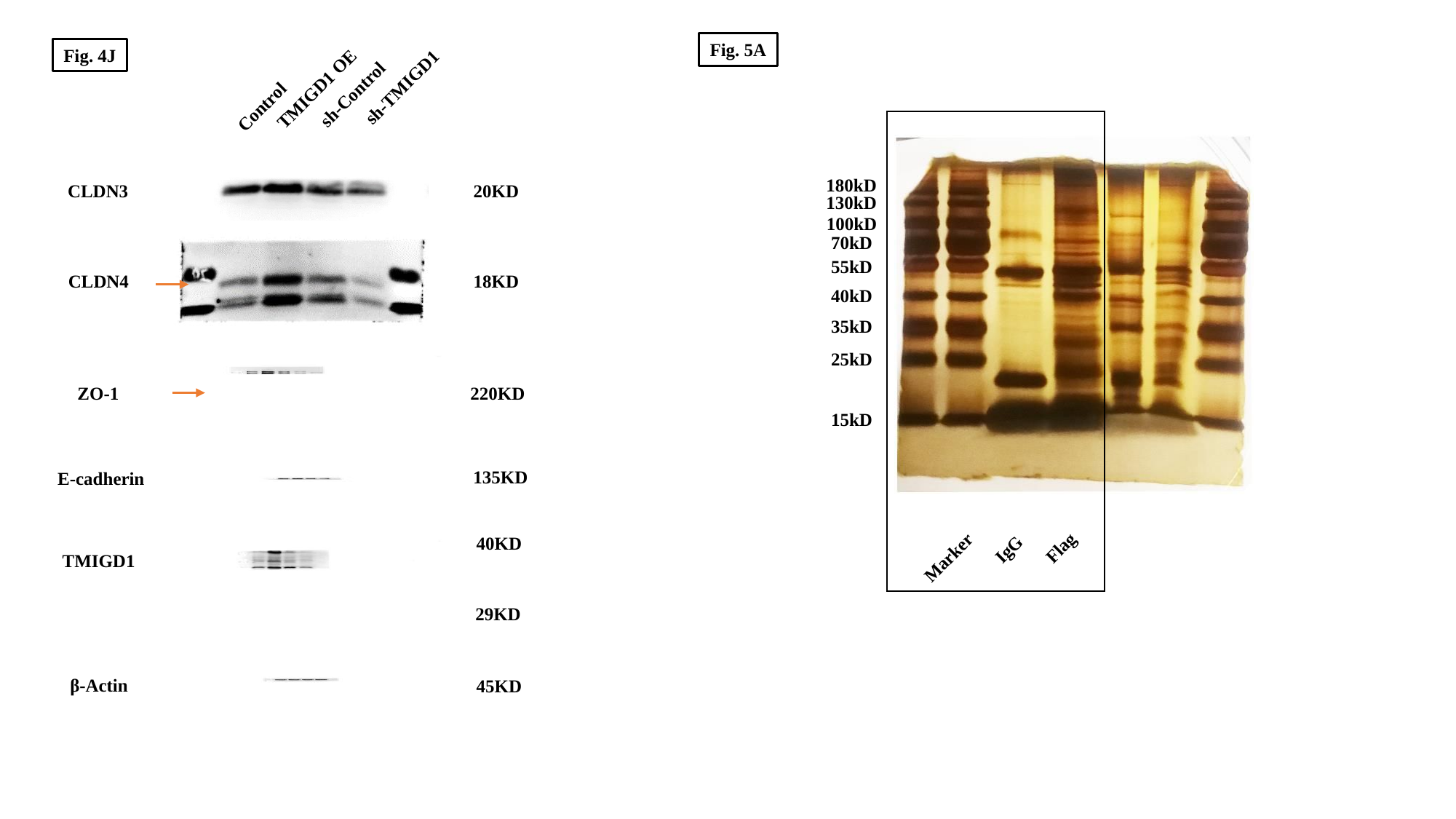

Fig. 5A
Fig. 4J
sh-TMIGD1
TMIGD1 OE
sh-Control
Control
180kD
20KD
CLDN3
130kD
100kD
70kD
55kD
18KD
CLDN4
40kD
35kD
25kD
220KD
ZO-1
15kD
135KD
E-cadherin
40KD
Flag
IgG
Marker
TMIGD1
29KD
β-Actin
45KD

## Slide 4
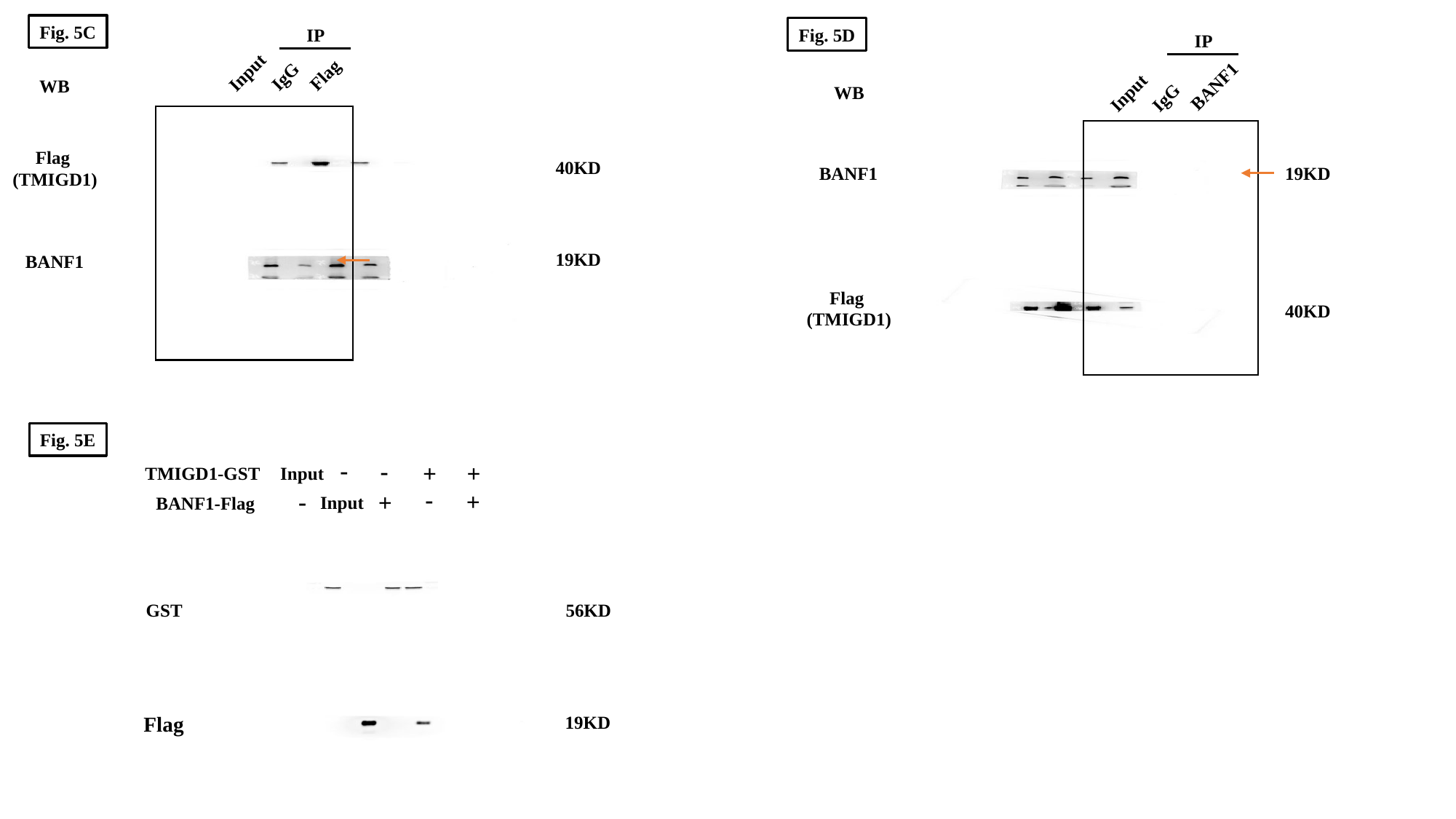

Fig. 5C
Fig. 5D
IP
IP
Input
Flag
IgG
WB
BANF1
WB
Input
IgG
Flag
(TMIGD1)
40KD
BANF1
19KD
19KD
BANF1
Flag
(TMIGD1)
40KD
Fig. 5E
-
-
 Input
+
+
TMIGD1-GST
-
-
+
+
Input
BANF1-Flag
GST
56KD
Flag
19KD

## Slide 5
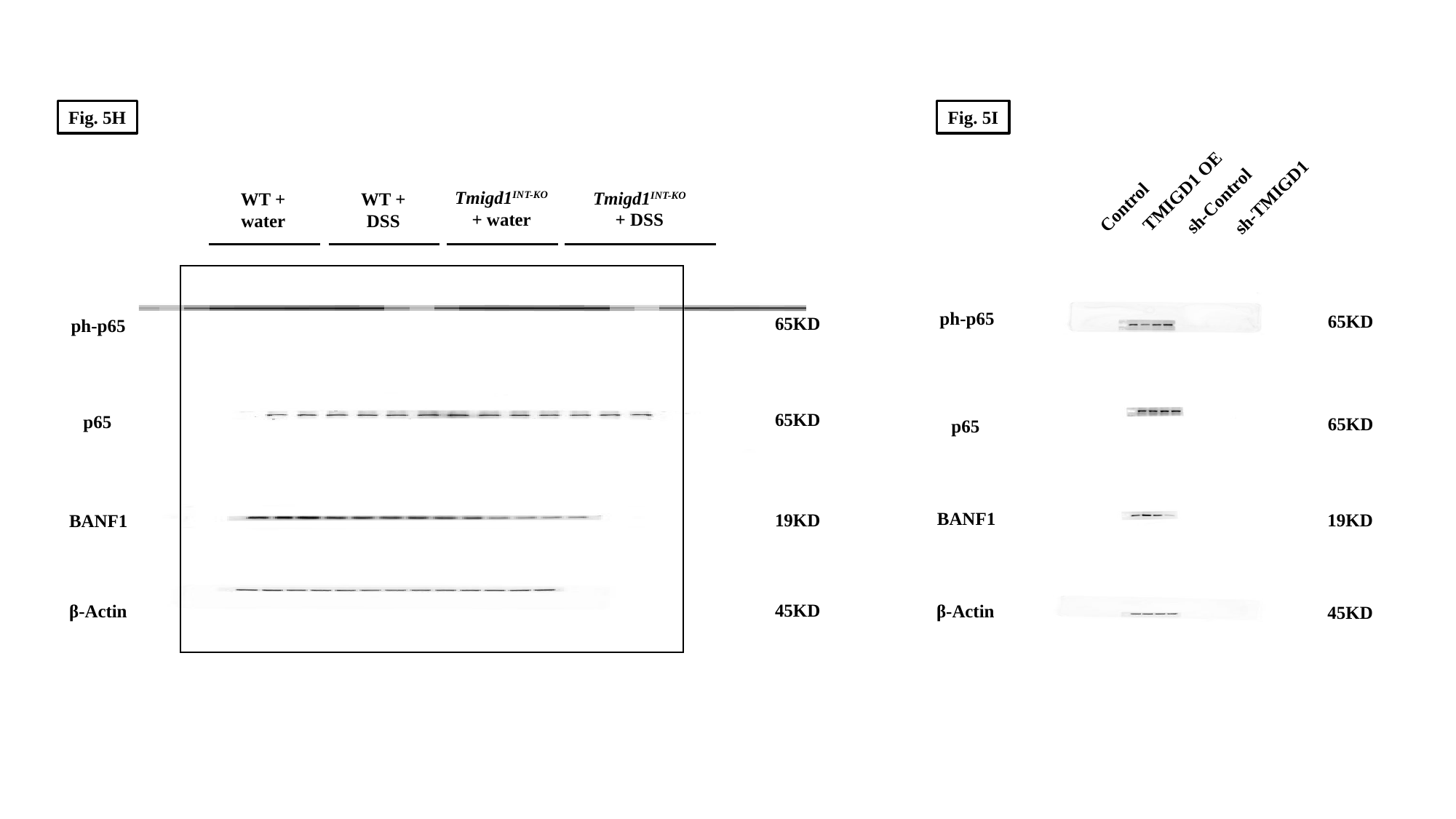

Fig. 5H
Fig. 5I
TMIGD1 OE
sh-TMIGD1
Tmigd1INT-KO
+ water
Tmigd1INT-KO
+ DSS
WT +
water
WT +
DSS
sh-Control
Control
ph-p65
65KD
65KD
ph-p65
65KD
p65
65KD
p65
BANF1
19KD
19KD
BANF1
45KD
β-Actin
β-Actin
45KD

## Slide 6
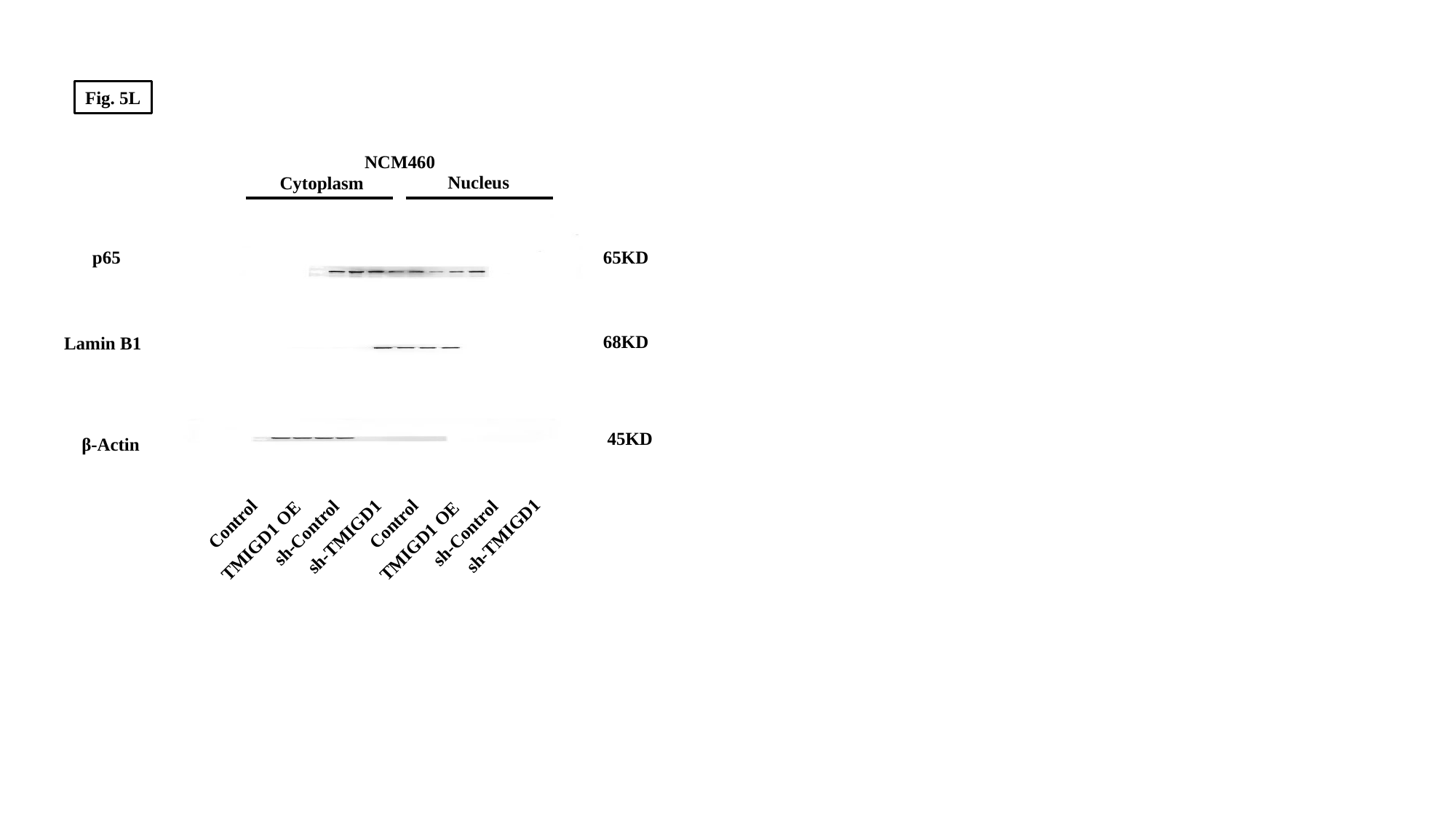

Fig. 5L
NCM460
Nucleus
Cytoplasm
65KD
p65
68KD
Lamin B1
45KD
β-Actin
Control
Control
sh-Control
sh-Control
sh-TMIGD1
sh-TMIGD1
TMIGD1 OE
TMIGD1 OE

## Slide 7
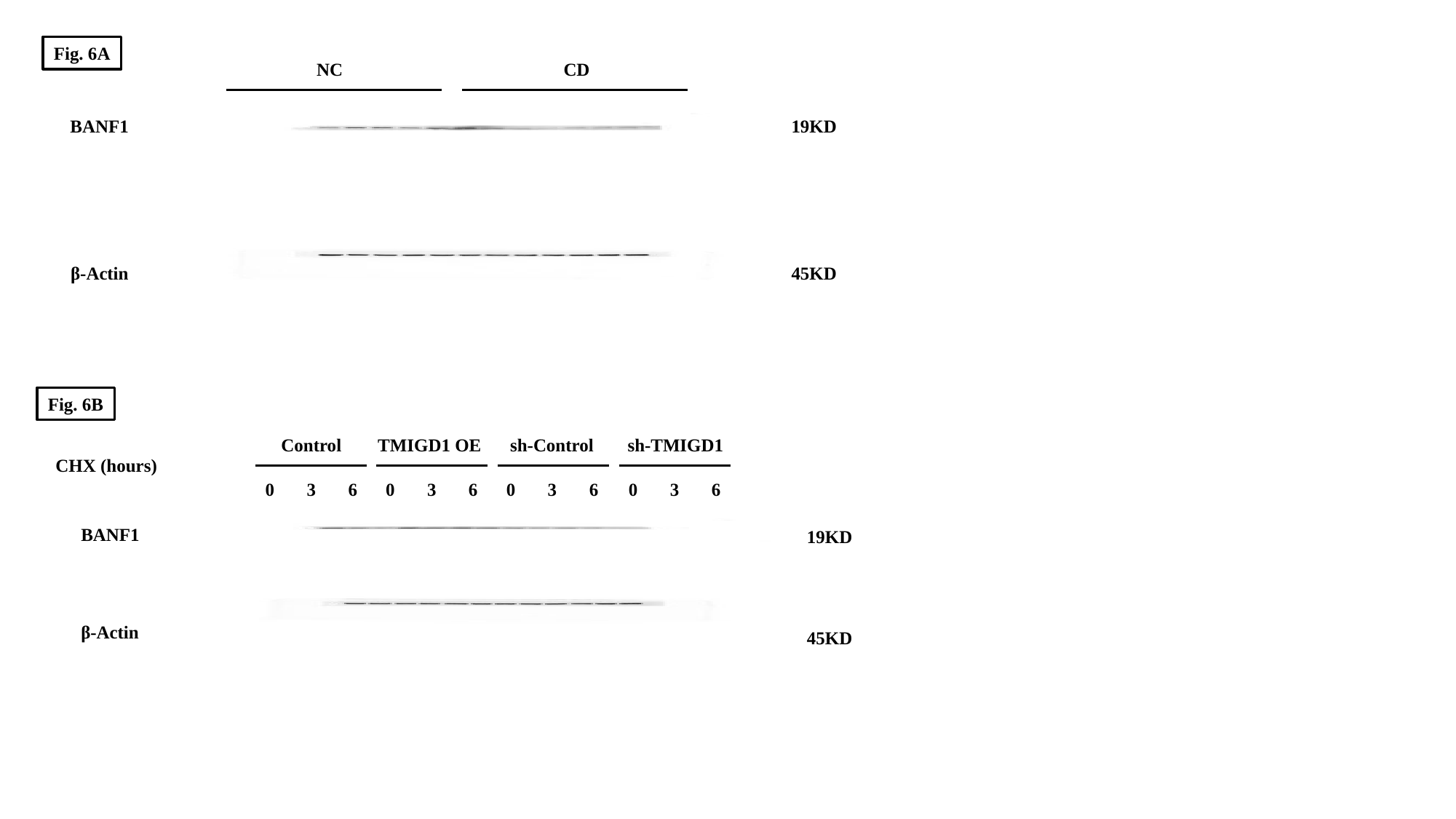

Fig. 6A
NC
CD
BANF1
19KD
β-Actin
45KD
Fig. 6B
Control
TMIGD1 OE
sh-Control
sh-TMIGD1
CHX (hours)
6
3
6
6
6
0
3
3
3
0
0
0
BANF1
19KD
β-Actin
45KD

## Slide 8
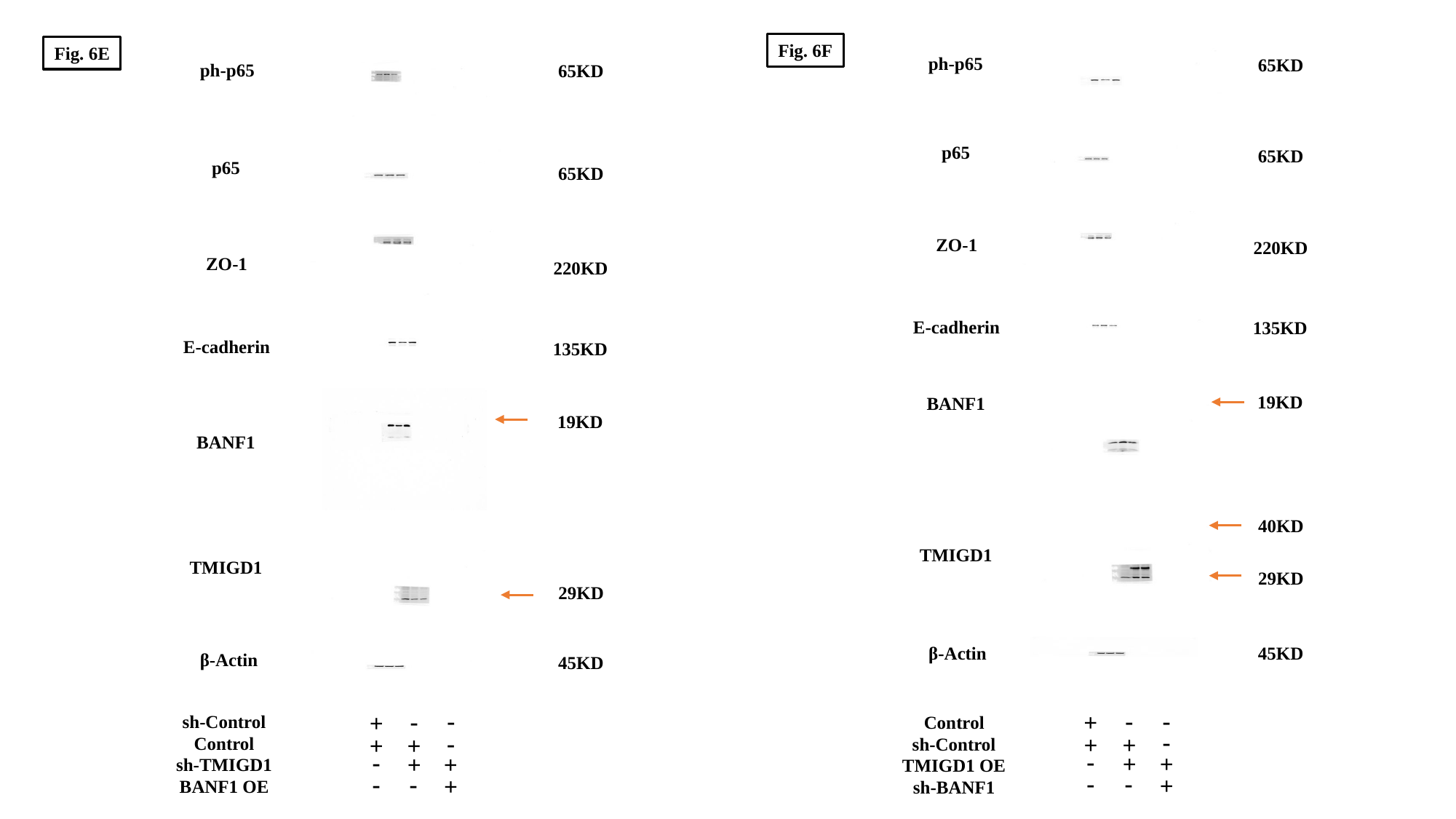

Fig. 6F
Fig. 6E
ph-p65
65KD
ph-p65
65KD
p65
65KD
p65
65KD
ZO-1
220KD
ZO-1
220KD
E-cadherin
135KD
E-cadherin
135KD
19KD
BANF1
19KD
BANF1
40KD
TMIGD1
TMIGD1
29KD
29KD
β-Actin
45KD
β-Actin
45KD
-
-
-
-
+
+
sh-Control
Control
sh-TMIGD1
BANF1 OE
Control
sh-Control
TMIGD1 OE
sh-BANF1
-
-
+
+
+
+
-
-
+
+
+
+
-
-
-
-
+
+

## Slide 9
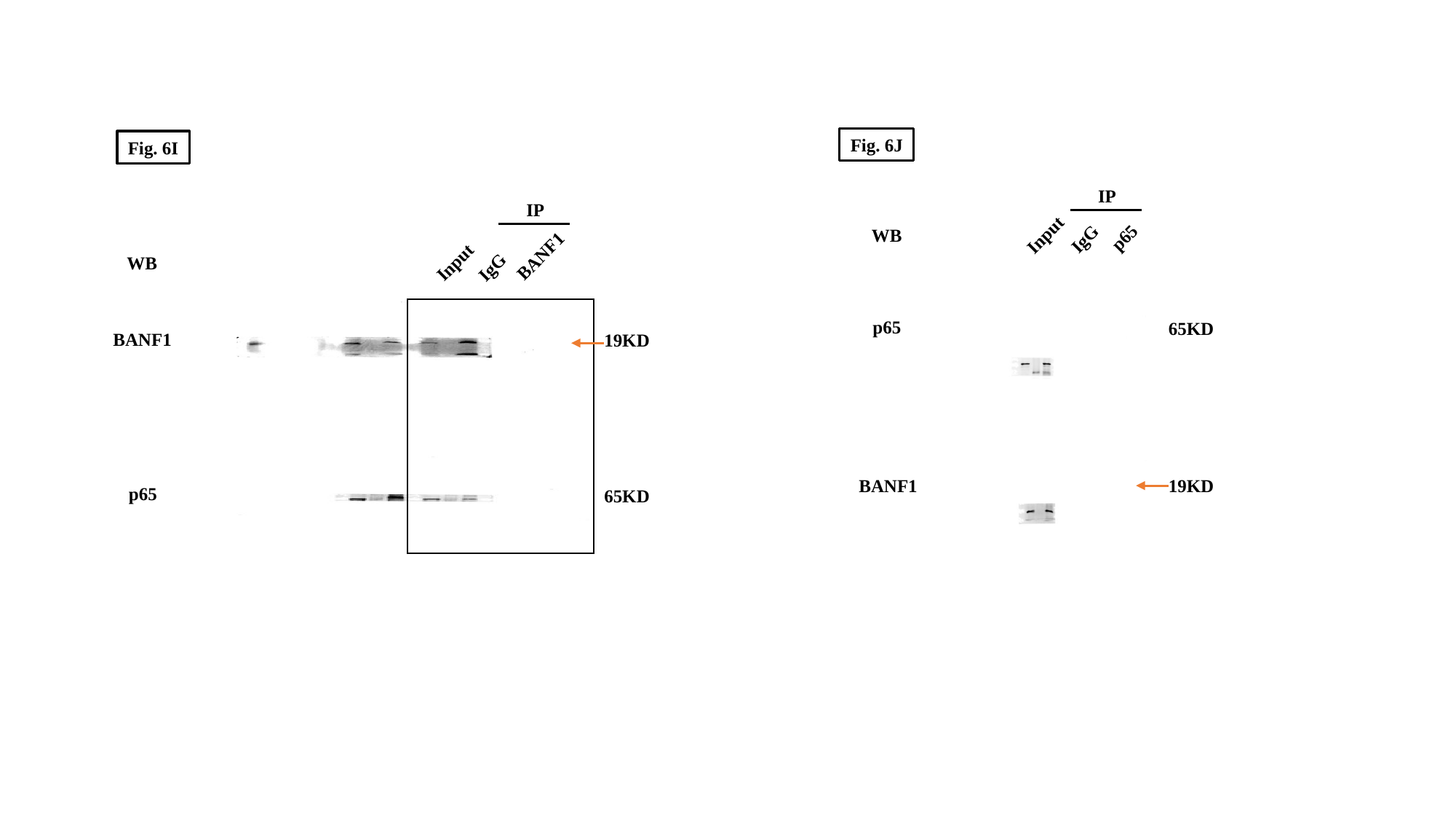

Fig. 6J
Fig. 6I
IP
IP
Input
WB
p65
IgG
BANF1
Input
WB
IgG
p65
65KD
BANF1
19KD
19KD
BANF1
p65
65KD

## Slide 10
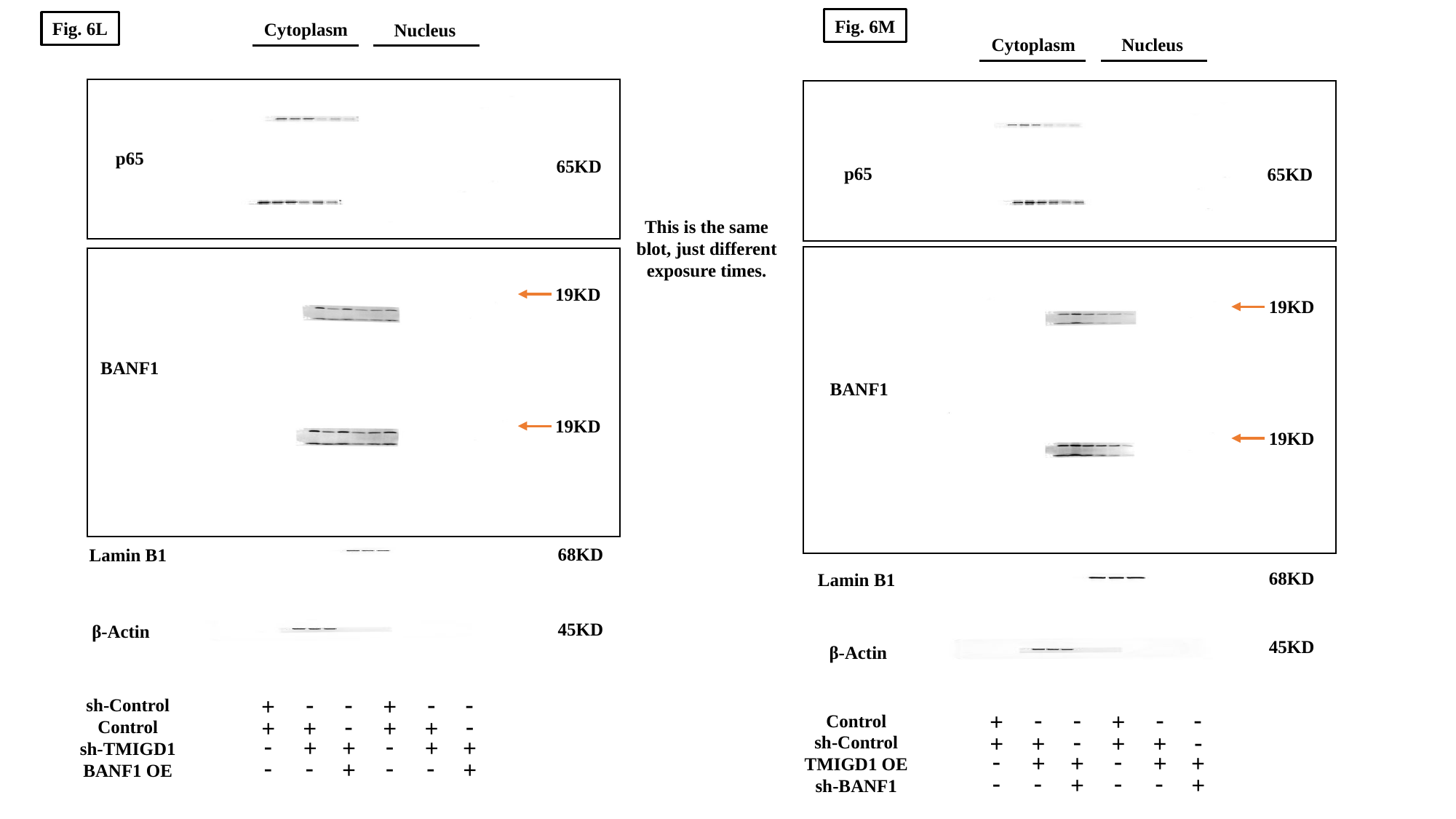

Fig. 6M
Fig. 6L
Cytoplasm
Nucleus
Cytoplasm
Nucleus
p65
65KD
p65
65KD
This is the same blot, just different exposure times.
19KD
19KD
BANF1
BANF1
19KD
19KD
68KD
Lamin B1
68KD
Lamin B1
45KD
β-Actin
45KD
β-Actin
-
-
-
-
+
+
sh-Control
Control
sh-TMIGD1
BANF1 OE
-
-
-
-
+
+
Control
sh-Control
TMIGD1 OE
sh-BANF1
-
-
+
+
+
+
-
-
+
+
+
+
-
-
+
+
+
+
-
-
+
+
+
+
-
-
-
-
+
+
-
-
-
-
+
+

## Slide 11
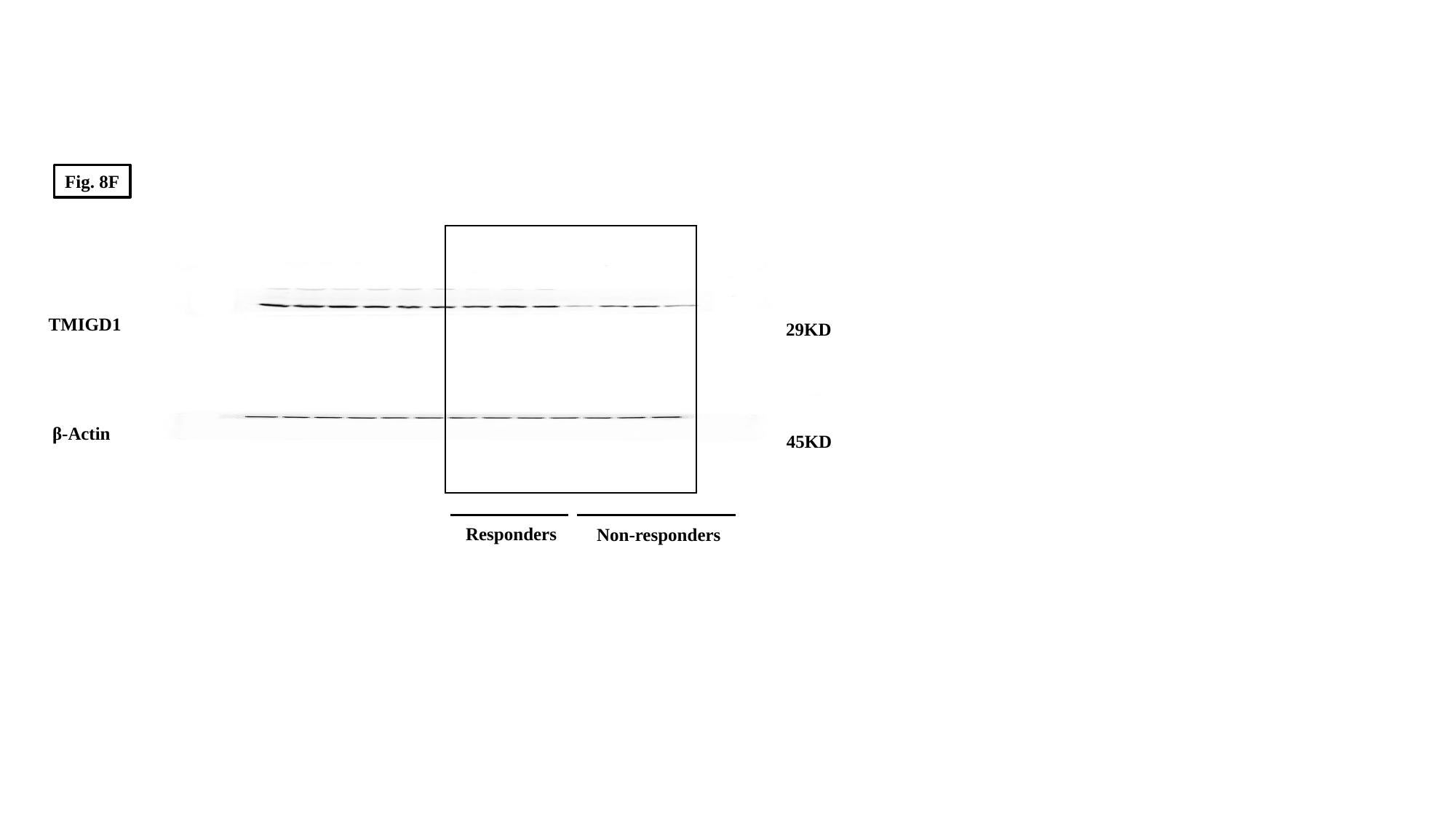

Fig. 8F
TMIGD1
29KD
β-Actin
45KD
Responders
Non-responders

## Slide 12
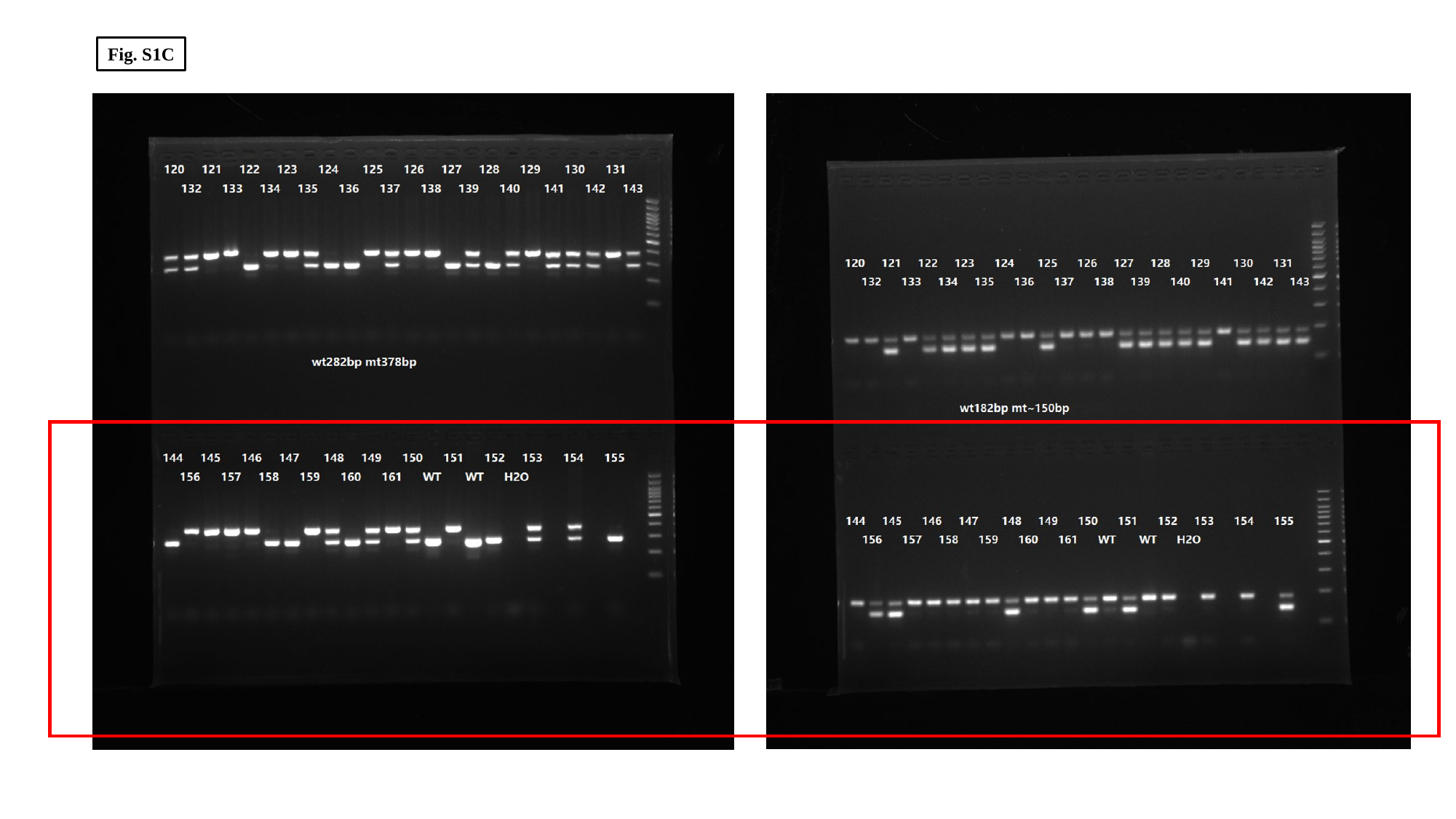

Fig. S1C

## Slide 13
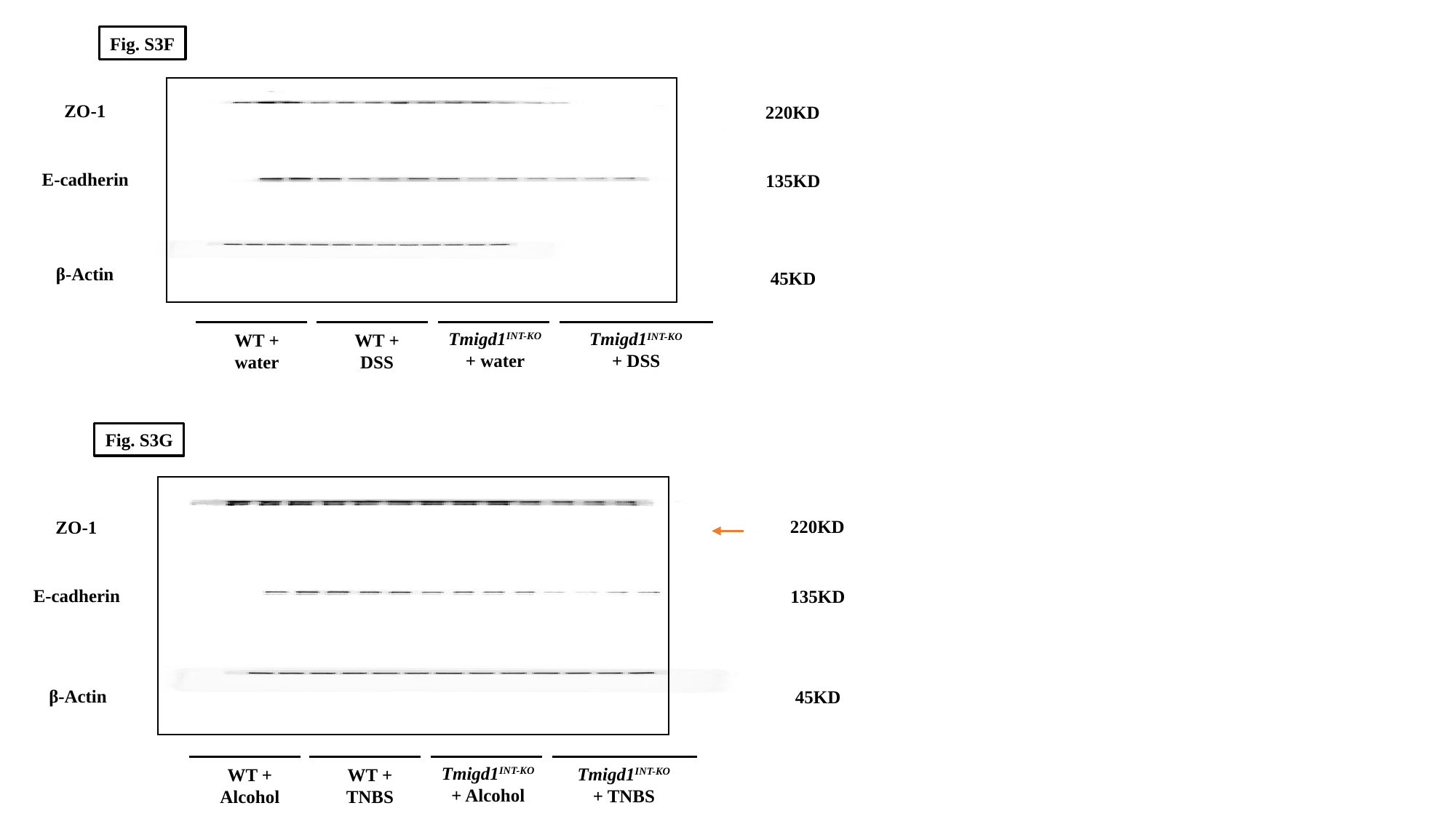

Fig. S3F
ZO-1
220KD
E-cadherin
135KD
β-Actin
45KD
Tmigd1INT-KO
+ water
Tmigd1INT-KO
+ DSS
WT +
water
WT +
DSS
Fig. S3G
220KD
ZO-1
E-cadherin
135KD
β-Actin
45KD
Tmigd1INT-KO
+ Alcohol
Tmigd1INT-KO
+ TNBS
WT +
Alcohol
WT +
TNBS

## Slide 14
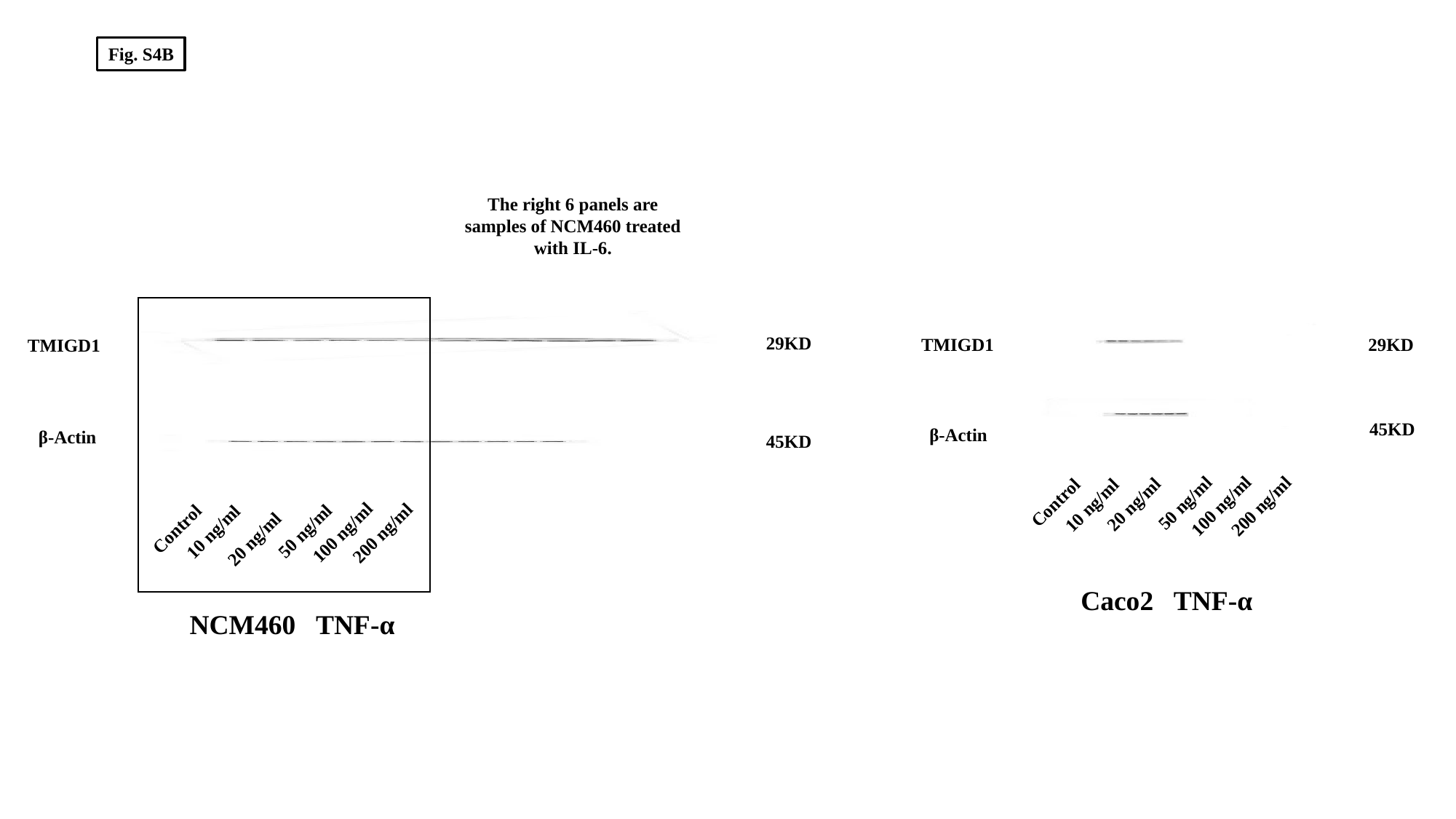

Fig. S4B
The right 6 panels are samples of NCM460 treated with IL-6.
29KD
TMIGD1
29KD
TMIGD1
45KD
β-Actin
β-Actin
45KD
Control
50 ng/ml
20 ng/ml
10 ng/ml
100 ng/ml
200 ng/ml
Control
50 ng/ml
10 ng/ml
100 ng/ml
200 ng/ml
20 ng/ml
Caco2 TNF-α
NCM460 TNF-α

## Slide 15
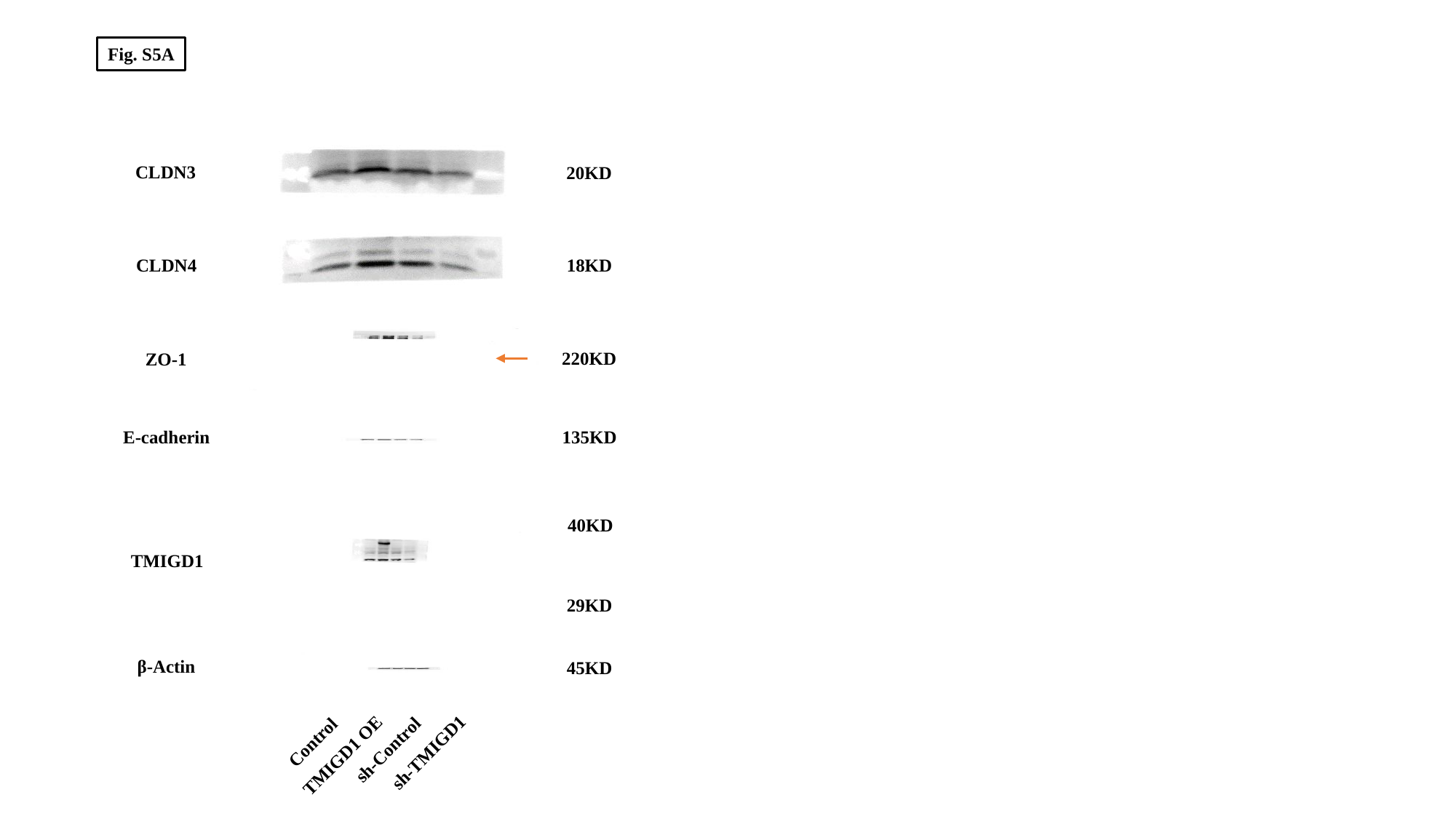

Fig. S5A
CLDN3
20KD
18KD
CLDN4
220KD
ZO-1
E-cadherin
135KD
40KD
TMIGD1
29KD
β-Actin
45KD
Control
sh-Control
sh-TMIGD1
TMIGD1 OE

## Slide 16
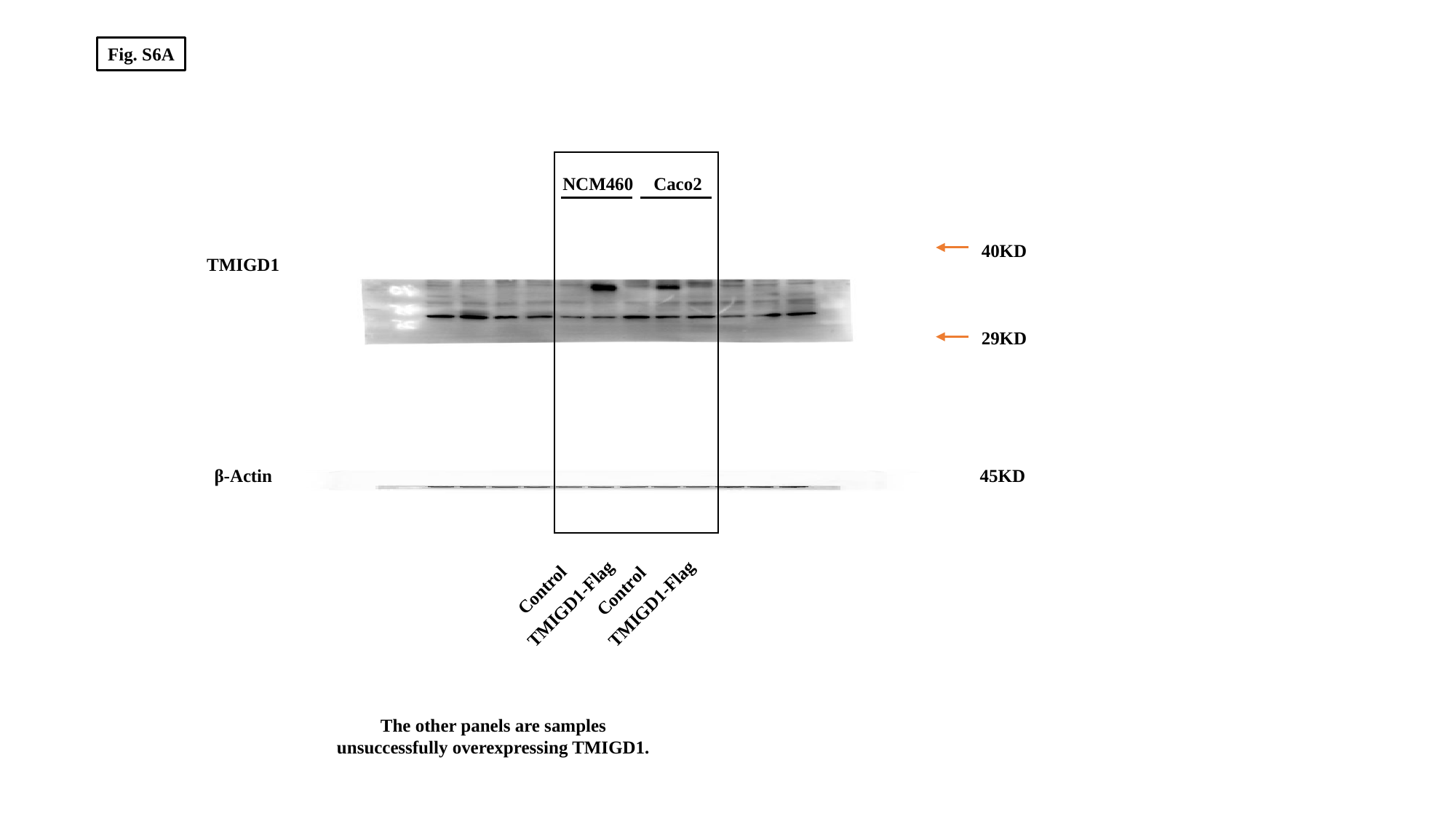

Fig. S6A
NCM460
Caco2
40KD
TMIGD1
29KD
β-Actin
45KD
Control
Control
TMIGD1-Flag
TMIGD1-Flag
The other panels are samples unsuccessfully overexpressing TMIGD1.

## Slide 17
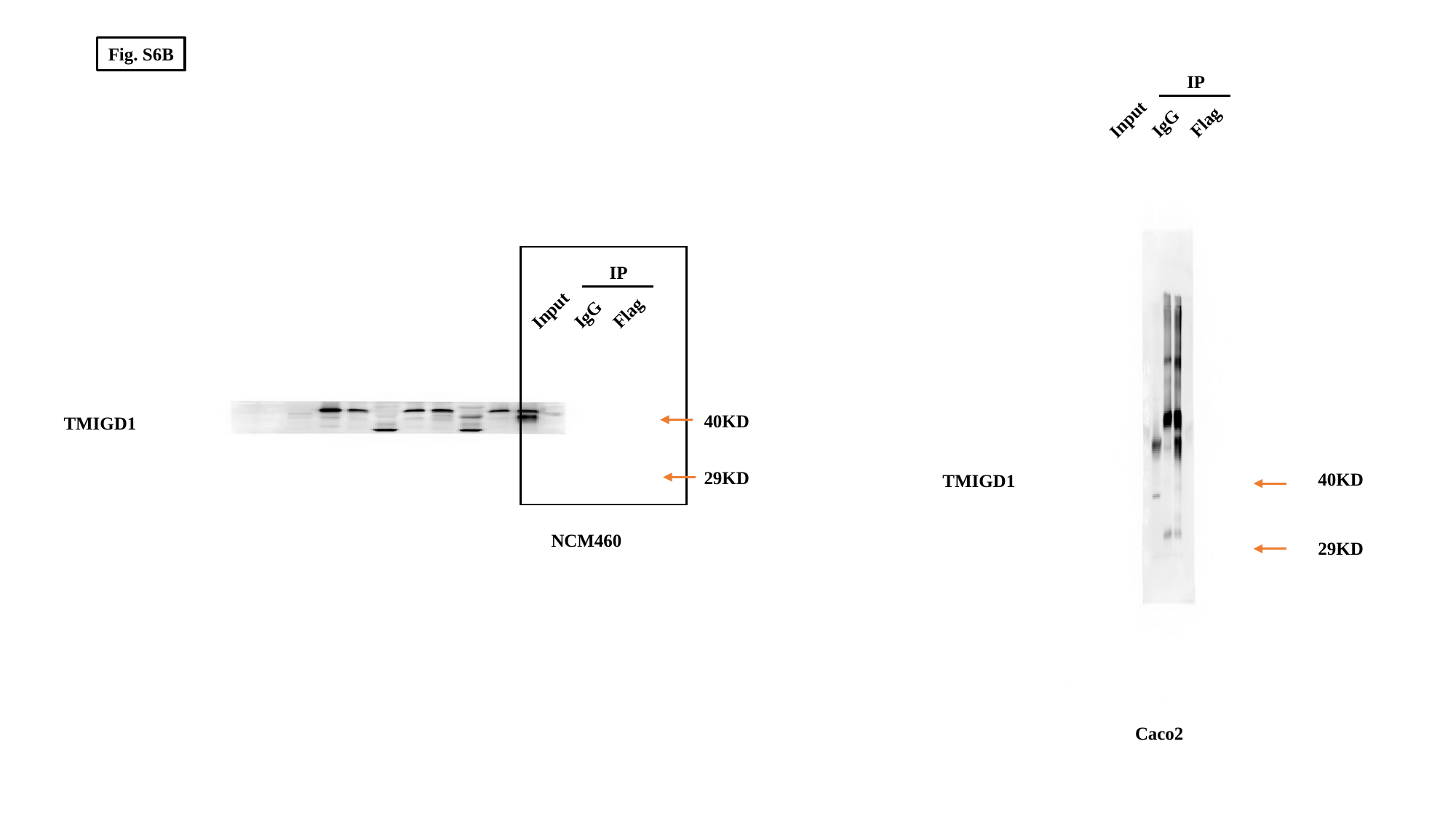

Fig. S6B
IP
Input
Flag
IgG
IP
Input
Flag
IgG
40KD
TMIGD1
29KD
40KD
TMIGD1
NCM460
29KD
Caco2

## Slide 18
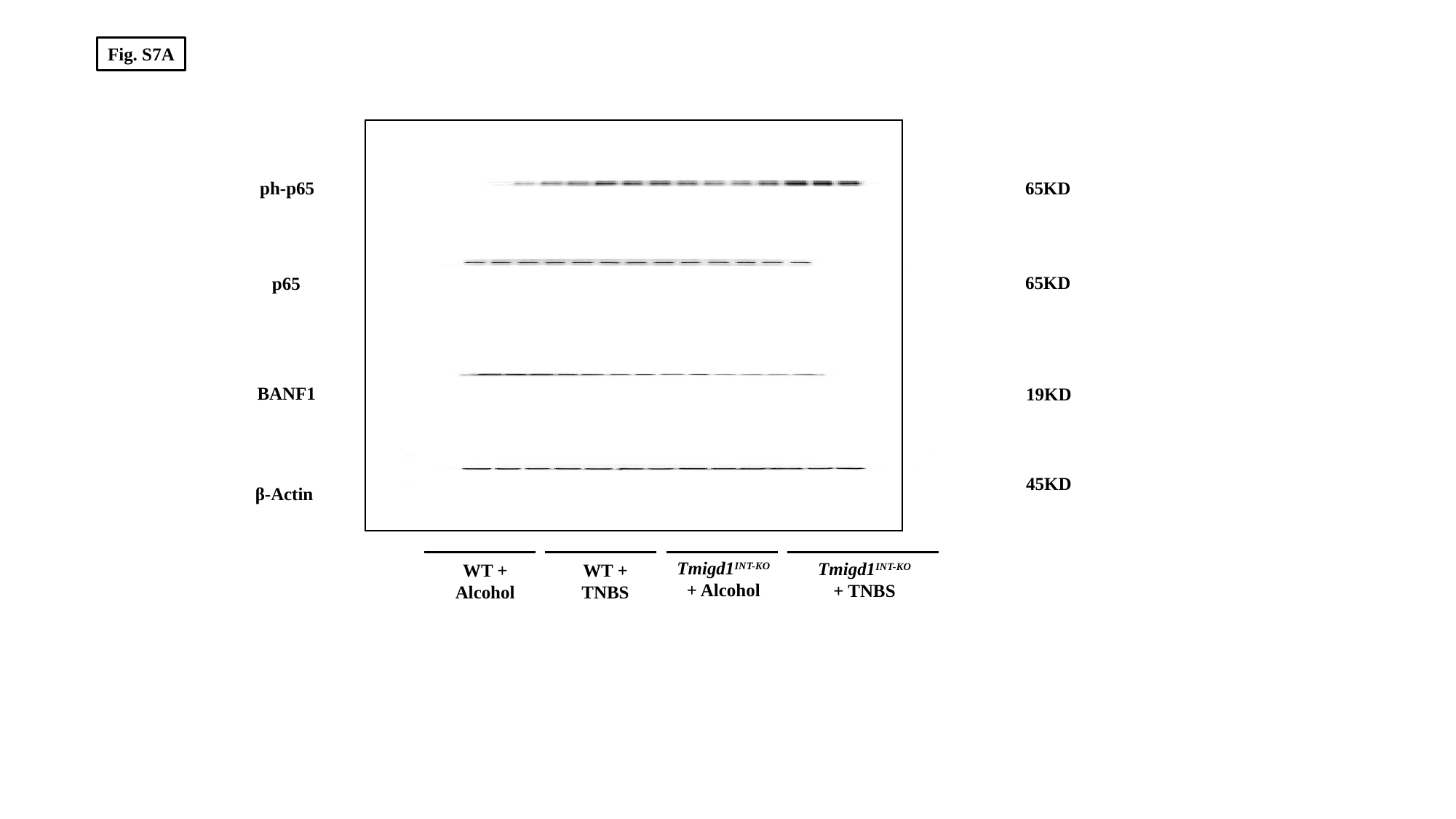

Fig. S7A
65KD
ph-p65
65KD
p65
BANF1
19KD
45KD
β-Actin
Tmigd1INT-KO
+ Alcohol
Tmigd1INT-KO
+ TNBS
WT +
Alcohol
WT +
TNBS

## Slide 19
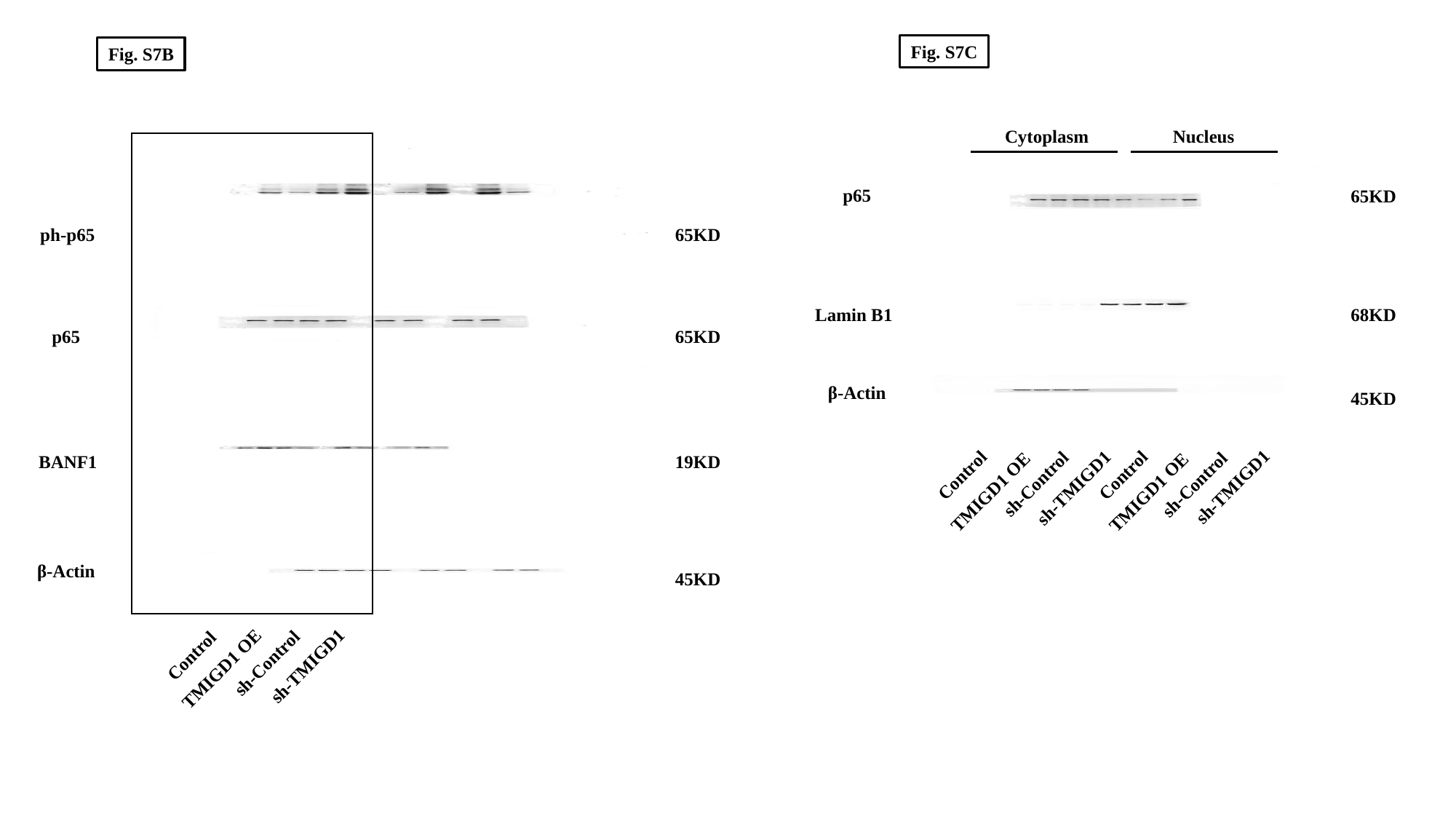

Fig. S7C
Fig. S7B
Nucleus
Cytoplasm
Nucleus
Cytoplasm
p65
65KD
ph-p65
65KD
68KD
Lamin B1
65KD
p65
β-Actin
45KD
19KD
BANF1
Control
Control
sh-Control
sh-Control
sh-TMIGD1
sh-TMIGD1
TMIGD1 OE
TMIGD1 OE
β-Actin
45KD
Control
sh-Control
sh-TMIGD1
TMIGD1 OE

## Slide 20
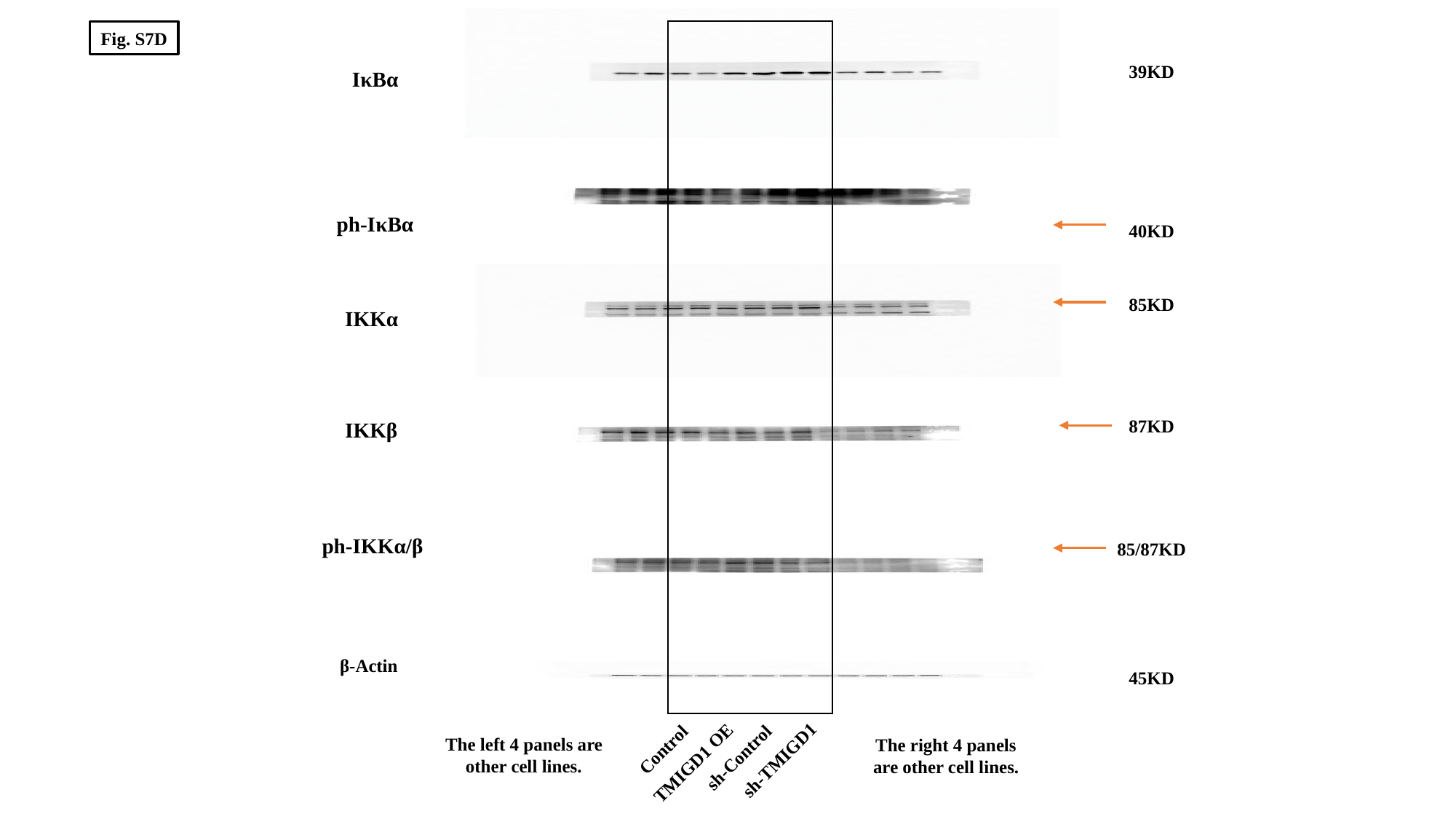

Fig. S7D
39KD
IκBα
ph-IκBα
40KD
85KD
IKKα
87KD
IKKβ
ph-IKKα/β
85/87KD
β-Actin
45KD
The left 4 panels are other cell lines.
The right 4 panels are other cell lines.
Control
sh-Control
sh-TMIGD1
TMIGD1 OE

## Slide 21
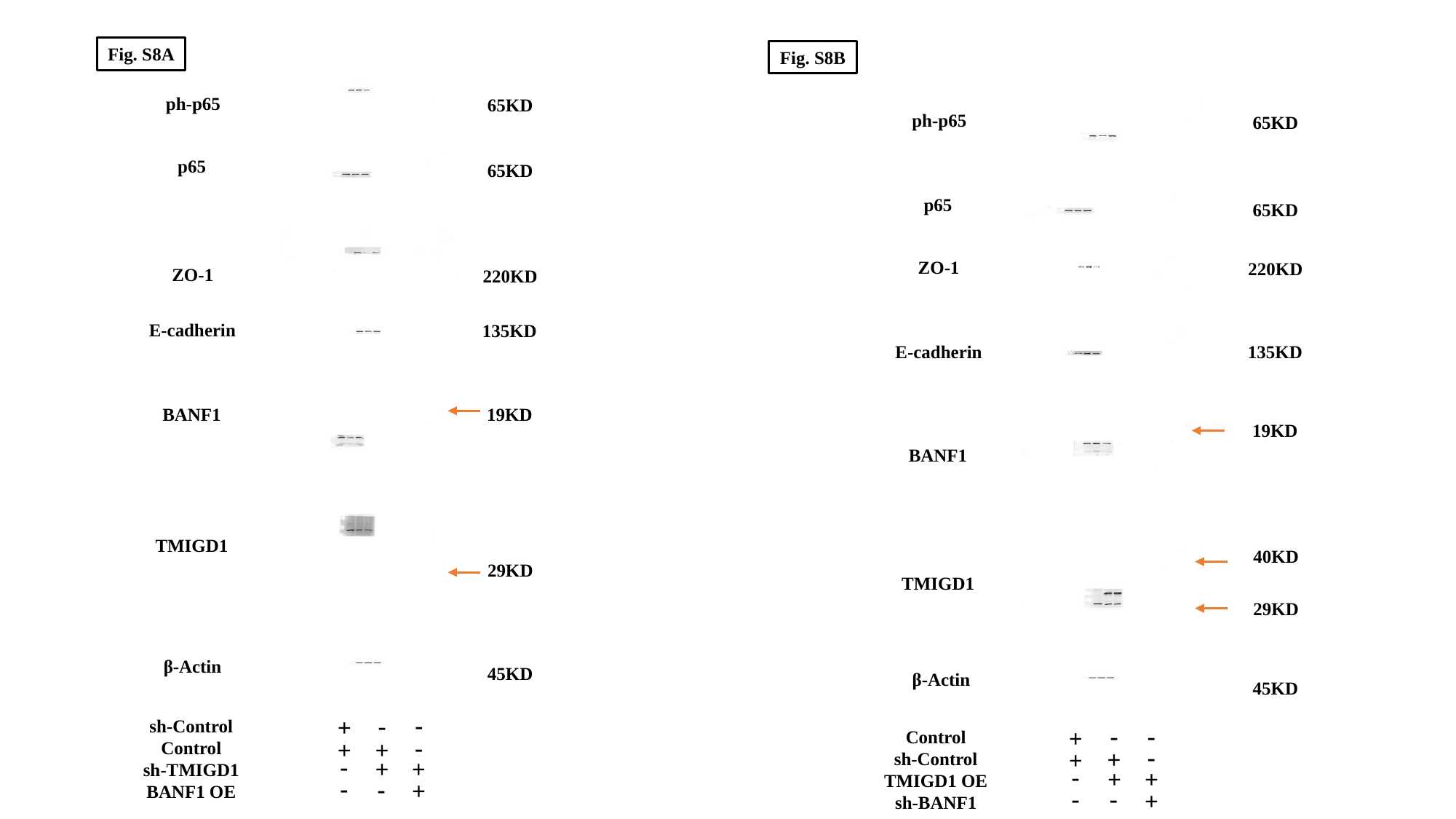

Fig. S8A
Fig. S8B
ph-p65
65KD
ph-p65
65KD
p65
65KD
p65
65KD
ZO-1
220KD
ZO-1
220KD
E-cadherin
135KD
135KD
E-cadherin
BANF1
19KD
19KD
BANF1
TMIGD1
40KD
29KD
TMIGD1
29KD
β-Actin
45KD
β-Actin
45KD
-
-
+
sh-Control
Control
sh-TMIGD1
BANF1 OE
-
-
+
Control
sh-Control
TMIGD1 OE
sh-BANF1
-
+
+
-
+
+
-
+
+
-
+
+
-
-
+
-
-
+

## Slide 22
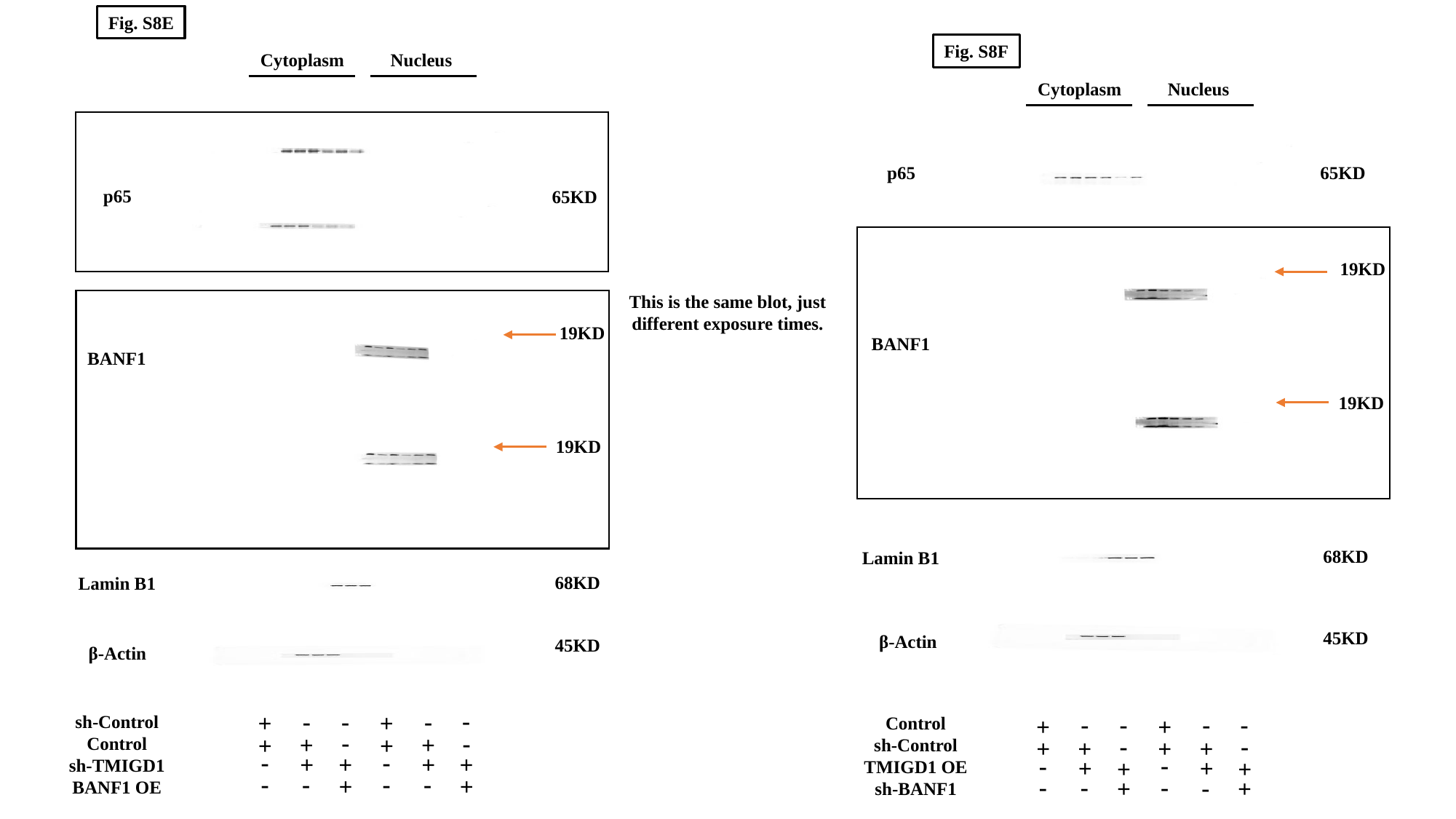

Fig. S8E
Fig. S8F
Cytoplasm
Nucleus
Cytoplasm
Nucleus
p65
65KD
p65
65KD
19KD
This is the same blot, just different exposure times.
19KD
BANF1
BANF1
19KD
19KD
68KD
Lamin B1
68KD
Lamin B1
45KD
β-Actin
45KD
β-Actin
-
-
-
-
+
+
-
-
-
-
sh-Control
Control
sh-TMIGD1
BANF1 OE
+
+
Control
sh-Control
TMIGD1 OE
sh-BANF1
-
-
+
+
+
+
-
-
+
+
+
+
-
-
+
+
+
+
-
-
+
+
+
+
-
-
-
-
+
+
-
-
-
-
+
+

## Slide 23
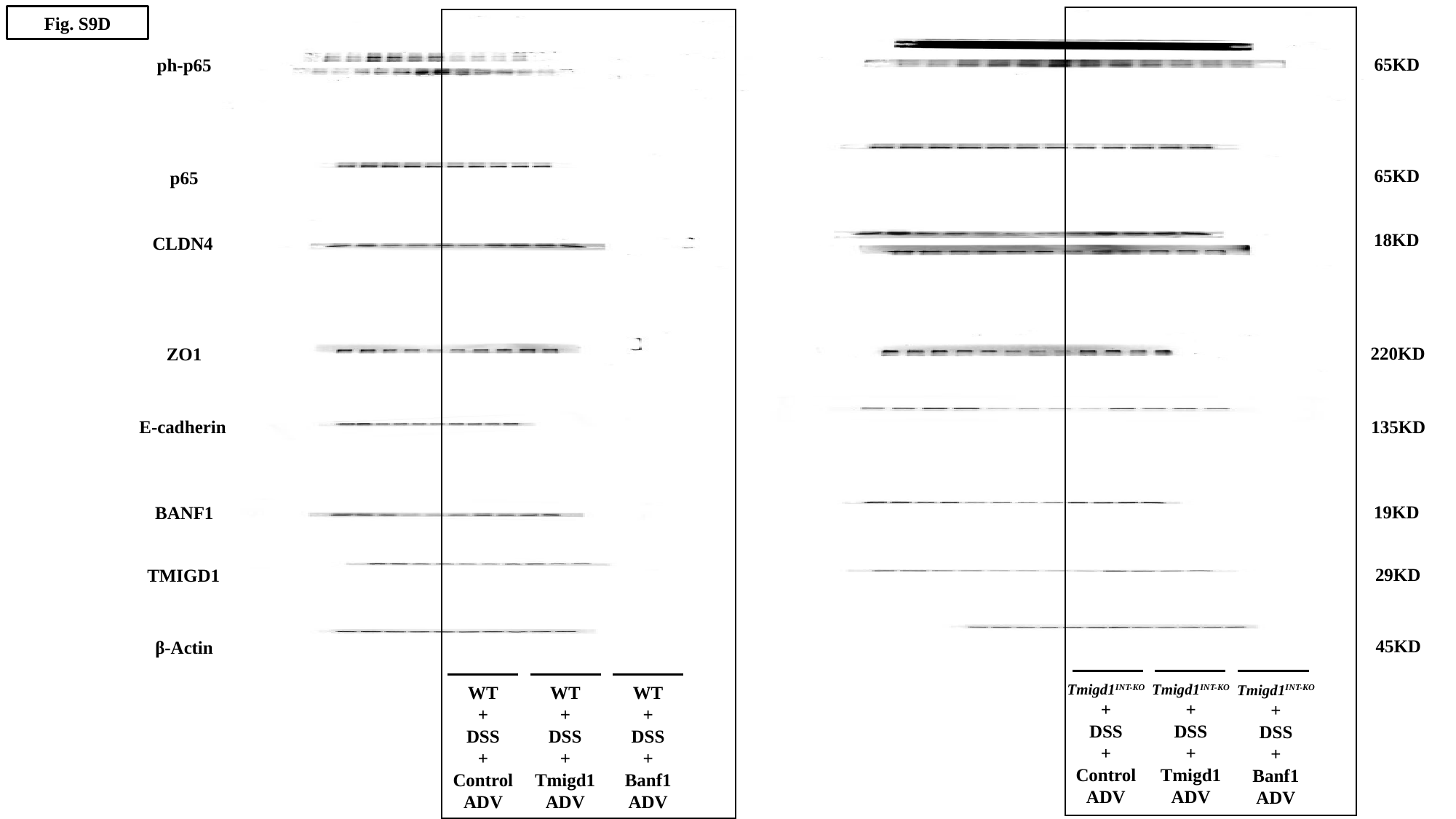

Fig. S9D
65KD
ph-p65
65KD
p65
18KD
CLDN4
220KD
ZO1
135KD
E-cadherin
19KD
BANF1
29KD
TMIGD1
45KD
β-Actin
Tmigd1INT-KO
+
DSS
+
Control
ADV
Tmigd1INT-KO
+
DSS
+
Tmigd1
ADV
Tmigd1INT-KO
+
DSS
+
Banf1
ADV
WT
+
DSS
+
Control
ADV
WT
+
DSS
+
Tmigd1
ADV
WT
+
DSS
+
Banf1
ADV
